# An integrative pharmacovigilance, network toxicology and molecular docking study on drug-induced cheilitis

**DOI:** 10.3389/fphar.2026.1757807

**Published:** 2026-03-20

**Authors:** Xuefeng Wang, Shangzhi Han, Yingxue Huang, Lei Zhang, Xue Li, Liqin Yang, Yanbo Zhang

**Affiliations:** 1 Department of Stomatology, The Affiliated Hospital of Chengde Medical University, Chengde, China; 2 Department of Thyroid Surgery, The Affiliated Hospital of Chengde Medical University, Chengde, China; 3 Department of Pharmacy, The Affiliated Hospital of Chengde Medical University, Chengde, China

**Keywords:** cheilitis, FAERS, molecular docking, network toxicology, pharmacovigilance

## Abstract

**Objective:**

Drug-induced cheilitis represents an inadequately comprehended adverse reaction, with limited understanding of its underlying mechanisms. This study employs an integrative approach, combining pharmacovigilance, network toxicology, and molecular docking methodologies, to systematically examine the phenomenon of drug-associated cheilitis.

**Methods:**

We conducted an analysis of 5,007 cheilitis reports obtained from the U.S. Food and Drug Administration (FDA) Adverse Event Reporting System (FDA Adverse Event Reporting System, 2004–2025) by employing disproportionality analysis and multivariate logistic regression techniques. Utilizing network toxicology, we constructed protein–protein interaction networks and identified enriched pathways. Furthermore, molecular docking and 500 ns molecular dynamics simulations were employed to validate the binding interactions between high-risk pharmacological agents and core molecular targets.

**Results:**

Thirty-eight pharmaceuticals demonstrated significant associations with cheilitis, with isotretinoin being the most frequently reported (ROR = 42.61) and crisaborole exhibiting the most pronounced signal (ROR = 550.48). Female sex emerged as an independent risk factor (OR = 0.771), whereas age and weight appeared to offer modest protective effects. Network analysis identified Interleukin 6 (IL6), tumor necrosis factor (TNF), AKT Serine/Threonine Kinase 1 (AKT1), Vascular Endothelial Growth Factor A (VEGFA) and Signal Transducer and Activator of Transcription 3 (STAT3) as central targets, with notable enrichment in the IL-17, TNF, and PI3K-Akt signaling pathways. Molecular docking studies indicated strong binding affinities (ranging from −8.1 to −6.2 kcal/mol), particularly for the afatinib-EGFR and capecitabine-IL-6 interactions. Molecular dynamics simulations confirmed the stability of these complexes, with MM/PBSA analysis highlighting key stabilizing residues. ADMET profiling predicted a high risk of drug-induced liver injury for four compounds, while lamotrigine demonstrated a favorable safety profile.

**Conclusion:**

This integrative framework connects population-level indicators with mechanistic forecasts, providing a translational model for comprehending, predicting, and managing drug-induced cheilitis.

## 1 Introduction

Cheilitis, an inflammatory condition of the vermilion lip and its surrounding skin, poses a significant clinical challenge due to its complex etiology, which encompasses environmental triggers like ultraviolet radiation, drug-induced reactions, and microbial infections (Narayanan and Rogge, 2024; Bernaola et al., 2023; Huoh and Chang, 2013). The global prevalence of cheilitis is notable, and its pathophysiological landscape involves an intricate interplay of immune dysregulation, oxidative stress, and epidermal barrier disruption (Lugović-Mihić et al., 2018; Carneiro et al., 2023). Despite its clinical importance, the precise molecular mechanisms, particularly for drug-induced cases, remain inadequately defined, often leading to empiric management and suboptimal patient outcomes.

Traditional approaches to investigating cheilitis, such as clinical observational studies and conventional toxicological models, struggle to systematically identify risk factors and elucidate the underlying molecular pathways on a systems level. This gap underscores the necessity for innovative, integrative research strategies that can leverage large-scale real-world data and advanced computational biology tools.

The integation of multiple methodologies offers a transformative paradigm. First, the U.S. Food and Drug Administration Adverse Event Reporting System (FAERS) database serves as a powerful tool for pharmacovigilance, enabling the detection of potential adverse event signals associated with pharmaceutical interventions (Kang et al., 2025; Wu et al., 2025). Second, logistic regression analysis complements this by quantifying the impact of specific clinical and demographic variables (e.g., gender, concomitant medications) on cheilitis risk, allowing for the identification and adjustment of potential confounders (Chen et al., 2025). Third, network toxicology provides a systems-level perspective, constructing interactive networks among compounds, protein targets, and biological pathways (Xu et al., 2025a). This approach can predict key targets (e.g., IL-6, STAT3, TNF) and pathways involved in cheilitis, moving beyond a single-target paradigm to reveal a holistic mechanistic picture.

Although integrative pipelines have been used for other dermatological ADRs like Stevens-Johnson syndrome and drug-induced rash (Schotland et al., 2016), cheilitis is less understood. The lip epithelium’s unique properties suggest drug-induced injury may involve different pathways than general skin inflammation. This study seeks to adapt a proven methodology to address this gap in oral mucosal toxicology.

Crucially, the hypothesized interactions between potential causative compounds and key disease targets derived from network toxicology require experimental validation. This is where molecular docking, a key computational structure-based method, plays an indispensable role (Xu et al., 2025b). Molecular docking simulates the binding mode and affinity between a small molecule (e.g., a drug suspected to induce cheilitis) and a protein target (e.g., an inflammatory cytokine or receptor identified via network toxicology) (He et al., 2025; Feng et al., 2025). By evaluating the binding energy and analyzing the intermolecular interactions at the atomic level, molecular docking can provide structural insights into potential drug-target interactions, thereby generating testable hypotheses to bridge the gap between statistical association and mechanistic understanding (Yoo et al., 2025; Ghosh et al., 2025).

This study introduces a novel integrative framework that synergizes FAERS data mining, logistic regression, network toxicology, and molecular docking. Our objectives are fourfold: (Narayanan and Rogge, 2024): to identify and characterize significant drug-cheilitis associations through systematic mining of the FAERS database (Bernaola et al., 2023); to quantify key clinical and demographic risk factors using multivariate logistic regression (Huoh and Chang, 2013); to construct a comprehensive protein-protein interaction network and identify central targets and enriched biological pathways pertinent to cheilitis pathogenesis via network toxicology; and (Lugović-Mihić et al., 2018) to validate the binding interactions between the top predicted causative drugs and the core disease targets using molecular docking, thereby providing atomistic insights into the potential molecular initiating events.

This cohesive strategy establishes a translational pipeline that progresses from population-level signal detection and risk quantification to systems-based mechanistic prediction and, finally, to structure-level mechanistic validation. By synthesizing evidence from pharmacoepidemiology, biostatistics, systems biology, and computational structural biology, this research aims to generate a unified and actionable understanding of cheilitis, ultimately informing improved risk stratification and the development of targeted therapeutic strategies.

## 2 Materials and methods

### 2.1 Data sources and processing

**FIGURE 1:**
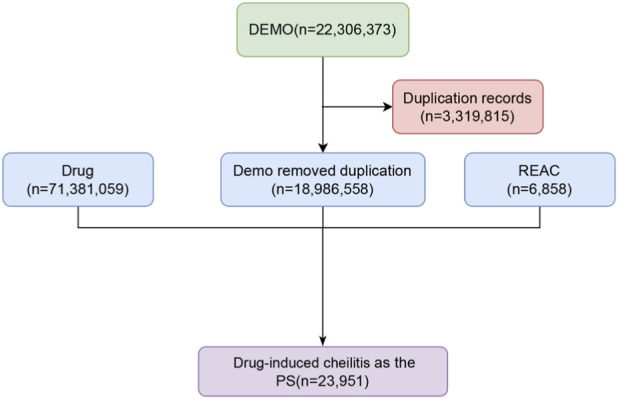
The flowchart for identifying drug-induced cheilitis using the FDA Adverse Event Reporting System (FAERS) database.

Case reports of cheilitis as an adverse drug reaction (ADR) were isolated from the FAERS database (Q1 2004 - Q2 2025). The initial dataset was refined by excluding non-drug-related events, duplicates, and entries with incomplete or ambiguous documentation (Supplementary Table S1). This curation process yielded a final cohort specifically for investigating drug-induced cheilitis (Figure 1). Subsequently, demographic characteristics (e.g., age, gender), reporting information (e.g., year, country), and pertinent drug details were extracted from the curated reports (Ahdi et al., 2023; Zhang et al., 2023).

### 2.2 Disproportionality analysis in the context of pharmacovigilance

Disproportionality analyses utilizing the proportional reporting ratio (PRR), reporting odds ratio (ROR), and empirical Bayes geometric mean (EBGM) were performed to pinpoint signals of adverse drug events (ADEs), defined as statistically significant drug-ADE associations (Supplementary Table S2) (Go et al., 2025). Furthermore, subgroup analyses stratified by age and sex were conducted to identify medications linked to cheilitis within specific demographic cohorts. In subgroup analyses stratified by age, sex, and weight, only subgroups containing at least three cases were included to enhance the stability of disproportionality estimates.

### 2.3 Sensitivity analysis

To evaluate the robustness of our primary pharmacovigilance findings and address potential biases arising from missing data, we conducted a sensitivity analysis using a complete-case dataset. In primary pharmacovigilance analyses, the inclusion of reports with unknown demographic information, such as age, sex, and weight, can introduce noise or obscure true signals within specific subpopulations. Consequently, we excluded all case reports where data on age, sex, or weight were categorized as “unknown” or missing. After this data cleaning process, we re-conducted the disproportionality analyses—namely, the Reporting Odds Ratio (ROR), Proportional Reporting Ratio (PRR), and Empirical Bayes Geometric Mean (EBGM)—stratified by age (<19, 19–44, 45–59, ≥60 years), sex (male, female), and weight (<50 kg, 50–100 kg, >100 kg). Consistent with the primary analysis, only subgroups containing a minimum of three case reports for a given drug were included to ensure the stability of the estimates. We then assessed the consistency of signal patterns between the full dataset (including unknowns) and the complete-case dataset to validate the reliability of our conclusions.

### 2.4 Elements influencing cheilitis caused by drugs

Potential risk factors for drug-induced cheilitis, encompassing demographic variables (age, sex, weight), the top 20 prevalent comorbidities, and concomitant medications, were screened from eligible reports after excluding records with missing data. Associations between these exposures and cheilitis were ascertained through univariate and subsequent multivariate logistic regression, with odds ratios (ORs) calculated to quantify risk. A stepwise regression approach (P < 0.05) was employed to select covariates for the final model (Zhou et al., 2023; Jing et al., 2022).

### 2.5 Network toxicology analysis

A network toxicology approach was implemented to decipher the systemic molecular mechanisms of drug-induced cheilitis (Yang et al., 2025; Wang et al., 2025).

#### 2.5.1 Target collection

Potential targets of the causative drugs identified in Section 2.2 were retrieved from the Comparative Toxicogenomics Database (CTD; https://ctdbase.org/), PharmMapper (http://www.lilab-ecust.cn/pharmmapper/), and STITCH (http://stitch.embl.de/). Disease-associated genes for “cheilitis” and related inflammatory lip disorders were collected from GeneCards (https://www.genecards.org/) and DisGeNET (https://www.disgenet.org/). Only targets with a prediction score ≥0.7 (CTD/STITCH) or experimental evidence (GeneCards) were retained. Non-human and duplicate entries were removed.

#### 2.5.2 Network construction

Protein-protein interaction (PPI) data for the collected targets were obtained from the STRING database (https://string-db.org/) using a confidence score cutoff ≥0.4. The PPI network was visualized and analyzed in Cytoscape (version 3.9.1). Hub targets were identified based on degree centrality ≥10, betweenness centrality, and closeness centrality.

#### 2.5.3 Functional enrichment analysis

Hub targets were subjected to Gene Ontology (GO) and Kyoto Encyclopedia of Genes and Genomes (KEGG) pathway enrichment using the clusterProfiler R package (version 4.2.2). Enrichment significance was assessed via hypergeometric test, with Benjamini–Hochberg correction for multiple testing. Terms with FDR <0.05 and containing at least 5 genes were considered significantly enriched. GO analysis covered biological process (BP), cellular component (CC), and molecular function (MF). Kyoto Encyclopedia of Genes and Genomes (KEGG) pathway enrichment was performed in parallel under the same statistical criteria.

#### 2.5.4 Workflow visualization

**FIGURE 2:**
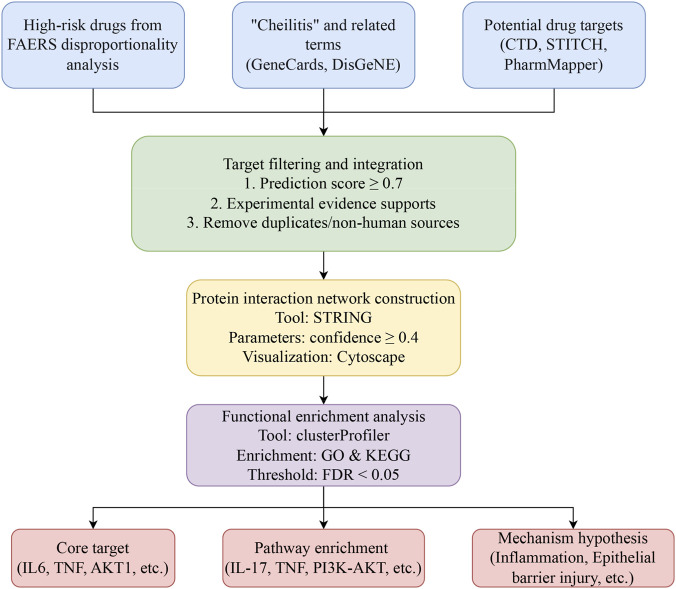
Workflow of network toxicology analysis for drug-induced cheilitis.

A detailed schematic of the network toxicology pipeline is provided in Figure 2, illustrating the sequence from data retrieval to functional interpretation.

### 2.6 Molecular docking

AutoDock Vina 1.2.2 was utilized to carry out molecular docking. Clinically relevant high-risk drugs associated with cheilitis were selected based on disproportionality and network toxicology analyses. Protein targets were chosen from core proteins within inflammatory pathways enriched in the network analysis.

#### 2.6.1 Preparation of ligands

The three-dimensional structures of the selected drugs (afatinib, capecitabine, everolimus, isotretinoin, and lamotrigine) were retrieved in SDF format from the PubChem database. Ligands were prepared using Open Babel (version 3.1.1) by adding hydrogen atoms, assigning Gasteiger partial charges, and optimizing protonation states at physiological pH (7.4). The resulting structures were converted to PDBQT format using AutoDock Tools (ADT) for docking input.

#### 2.6.2 Preparation of protein targets

The crystal structures of target proteins—EGFR (PDB: 2EB2), IL-6 (PDB: 1ALU), TNF-α (PDB: 1A8M), and BCL-2 (PDB: 1G5M)—were obtained from the Protein Data Bank. Structures were prepared by removing water molecules, co-crystallized ligands, and non-essential ions. Missing hydrogen atoms were added, and Kollman united-atom charges were assigned using AutoDock Tools. The binding site for each protein was defined based on the coordinates of the native ligand or known active site residues, and a grid box of dimensions 30 × 30 × 30 Å was centered on this region.

It is important to acknowledge that molecular docking serves as a computational predictive tool, generating hypotheses concerning potential binding modes and affinities. However, these predictions necessitate subsequent experimental validation.

### 2.7 Molecular dynamics simulations

Molecular dynamics (MD) simulations were conducted utilizing GROMACS version 2022.2. The topological parameters of the protein and ligand were independently assessed for the protein-ligand complex. The protein was modeled employing the Amber14sb force field, while the ligand was modeled using the General Amber Force Field (GAFF), incorporating restrained electrostatic potential (RESP) charges. The RESP charges for the ligand were calculated using ORCA version 6.0.0 and Multiwfn version 3.8 (dev). The protein-ligand complex was assembled by merging the topology and coordinate files of the protein and ligand. Subsequently, it was positioned at the center of an octahedral simulation box, ensuring a minimum distance of 1 nm between the protein and the box boundaries. The system was solvated with TIP3P water molecules, and Na+/Cl− ions were introduced to neutralize the system’s charge by replacing an appropriate number of water molecules. Energy minimization was conducted utilizing the steepest descent method. Subsequent equilibration of the system was achieved under NVT (constant number of particles, volume, and temperature) and NPT (constant number of particles, pressure, and temperature) ensembles for a duration of 200 picoseconds, employing a time step of 2 femtoseconds, while implementing position restraints. Periodic boundary conditions were applied to confine the system within the simulation box, and the enhanced Berendsen coupling algorithm was employed to maintain the system temperature at 300 K. Covalent bonds were constrained using the LINCS algorithm, and long-range electrostatic interactions were computed via the particle mesh Ewald method. Following equilibration in both NVT and NPT ensembles, a 500-nanosecond molecular dynamics simulation was executed on the protein-ligand complex without position restraints, with snapshots recorded every 10 picoseconds. Conformational analysis was performed using PyMOL for visualization and VMD for trajectory analysis.

To quantitatively assess the binding free energies between identified high-risk drugs and their respective protein targets, the Molecular Mechanics/Poisson–Boltzmann Surface Area (MM/PBSA) method was utilized via the gmx_MMPBSA tool. This method is widely acknowledged for its robustness in estimating interaction energies of protein–ligand complexes based on MD trajectories. For each complex, snapshots were extracted from the equilibrated phase of a 500 ns MD simulation trajectory to ensure conformational stability and representativeness. The binding free energy was decomposed into individual energy components, including van der Waals, electrostatic, polar solvation, and non-polar solvation contributions, providing a detailed energetic landscape of the interactions. This analysis offers a quantitative evaluation of the thermodynamic stability of the complexes and complements the structural insights derived from molecular docking and MD simulations.

### 2.8 ADMET analysis

ADMET properties of five compounds (Afatinib, Capecitabine, Everolimus, Isotretinoin, and Lamotrigine) were predicted *in silico* using ADMETlab 3.0 (https://admetlab3.scbdd.com/server/screening), a robust platform incorporating validated machine learning and quantitative structure-activity relationship (QSAR) algorithms. Canonical SMILES of each compound served as input, with structural integrity verified prior to analysis. Under default parameters, 89 key endpoints were evaluated, encompassing absorption (e.g., human intestinal absorption, Caco-2/MDCK permeability, transporter interactions), distribution (e.g., plasma protein binding, logVDss, BBB penetration), metabolism (e.g., CYP enzyme inhibition/substrate profiles), excretion (e.g., intrinsic clearance, half-life), and toxicity (e.g., hERG liability, Ames test, DILI). Predicted data were extracted directly without additional statistical transformations, leveraging the platform’s established validation against large datasets (concordance rate >85% for key endpoints) to ensure reliability.

## 3 Results

### 3.1 Attributes of drug-related cheilitis

A total of 5,007 cases of drug-induced cheilitis were identified from the FAERS database spanning from 2004 to 2025. The annual distribution of reports demonstrated a general increasing trend, peaking in 2013 (n = 353, 7.05%) and 2024 (n = 321, 6.41%), with a notable decline in 2025 due to partial data availability (Table 1).

**TABLE 1:** Attributes of drug-induced cheilitis (2004–2025).

Variable	n (%)
Year	​
2004	95 (1.90)
2005	122 (2.44)
2006	132 (2.64)
2007	127 (2.54)
2008	142 (2.84)
2009	156 (3.12)
2010	198 (3.95)
2011	221 (4.41)
2012	304 (6.07)
2013	353 (7.05)
2014	277 (5.53)
2015	315 (6.29)
2016	302 (6.03)
2017	290 (5.79)
2018	296 (5.91)
2019	277 (5.53)
2020	244 (4.87)
2021	215 (4.29)
2022	268 (5.35)
2023	280 (5.59)
2024	321 (6.41)
2025	72 (1.44)
Sex	​
Female	3,138 (62.67)
Male	1,539 (30.74)
Unknown	330 (6.59)
Age	55.00 (34.00,68.00)
<19	490 (9.91)
19∼44	742 (15.01)
44∼59	815 (16.49)
≥60	1,573 (31.82)
Unknow	1,323 (26.77)
Weight	67.11 (56.25,82.00)
<50	246 (4.92)
50∼100	1,332 (26.65)
>100	114 (2.28)
Unknow	3,307 (66.15)
Reporter	​
Consumer	2,158 (43.10)
Physician	1,160 (23.17)
Other health-professional	696 (13.90)
Pharmacist	504 (10.07)
Unknown	306 (6.11)
Lawyer	178 (3.56)
Registered nurse	5 (0.10)
Reported countries	​
United States	2,299 (63.49)
France	328 (9.06)
Japan	161 (4.45)
Canada	131 (3.62)
Other	591 (16.31)
United Kingdom	111 (3.07)
Outcomes	​
Other serious	2036 (53.79)
Hospitalization	1,297 (34.27)
Death	150 (3.96)
Life threatening	144 (3.80)
Disability	127 (3.36)
Required intervention to prevent permanent impairment/Damage	28 (0.74)
Congenital anomaly	3 (0.08)
TTO	15.00 (2.00,64.00)
<2	728 (14.75)
2∼5	284 (5.75)
5∼7	122 (2.47)
7∼14	315 (6.38)
14∼28	343 (6.95)
≥28	841 (17.04)
Unknow	2,303 (46.66)

Females accounted for the majority of cases (62.67%), with males representing 30.74% of reports. The median age of affected individuals was 55.0 years (IQR: 34.0–68.0), and patients aged ≥60 years constituted the largest age subgroup (31.82%). Weight data were largely incomplete (66.15% unknown), but among those reported, the median weight was 67.11 kg (IQR: 56.25–82.00).

Consumers were the most frequent reporters (43.10%), followed by physicians (23.17%). Geographically, the United States contributed the majority of reports (63.49%), with France (9.06%) and Japan (4.45%) as other notable contributors. Regarding clinical outcomes, “other serious” events were most common (53.79%), followed by hospitalization (34.27%). Life-threatening events and death were reported in 3.80% and 3.96% of cases, respectively.

The median time-to-onset (TTO) of cheilitis was 15 days (IQR: 2–64). A substantial proportion of cases (46.66%) lacked TTO data. Among those with available data, 14.75% of cases occurred within 2 days of drug initiation, and 17.04% had a TTO exceeding 28 days.

### 3.2 Characteristics of medications associated with cheilitis

**FIGURE 3:**
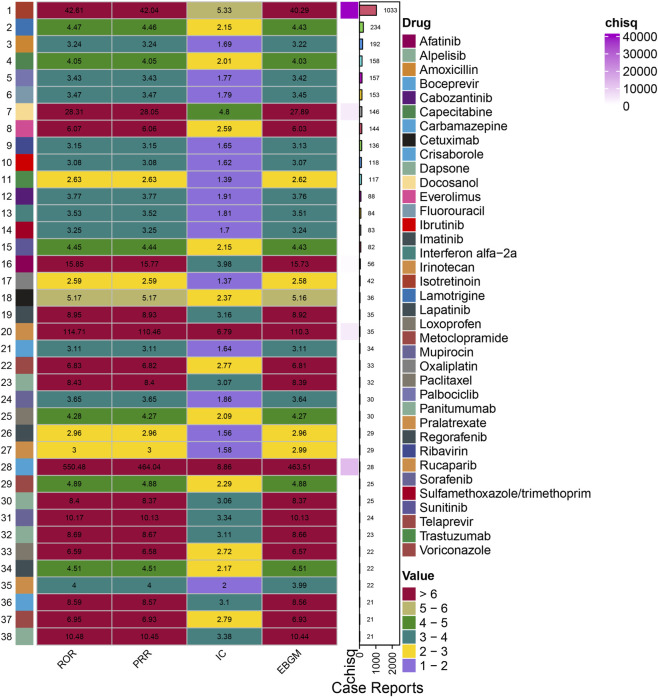
Relationships between drug profiles and cheilitis.

Disproportionality analysis identified 38 drugs with significant reporting associations with cheilitis (Figure 3). Isotretinoin was the most frequently reported medication, with 1,033 cases, and demonstrated the strongest association among high-volume drugs, as evidenced by a ROR of 42.61 (95% CI: 40.02–45.37), a PRR of 42.04 (95% CI: 39.64–44.59), and an Empirical Bayes Geometric Mean (EBGM) of 40.29 (EBGM05 = 38.23).

Notably, several topical and systemic agents used across diverse therapeutic areas showed strong signals. Docosanol, a topical antiviral, exhibited a high ROR of 28.31 (95% CI: 24.04–33.34). Among antineoplastic and immunomodulatory agents, drugs such as afatinib (ROR = 15.85, 95% CI: 12.18–20.61), everolimus (ROR = 6.07, 95% CI: 5.15–7.15), and cabozantinib (ROR = 3.77, 95% CI: 3.06–4.65) were prominently associated. The association for folotyn (pralatrexate) was particularly strong (ROR = 114.71, 95% CI: 81.82–160.83), albeit based on a smaller number of cases (n = 35). The strongest signal overall was observed for the topical phosphodiesterase-4 inhibitor crisaborole (ROR = 550.48, 95% CI: 367.64–824.26; n = 28).

Other significant associations were identified for antiepileptics (e.g., lamotrigine), antibiotics (e.g., amoxicillin, sulfamethoxazole/trimethoprim), and antiviral regimens (e.g., ribavirin, peginterferon alfa-2a). The consistency of signals across multiple disproportionality metrics (ROR, PRR, and EBGM) reinforces the robustness of these drug-cheilitis associations.

Supplementary Tables S3–S5 contain detailed analyses stratified by age, sex and weight.

Certain drugs, like crisaborole and pralatrexate, show very high disproportionality signals (ROR = 550.48 and ROR = 114.71, respectively) in the FAERS dataset, despite being based on fewer cases (28 and 35) compared to more commonly reported drugs like isotretinoin. These extreme values should be interpreted cautiously due to possible influences like increased clinical awareness, selective reporting, or statistical instability from small sample sizes. Nonetheless, these signals suggest potential high-risk agents that require further clinical attention and investigation.

### 3.3 Results of sensitivity analysis

To mitigate potential bias arising from incomplete demographic data and to assess the robustness of our primary findings, we conducted a sensitivity analysis limited to cases with comprehensive information on age, sex, and weight, as delineated in Supplementary Tables S3–S5. The outcomes of this complete-case analysis demonstrated a high degree of consistency with the primary disproportionality analysis, which encompassed all cases (Supplementary Table S6).

Isotretinoin consistently demonstrated the most robust and widespread signal across nearly all subgroups, with ROR values remaining exceptionally high (e.g., ROR = 53.26 in individuals under 19 years of age, 76.64 in males, and 104.16 in patients weighing over 100 kg), thereby reinforcing its well-established causal role. Similarly, docosanol and lamotrigine showed persistently strong associations across all age and sex categories after excluding cases with unknown data, confirming that the signals for these drugs are not merely artifacts of incomplete reporting.

It is important to note that certain extreme signals identified in the primary analysis, including those associated with crisaborole and pralatrexate, were derived from a limited number of cases and thus warrant cautious interpretation. The sensitivity analysis further emphasized that, although the disproportionality metrics for these signals remain elevated, the relatively low number of cases within specific demographic subgroups (e.g., individuals under 19 years of age) results in broader confidence intervals, reflecting statistical uncertainty. This highlights the necessity for ongoing post-marketing surveillance to validate these signals.

In summary, the substantial concordance between the primary and sensitivity analyses indicates that our principal findings are robust and not unduly influenced by records lacking demographic information. The consistent identification of high-risk signals for specific drug classes, namely, retinoids, EGFR inhibitors, and anticonvulsants, across all analytical scenarios enhances confidence in their biological plausibility and clinical significance.

### 3.4 Elements that influence drug-induced cheilitis

To identify independent risk factors for drug-induced cheilitis, we performed univariate and multivariate logistic regression analyses, adjusting for potential confounders. Several patient characteristics, comorbidities, and specific medications were significantly associated with an increased risk of cheilitis (Supplementary Tables S7, S8).

In the multivariate model, female sex was associated with a significantly higher risk of cheilitis (OR 0.771, 95% CI: 0.688–0.864; P < 0.001), whereas increasing age (OR 0.995 per unit increase, 95% CI: 0.993–0.997; P < 0.001) and higher body weight (OR 0.995 per unit increase, 95% CI: 0.993–0.997; P < 0.001) were associated with a modestly reduced risk. Among comorbidities, patients with diabetes mellitus (OR 0.285, 95% CI: 0.164–0.492; P < 0.001) or multiple myeloma (OR 0.448, 95% CI: 0.247–0.813; P = 0.008) had a significantly lower risk of developing cheilitis.

The most substantial risk factors identified were specific medications. Isotretinoin use was associated with a markedly elevated risk (OR 39.210, 95% CI: 31.749–48.424; P < 0.001). Strong associations were also observed for afatinib (OR 15.494, 95% CI: 5.775–41.568; P < 0.001), as well as for everolimus (OR 4.649, 95% CI: 1.495–14.459; P = 0.008), lamotrigine (OR 4.667, 95% CI: 1.743–12.493; P = 0.002), capecitabine (OR 3.949, 95% CI: 1.477–10.556; P = 0.006), and amoxicillin (OR 3.515, 95% CI: 1.573–7.852; P = 0.002).

These findings highlight distinct demographic and clinical profiles associated with drug-induced cheilitis and underscore several high-risk medications across therapeutic classes.

### 3.5 Network toxicology analysis

To systematically investigate the molecular mechanisms underlying drug-induced cheilitis, a network toxicology approach was employed. By integrating potential drug targets from the Comparative Toxicogenomics Database and disease-associated genes from GeneCards, a protein-protein interaction (PPI) network was constructed, followed by functional enrichment analysis.

**FIGURE 4:**
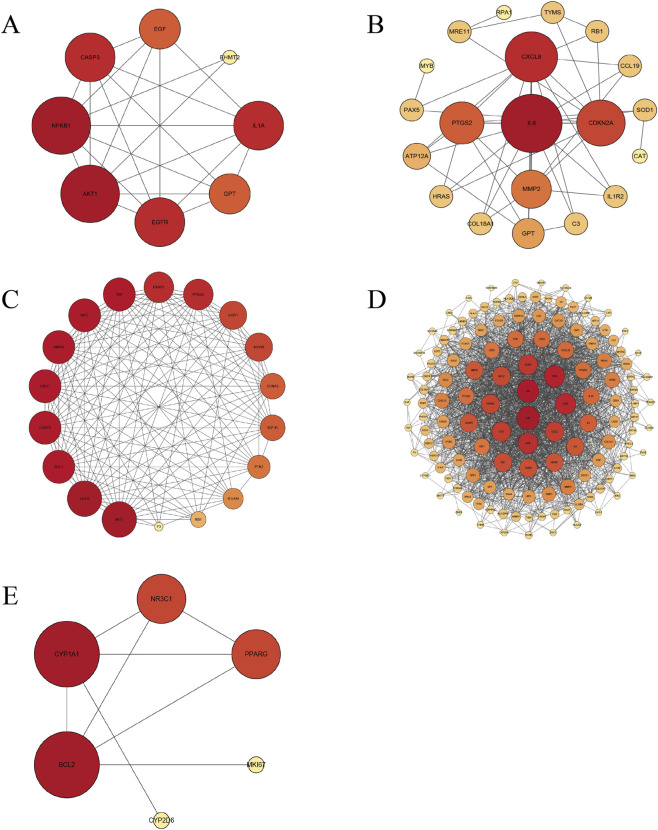
Protein-protein interaction (PPI) network analysis **(A)** Afatinb. **(B)** Capecitabine. **(C)** Everolimus. **(D)** Isotretinoin. **(E)** Lamotrigine.

The PPI network revealed several highly interconnected hub targets central to the pathogenesis of cheilitis (Figure 4). Key targets identified included IL6, TNF, AKT1, VEGFA, and STAT3, which exhibited high degree centrality, suggesting their pivotal roles in mediating inflammatory and immune responses. IL6, TNF, AKT1, VEGFA, and STAT3, known for their roles in systemic inflammation, are also enriched in cheilitis-related networks, indicating their coordinated involvement in lip-specific pathophysiology. VEGFA, crucial for angiogenesis, may aid drug delivery and immune cell recruitment due to the lip’s high vascular density. STAT3 and AKT1 are vital for epithelial growth and barrier repair, essential for the fast-regenerating lip mucosa.

**FIGURE 5:**
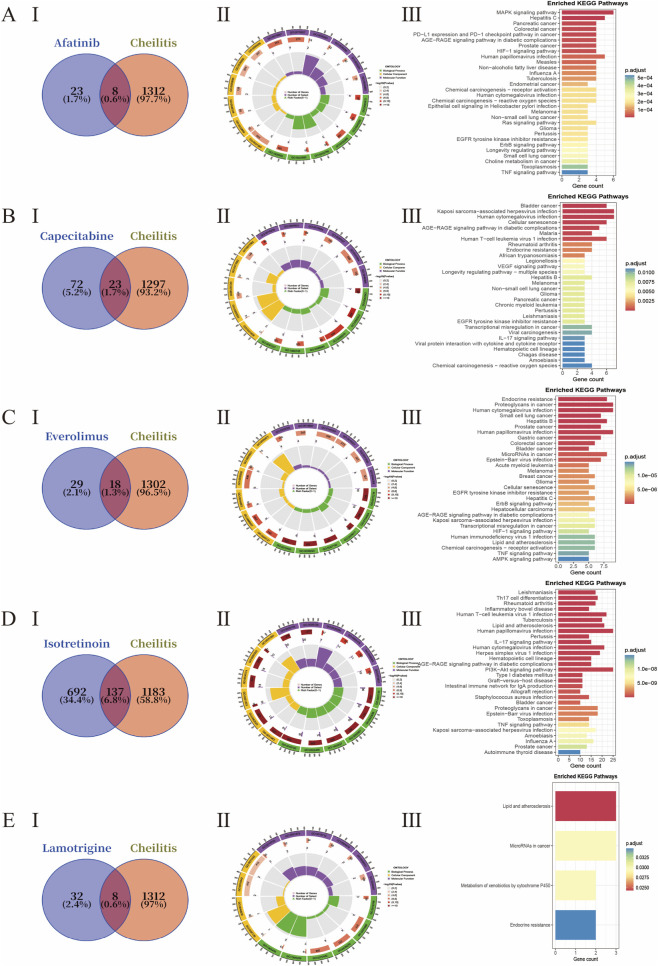
Network toxicology analysis unveils the core targets and pathways in drug-induced cheilitis. **(A)** Afatinb. **(B)** Capecitabine. **(C)** Everolimus. **(D)** Isotretinoin. **(E)** Lamotrigine. (I) Venn diagram. (II) Significantly enriched Gene Ontology (GO) terms for biological processes. (III) Significantly enriched Kyoto Encyclopedia of Genes and Genomes (KEGG) pathways.

Functional enrichment analysis of these hub targets revealed significant involvement in biological processes and signaling pathways relevant to cheilitis pathogenesis. Gene Ontology (GO) analysis highlighted enrichment in inflammatory responses (e.g., response to lipopolysaccharide, cytokine-mediated signaling pathway) and epidermal integrity (e.g., epithelial cell proliferation, skin development). Kyoto Encyclopedia of Genes and Genomes (KEGG) pathway analysis further identified several mechanistically pertinent pathways, including the IL-17 signaling pathway, TNF signaling pathway, and pathways related to various infections (e.g., Hepatitis B, Tuberculosis) which share common inflammatory cascades. Additionally, pathways in cancer, notably the PI3K-Akt signaling pathway, were enriched, reflecting the involvement of proliferative and survival mechanisms often triggered by high-risk medications such as kinase inhibitors (Figure 5).

These findings provide a systems-level perspective, positioning drug-induced cheilitis within a network of interconnected inflammatory, immune, and tissue remodeling pathways, and pinpointing core targets for subsequent mechanistic validation.

### 3.6 Molecular docking validates stable binding between high-risk drugs and core targets

To explore potential drug-target interactions predicted by network toxicology at an atomic level, molecular docking was performed for five high-risk drugs (afatinib, capecitabine, everolimus, isotretinoin, and lamotrigine) against their respective core protein targets (EGFR, IL-6, TNF-α, IL-6, and BCL-2) identified in the network analysis.

**FIGURE 6:**
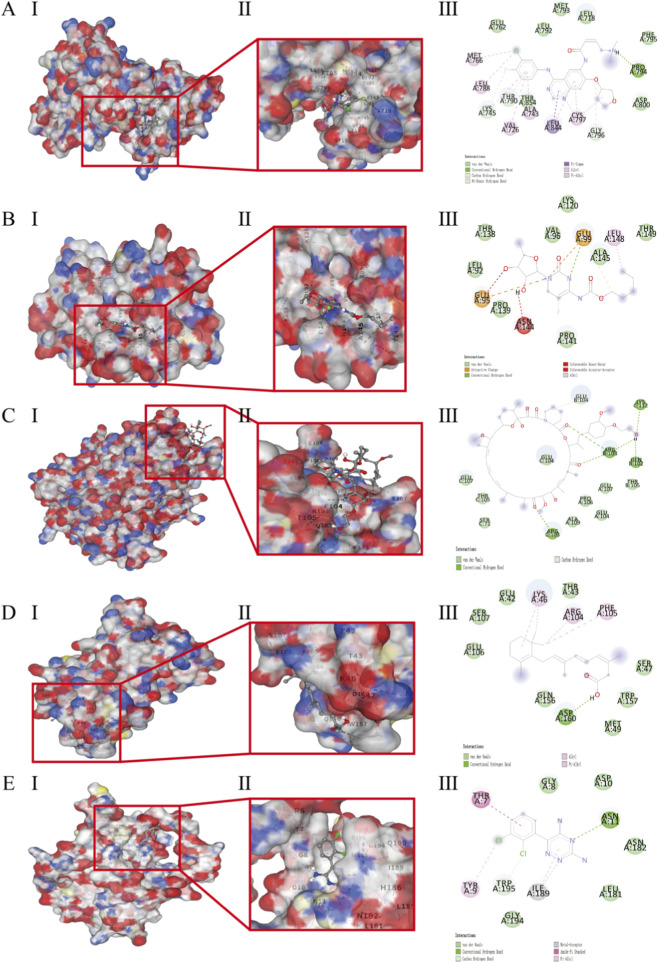
Molecular docking results of selected drugs against key targets. **(A)** Afatinb-EGFR. **(B)** Capecitabine-IL-6. **(C)** Everolimus-TNF-α. **(D)** Isotretinoin-IL-6. **(E)** Lamotrigine-BCL-2. (I) Protein-ligand binding site. (II) Protein–ligand complex structure. (III) Docked ligand structure.

All tested drug-target complexes exhibited favorable binding affinities, with calculated binding energies ranging from −8.1 to −6.2 kcal/mol (Table 2). Afatinib, an EGFR inhibitor, demonstrated the strongest binding energy of −8.1 kcal/mol with the EGFR kinase domain (PDB: 2EB2). Analysis of the binding pose revealed that afatinib formed critical hydrogen bonds with key residues, such as Met-793, and engaged in extensive hydrophobic interactions within the ATP-binding pocket, stabilizing the complex (Figure 6A).

**TABLE 2:** Molecular docking results of selected drugs against key targets, including binding energies (kcal/mol).

Drug	Compound CID	Target	PDBID	Binding energy (kcal/mol)
Afatinb	10184653	EGFR	2EB2	−8.1
Capecitabine	60953	IL-6	1ALU	−6.6
Everolimus	6442177	TNF-α	1A8M	−7.1
Isotretinoin	5282379	IL-6	1ALU	−6.2
Lamotrigine	3,878	BCL-2	1G5M	−6.6

Notably, both capecitabine and isotretinoin were docked into the same binding site of IL-6 (PDB: 1ALU), with binding energies of −6.6 kcal/mol and −6.2 kcal/mol, respectively. Their binding modes suggested potential interference with the IL-6 receptor binding interface, which could disrupt the pro-inflammatory signaling cascade (Figure 6B,D). Everolimus displayed a robust binding affinity of −7.1 kcal/mol for TNF-α (PDB: 1A8M), forming a stable complex that may impede TNF-α trimerization or receptor binding (Figure 6C). Lamotrigine, surprisingly, showed a significant binding propensity for the anti-apoptotic protein BCL-2 (PDB: 1G5M) with an energy of −6.6 kcal/mol, occupying its hydrophobic groove, which is crucial for protein-protein interactions in apoptosis regulation (Figure 6E). Detailed interaction profiles are summarized in Table 3 and Supplementary Table S9.

**TABLE 3:** Volume and three-dimensional structural parameters of the drug-protein complex.

Complex	Volume	Center x	Center y	Center z	Size x	Size y	Size z
Afatinb-EGFR	311	−0.312	53.607	23.378	25	25	25
Capecitabine-IL-6	722	5.783	−24.221	17.667	24	24	24
Everolimus-TNF-α	976	5.724	62.478	29.899	26	26	26
Isotretinoin-IL-6	166	14.041	−30.838	−0.15	23	23	23
Lamotrigine-BCL-2	285	−1.216	5.661	10.222	19	19	19

These molecular docking results provide atomistic evidence supporting the plausible direct interactions between high-risk drugs and central inflammatory or apoptotic targets, thereby bridging the gap between epidemiological associations and potential mechanistic underpinnings in drug-induced cheilitis.

The computational predictions indicate potential binding interactions; however, experimental validation, such as surface plasmon resonance or isothermal titration calorimetry, is required to substantiate these findings.

### 3.7 Molecular dynamics simulations

To assess the dynamic stability of the five drug-protein complex, 500 ns all-atom molecular dynamics simulations were performed.

**FIGURE 7:**
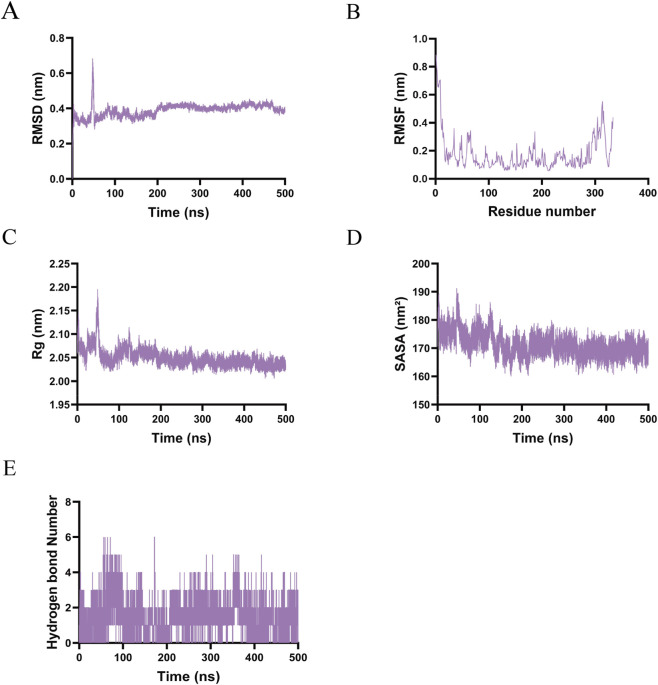
Molecular dynamics simulations results of Afatinb-EGFR. **(A)** RMSD. **(B)** RMSF. **(C)** Rg. **(D)** SASA. **(E)** The number of hydrogen bonds.

In the EGFR–Afatinib complex, the backbone root-mean-square deviation (RMSD) exhibited a rapid increase to approximately 0.4 nm within the initial 50 ns, followed by a peak at approximately 0.7 nm around 70 ns, before stabilizing within the range of 0.3–0.5 nm (Figure 7A). This pattern suggests an initial structural adjustment phase, succeeded by a stable equilibrium state. Root-mean-square fluctuation (RMSF) analysis indicated significant flexibility in the N-terminal (residues 1–50) and C-terminal (residues 300–400) regions, whereas the central kinase domain (residues 70–300) remained rigid, aligning with the characteristics of the binding core (Figure 7B). The radius of gyration (Rg) decreased from approximately 2.15 nm–2.08 nm during the first 50 ns and then stabilized between 2.03 and 2.10 nm, corroborating the occurrence of structural compaction (Figure 7C). The solvent-accessible surface area (SASA) decreased from approximately 190 nm^2^–175 nm^2^ initially and stabilized between 170 and 180 nm^2^, indicating the formation of a hydrophobic core (Figure 7D). The number of hydrogen bonds fluctuated between 0 and 6, predominantly ranging from 2 to 4, with transient peaks exceeding 5, which suggests dynamic binding adjustments while maintaining overall structural integrity (Figure 7E).

**FIGURE 8:**
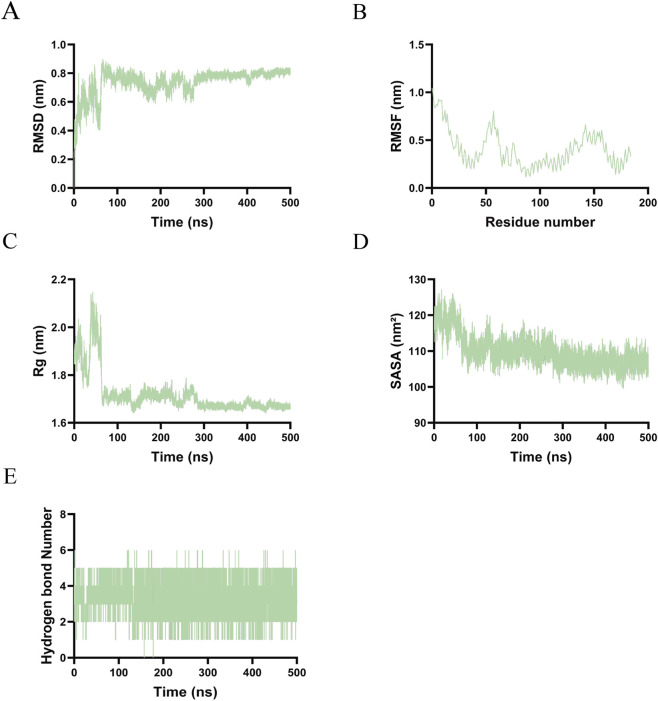
Molecular dynamics simulations results of Capecitabine-IL-6. **(A)** RMSD. **(B)** RMSF. **(C)** Rg. **(D)** SASA. **(E)** The number of hydrogen bonds.

In the IL6–Capecitabine complex, the backbone RMSD increased rapidly to approximately 0.8 nm within the first 100 ns and subsequently stabilized between 0.7 and 0.9 nm (Figure 8A). This behavior suggests an initial structural adjustment followed by the attainment of equilibrium. RMSF analysis indicated a high degree of flexibility in the N-terminal region (residues 1–50) and moderate flexibility in the C-terminal region (residues 150–200), whereas the central domain (residues 50–150) exhibited relative rigidity, consistent with its role as the binding core (Figure 8B). The Rg decreased from approximately 2.1 nm–1.7 nm during the initial 100 ns and remained stable between 1.6 and 1.7 nm, corroborating the occurrence of structural compaction (Figure 8C). The SASA decreased from approximately 125 nm^2^–110 nm^2^ initially and stabilized between 105 and 115 nm^2^, indicative of hydrophobic core formation (Figure 8D). The number of hydrogen bonds fluctuated between 0 and 6, predominantly ranging from 2 to 5, suggesting a dynamically stable network that preserved structural integrity (Figure 8E).

**FIGURE 9:**
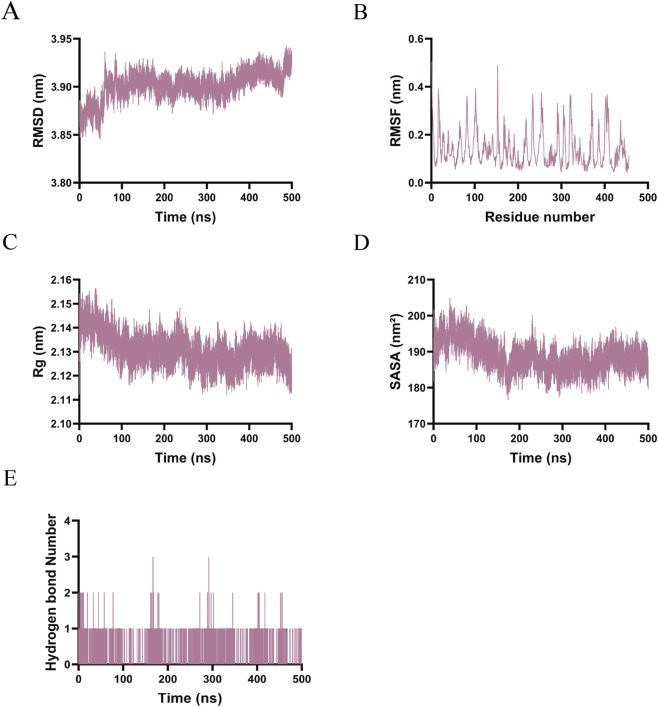
Molecular dynamics simulations results of Everolimus-TNF-α. **(A)** RMSD. **(B)** RMSF. **(C)** Rg. **(D)** SASA. **(E)** The number of hydrogen bonds.

Throughout the simulation, the backbone RMSD of the TNF-α–Everolimus complex ranged from 3.88 to 3.95 nm, indicating sustained conformational dynamics without achieving a fully rigid equilibrium state (Figure 9A). RMSF analysis demonstrated moderate to high flexibility across all residues (1–500), with frequent spikes reaching approximately 0.5 nm, suggesting considerable conformational plasticity (Figure 8B). The Rg remained stable between 2.11 and 2.15 nm, confirming the maintenance of overall compactness (Figure 9C). The SASA decreased from approximately 200 nm^2^ to about 185 nm^2^ initially, stabilizing between 180 and 195 nm^2^, which reflects the formation of a hydrophobic core (Figure 9D). The number of hydrogen bonds fluctuated between 0 and 3, predominantly between 0 and 1 (Figure 9E), with transient peaks indicating intermittent binding adjustments within a primarily hydrophobic interaction network.

**FIGURE 10:**
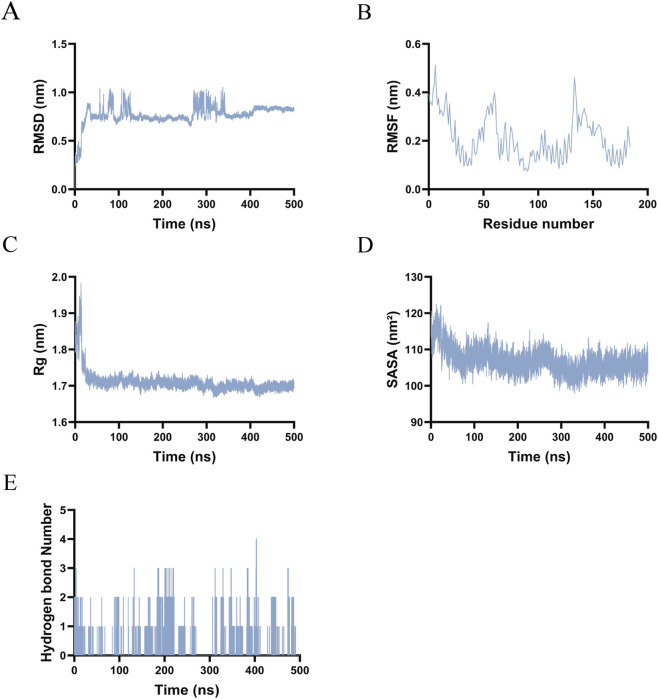
Molecular dynamics simulations results of Isotretinoin-IL-6. **(A)** RMSD. **(B)** RMSF. **(C)** Rg. **(D)** SASA. **(E)** The number of hydrogen bonds.

In the IL6–Isotretinoin complex, the backbone RMSD increased rapidly to approximately 0.8 nm within the first 50 nanoseconds (ns), exhibited fluctuations with peaks around 1.0 nm until 300 ns, and subsequently stabilized between 0.7 and 0.9 nm (Figure 10A). This pattern suggests an initial phase of structural adjustment followed by the attainment of equilibrium. RMSF analysis indicated high flexibility in the N-terminal region (residues 1–50) and moderate flexibility in the C-terminal region (residues 150–200), whereas the central domain (residues 50–150) exhibited relative rigidity, consistent with its role as the binding core (Figure 10B). The Rg decreased from approximately 2.0 nm–1.7 nm during the initial 50 ns and remained stable between 1.7 and 1.8 nm, corroborating the occurrence of structural compaction (Figure 10C). The SASA decreased from approximately 120 nm^2^–105 nm^2^ initially and stabilized within the range of 105–115 nm^2^, indicative of hydrophobic core formation (Figure 10D). The number of hydrogen bonds fluctuated between 0 and 4, predominantly ranging from 1 to 3, with transient peaks suggesting dynamic binding adjustments while maintaining overall structural integrity (Figure 10E).

**FIGURE 11:**
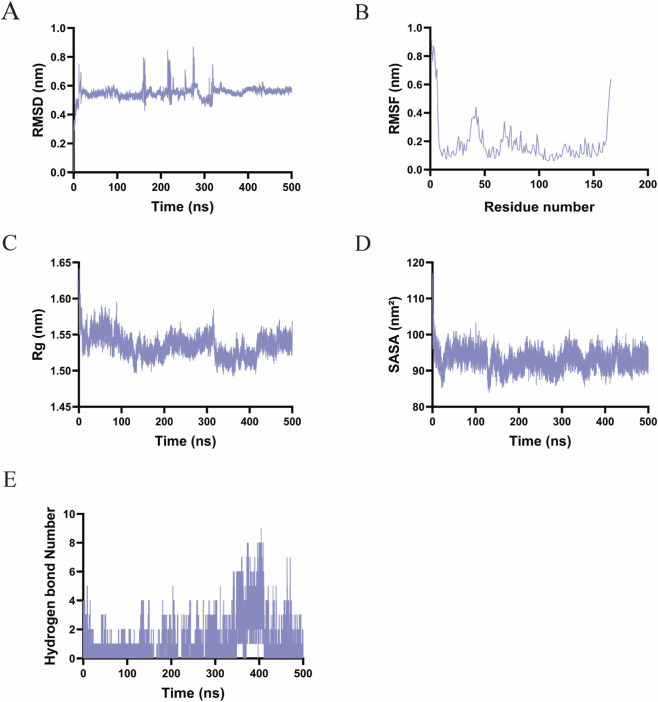
Molecular dynamics simulations results of Lamotrigine-BCL-2. **(A)** RMSD. **(B)** RMSF. **(C)** Rg. **(D)** SASA. **(E)** The number of hydrogen bonds.

In the BCL-2–Lamotrigine complex, the backbone RMSD increased rapidly to approximately 0.6 nm within the first 100 ns, exhibited fluctuations with peaks reaching around 1.0 nm until 300 ns, and subsequently stabilized at a range of 0.5–0.6 nm (Figure 11A). This behavior suggests an initial conformational rearrangement followed by the attainment of a stable equilibrium. RMSF analysis indicated significant flexibility in the N-terminal (residues 1–20) and C-terminal (residues 150–200) regions, whereas the central domain (residues 20–150) remained rigid, consistent with its role as a binding core (Figure 11B). The Rg decreased from 1.65 nm to approximately 1.55 nm within the first 50 ns and remained stable between 1.50 and 1.58 nm, corroborating the occurrence of structural compaction (Figure 11C). The SASA decreased from approximately 120 nm^2^ to about 90 nm^2^ initially and stabilized within the range of 90–100 nm^2^, indicating the formation of a hydrophobic core (Figure 11D). The number of hydrogen bonds fluctuated between 0 and 10, predominantly ranging from 2 to 6, with peaks exceeding 8 observed between 300 and 400 ns (Figure 11E). This pattern reflects dynamic binding adjustments while preserving overall structural integrity.

**FIGURE 12:**
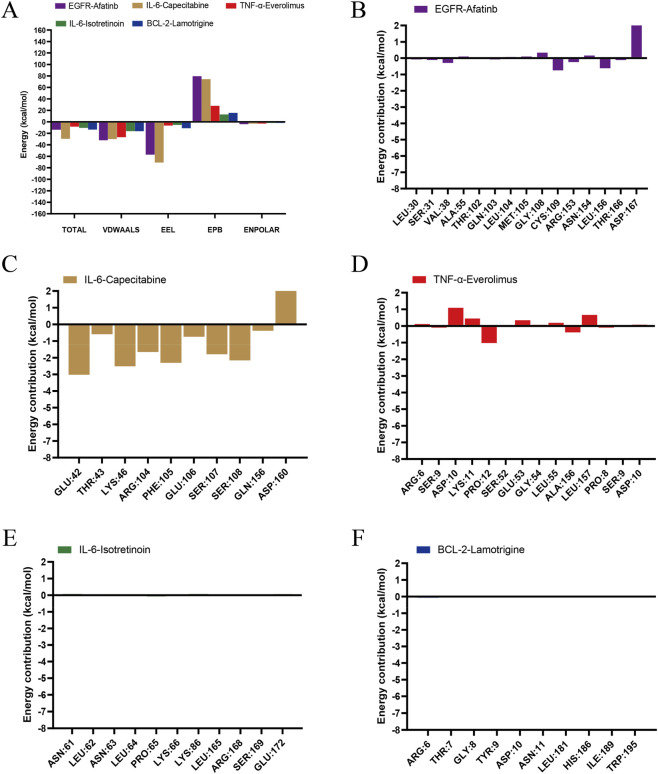
Study of energy partitioning and per-residue binding energy contributions in complexes of proteins and ligands. **(A)** Decomposing the binding energy of five different protein-ligand complexes. **(B)** Afatinb-EGFR. **(C)** Capecitabine-IL-6. **(D)** Everolimus-TNF-α. **(E)** Isotretinoin-IL-6. **(F)** Lamotrigine-BCL-2.

In order to assess the binding affinity and identify key residues of candidate drugs with their respective targets, we conducted molecular docking and energy decomposition analyses on five drug-target complexes (Figure 12A): EGFR-Afatinib (Figure 12B), IL-6-Capecitabine (Figure 12C), TNF-α-Everolimus (Figure 12D), IL-6-Isotretinoin (Figure 12E) and BCL-2-Lamotrigine (Figure 12F). The analysis of total binding energy indicated that the IL-6-Capecitabine and EGFR-Afatinib complexes formed stable interactions with their respective targets, primarily facilitated by robust van der Waals and electrostatic forces. In contrast, the TNF-α-Everolimus, IL-6-Isotretinoin, and BCL-2-Lamotrigine complexes exhibited negligible binding affinity, with total binding energies approximating zero. The residue energy contribution analysis highlighted ASP:167 as the critical stabilizing residue for the EGFR-Afatinib complex, whereas GLU:42, LYS:46, ARG:104, and PHE:105 were identified as key residues contributing to the stabilization of the IL-6-Capecitabine complex.

### 3.8 ADMET analysis

The ADMET (Absorption, Distribution, Metabolism, Excretion, and Toxicity) profiles of five candidate drugs—Afatinib, Capecitabine, Everolimus, Isotretinoin, and Lamotrigine—were evaluated to determine their druggability and safety (Table 4). Four of these compounds (Afatinib, Capecitabine, Isotretinoin, and Lamotrigine) complied with Lipinski’s rule of five, whereas Everolimus did not, due to its elevated molecular weight (957.58 Da) and number of hydrogen bond acceptors (nHA = 15 > 10). All the drugs exhibited negative LogS values, indicating poor aqueous solubility, and demonstrated low Caco-2 permeability, with a predicted oral bioavailability of only 30%. Notably, Lamotrigine showed excellent permeability across the blood-brain barrier (+++), while the other compounds displayed negligible penetration. Predicted plasma protein binding was high (>94%) for Afatinib, Everolimus, and Isotretinoin, in contrast to moderate-to-low binding observed for Capecitabine (62.1%) and Lamotrigine (48.0%). Everolimus was identified as a strong inhibitor of CYP3A4 (++), whereas Afatinib was a moderate inhibitor (+), with half-lives ranging from 0.711 h (Afatinib) to 1.954 h (Everolimus). Safety assessments projected a minimal risk of hERG channel blockade across all compounds. However, a significant risk of drug-induced liver injury (DILI) was predicted for Everolimus (1.000), Afatinib (0.998), Isotretinoin (0.984), and Capecitabine (0.927), whereas Lamotrigine demonstrated a low DILI risk (0.180). Additionally, Afatinib (0.865) and Everolimus (0.869) exhibited a high risk of genotoxicity, as indicated by the AMES test (++), with the remaining compounds presenting a moderate risk (+).

**TABLE 4:** ADMET analysis results of 5 drugs.

Drug	Molecular weight (MW)	LogS	F30%	Caco-2 permeability	BBB	PPB	CYP2D6 inhibitor	CYP3A4 inhibitor	T_1/2_	hERG blockers	DILI	AMES toxicity	Lipinski
Afatinb	485.16	−4.967	---	−5.253	---	95.3%	---	+	0.711	0.889	0.998	0.865	Yes
Capecitabine	359.15	−2.311	---	−5.380	---	62.1%	--	---	1.205	0.146	0.927	0.564	Yes
Everolimus	957.58	−4.384	+++	−5.426	---	94.7%	---	++	1.954	0.059	1.000	0.869	No
Isotretinoin	300.21	−3.905	---	−4.890	---	94.8%	--	---	0.952	0.076	0.644	0.565	Yes
Lamotrigine	255.01	−3.634	---	−4.584	+++	48.0%	---	---	1.183	0.180	0.984	0.582	Yes

The prediction probability values for classification endpoints are represented by six symbols: 0–0.1 (---), 0.1–0.3 (--), 0.3–0.5 (−), 0.5–0.7 (+), 0.7–0.9 (++), and 0.9–1.0 (+++).

## 4 Discussion

This research develops a comprehensive pharmacovigilance framework that integrates real-world clinical data with systems-level mechanistic analysis of drug-induced cheilitis. Through the triangulation of evidence from the FAERS database, network toxicology, and molecular dynamics simulations, we identified both well-known and previously under-recognized drug signals. Additionally, we elucidated potential biological pathways and formulated testable hypotheses concerning drug–target interactions.

The disproportionate reporting of isotretinoin, EGFR inhibitors (such as afatinib), and mTOR inhibitors (such as everolimus) is consistent with their established effects on epithelial proliferation and differentiation (Neill et al., 2023; Ornelas et al., 2016). Notably, the exceptionally strong signal associated with crisaborole—a topical agent—challenges the conventional assumption that cheilitis predominantly results from systemic drug exposure. This suggests that localized immune modulation or barrier disruption may also precipitate lip inflammation (Atarashi et al., 2009; Caron et al., 2025). This finding is clinically significant, as it underscores the necessity for increased vigilance even with topical therapies, particularly in patients with atopic dermatitis who may already have compromised skin barriers.

The identified hub targets are specific to the lip interactome, not just general inflammatory markers. The PI3K-Akt pathway is notably enriched, reflecting the lip epithelium’s metabolic and proliferative needs, making it a suitable target for kinase inhibitors such as afatinib. Additionally, the IL-17 pathway, significantly enriched, is linked to oral mucosal immunity and barrier defense, connecting drug exposure to localized lip inflammation.

From a clinical standpoint, our logistic regression analysis identified female sex as an independent risk factor, while increasing age and body weight appeared to confer modest protective effects. If these demographic patterns are validated in prospective cohorts, they could inform personalized monitoring strategies. For example, younger female patients commencing treatment with isotretinoin or afatinib might benefit from preemptive counseling and early dermatological follow-up. In contrast, the observed reduced risk in diabetic patients may be attributed to altered immune responsiveness or the effects of concomitant medications—an observation that necessitates further investigation rather than immediate clinical intervention.

It is essential to interpret the pharmacovigilance findings presented in this study as hypothesis-generating rather than as confirmation of causal risk. Spontaneous reporting systems, such as the FAERS, are inherently limited by issues such as under-reporting, reporting bias, and the lack of denominator data, which prevent accurate estimation of true incidence rates. Consequently, while signals like the notably high ROR of crisaborole (550.48) may indicate a potential safety concern, they do not establish causality. Such signals necessitate further validation through prospective observational studies or analyses of electronic health record cohorts.

Network toxicology and molecular docking analyses have provided mechanistic plausibility for the observed associations. The identification of IL6, TNF, and STAT3 as central targets, coupled with the enrichment of IL-17 and PI3K-Akt pathways, suggests a common inflammatory core potentially activated by various pharmacological agents. Furthermore, molecular docking and molecular dynamics simulations indicated stable interactions between high-risk drugs (such as afatinib with EGFR and capecitabine with IL-6) and these core targets, offering atomistic hypotheses regarding the direct modulation of inflammatory or apoptotic pathways in lip tissue by these drugs. Nonetheless, these computational predictions remain exploratory and necessitate experimental validation through techniques such as surface plasmon resonance, cellular thermal shift assays, or *in vivo* models.

This study is subject to several limitations. Firstly, the FAERS data are deficient in detailed clinical context, such as disease severity, concomitant therapies, and temporal plausibility, which may confound the associations between drugs and adverse events. Secondly, the network toxicology approach depends on publicly accessible databases that may contain incomplete or biased target annotations. Thirdly, although molecular docking and molecular dynamics simulations provide structural insights, they are unable to fully capture the complex *in vivo* pharmacokinetics or the immune microenvironment of the lip. Lastly, the lack of a replication cohort restricts the generalizability of the findings.

## 5 Conclusion

This study introduces the first multi-evidence framework for drug-induced cheilitis, linking population-level data with lip-specific mechanisms. By focusing on the unique anatomy and pathophysiology of the lip, we offer a model that surpasses general dermatotoxicity concepts. This approach improves post-marketing surveillance and supports targeted prevention strategies, like barrier creams for high-risk patients, as well as precision monitoring based on individual risk profiles.

## Data Availability

The original contributions presented in the study are included in the article/Supplementary Material, further inquiries can be directed to the corresponding author.
