# Supplementary material for: An integrative pharmacovigilance, network toxicology and molecular docking study on drug-induced cheilitis
Source: Front Pharmacol. 2026 Mar 20;17:1757807. doi: 10.3389/fphar.2026.1757807 (PMC13047072; doi:10.3389/fphar.2026.1757807)
Supplement: Supplementary file 2 [file Table8.docx]

**Table S8** Univariate and multivariate logistic regression.

|  | Univariate analysis  OR (95% CI) | P.value | Multivariate analysis  OR (95%CI) | P.value |
| --- | --- | --- | --- | --- |
| **Patient characteristics** |  |  |  |  |
| Age | 0.992 (0.990-0.994) | <0.001 |  |  |
| Sex | 0.811 (0.726-0.906) | <0.001 | 0.771 (0.688-0.864) | <0.001 |
| Wight | 0.992 (0.990-0.994) | <0.001 | 0.995 ( 0.993- 0.997) | <0.001 |
| **Indication** |  |  |  |  |
| Unknown indication | 0.598 (0.493-0.725) | <0.001 | 0.584 ( 0.481- 0.709) | <0.001 |
| Rheumatoid Arthritis | 0.896 (0.656-1.222) | 0.487 |  |  |
| Hypertension | 0.708 (0.521-0.963) | 0.028 |  |  |
| Myeloma | 0.430 (0.238-0.778) | 0.005 | 0.448 ( 0.247- 0.813) | 0.008 |
| Gastroesophageal reflux disease | 1.036 (0.572-1.875) | 0.908 |  |  |
| Diabetes mellitus | 0.264 (0.153-0.456) | <0.001 | 0.285 ( 0.164- 0.492) | <0.001 |
| Pain | 0.852 (0.596-1.216) | 0.377 |  |  |
| Depression | 0.962 (0.637-1.453) | 0.854 |  |  |
| Breast cancer | 1.198 (0.856-1.676) | 0.292 |  |  |
| Crohn’s disease | 1.065 (0.699-1.623) | 0.771 |  |  |
| **Drug** |  |  |  |  |
| Isotretinoin | 46.929 (38.640-56.996) | <0.001 | 39.210 (31.749-48.424) | <0.001 |
| Lamotrigine | 5.402 ( 2.022-14.433) | <0.001 | 4.667 ( 1.743-12.493) | 0.002 |
| Amoxicillin | 3.870 (1.734-8.636) | <0.001 | 3.515 ( 1.573- 7.852) | 0.002 |
| Capecitabine | 4.077 ( 1.527-10.889) | 0.005 | 3.949 ( 1.477-10.556) | 0.006 |
| Palbociclib | 2.155 (0.807-5.753) | 0.125 |  |  |
| Fluorouracil | 4.127 ( 0.580-29.374) | 0.157 |  |  |
| Everolimus | 4.900 ( 1.576-15.232) | 0.006 | 4.649 ( 1.495-14.459) | 0.008 |
| Ribavirin | 0.000 (0.000-inf) | 0.932 |  |  |
| Ibrutinib | 0.927 (0.130-6.589) | 0.940 |  |  |
| Trastuzumab | 0.000 (0.000-inf) | 0.906 |  |  |
| Cabozantinib | 0.000 (0.000-inf) | 0.905 |  |  |
| Interferon alfa-2a | 0.000 (0.000-inf) | 0.938 |  |  |
| Sulfamethoxazole trimethoprim | 4.845 ( 0.680-34.498) | 0.115 |  |  |
| Sunitinib | 1.905 (0.476-7.628) | 0.363 |  |  |
| Afatinib | 16.675 ( 6.225-44.667) | <0.001 | 15.494 ( 5.775-41.568) | <0.001 |
