# Supplementary material for: An integrative pharmacovigilance, network toxicology and molecular docking study on drug-induced cheilitis
Source: Front Pharmacol. 2026 Mar 20;17:1757807. doi: 10.3389/fphar.2026.1757807 (PMC13047072; doi:10.3389/fphar.2026.1757807)
Supplement: Supplementary file 3 [file Table7.docx]

**Table S7** Comparison of baseline characteristics and medication use in patients with and without cheilitis

| Variables | Total (n=2845918) | Non- cheilitis (n=2844555) | Cheilitis (n=1363) | Pvalue |
| --- | --- | --- | --- | --- |
| **Patient characteristics** |  |  |  |  |
| Age, median (Q1, Q3) | 59 (42, 70) | 59 (42, 70) | 56 (33, 68) | <0.001 |
| Sex, n (%) |  |  |  | <0.001 |
| Famale | 1708307 (60.03) | 1707422 (60.02) | 885 (64.93) |  |
| Male | 1137611 (39.97) | 1137133 (39.98) | 478 (35.07) |  |
| Weight, median (Q1, Q3) | 72.57 (59.86, 88) | 72.57 (59.86, 88) | 68 (56.7, 82) | <0.001 |
| **Indication** |  |  |  |  |
| Unknow indication, n (%) |  |  |  | < 0.001 |
| No | 2472182 (86.87) | 2470932 (86.87) | 1250 (91.71) |  |
| Yes | 373736 (13.13) | 373623 (13.13) | 113 (8.29) |  |
| Rheumatoid arthritis, n (%) |  |  |  | 0.535 |
| No | 2750667 (96.65) | 2749345 (96.65) | 1322 (96.99) |  |
| Yes | 95251 (3.35) | 95210 (3.35) | 41 (3.01) |  |
| Hypertension, n (%) |  |  |  | 0.032 |
| No | 2723627 (95.70) | 2722306 (95.70) | 1321 (96.92) |  |
| Yes | 122291 (4.30) | 122249 (4.30) | 42 (3.08) |  |
| Myeloma, n (%) |  |  |  | 0.006 |
| No | 2793109 (98.14) | 2791757 (98.14) | 1352 (99.19) |  |
| Yes | 52809 (1.86) | 52798 (1.86) | 11 (0.81) |  |
| Gastroesophageal reflux disease, n (%) |  |  |  | 1.000 |
| No | 2823737 (99.22) | 2822385 (99.22) | 1352 (99.19) |  |
| Yes | 22181 (0.78) | 22170 (0.78) | 11 (0.81) |  |
| Diabetes mellitus, n (%) |  |  |  | < 0.001 |
| No | 2745767 (96.48) | 2744417 (96.48) | 1350 (99.05) |  |
| Yes | 100151 (3.52) | 100138 (3.52) | 13 (0.95) |  |
| Pain, n (%) |  |  |  | 0.423 |
| No | 2770216 (97.34) | 2768884 (97.34) | 1332 (97.73) |  |
| Yes | 75702 (2.66) | 75671 (2.66) | 31 (2.27) |  |
| Depression, n (%) |  |  |  | 0.936 |
| No | 2796038 (98.25) | 2794698 (98.25) | 1340 (98.31) |  |
| Yes | 49880 (1.75) | 49857 (1.75) | 23 (1.69) |  |
| Breast cancer, n (%) |  |  |  | 0.336 |
| No | 2784648 (97.85) | 2783320 (97.85) | 1328 (97.43) |  |
| Yes | 61270 (2.15) | 61235 (2.15) | 35 (2.57) |  |
| Crohn, n (%) |  |  |  | 0.857 |
| No | 2802729 (98.48) | 2801388 (98.48) | 1341 (98.39) |  |
| Yes | 43189 (1.52) | 43167 (1.52) | 22 (1.61) |  |
| **Drug** |  |  |  |  |
| Isotretinoin, n (%) |  |  |  | < 0.001 |
| No | 2840336 (99.8) | 2839086 (99.81) | 1250 (91.71) |  |
| Yes | 5582 (0.2) | 5469 (0.19) | 113 (8.29) |  |
| Adalimumab, n (%) |  |  |  | 0.022 |
| No | 2819575 (99.07) | 2818216 (99.07) | 1359 (99.71) |  |
| Yes | 26343 (0.93) | 26339 (0.93) | 4 (0.29) |  |
| Dupilumab, n (%) |  |  |  | 0.469 |
| No | 2836564 (99.67) | 2835207 (99.67) | 1357 (99.56) |  |
| Yes | 9354 (0.33) | 9348 (0.33) | 6 (0.44) |  |
| Methotrexate, n (%) |  |  |  | 0.021 |
| No | 2842686 (99.89) | 2841328 (99.89) | 1358 (99.63) |  |
| Yes | 3232 (0.11) | 3227 (0.11) | 5 (0.37) |  |
| Lamotrigine, n (%) |  |  |  | 0.007 |
| No | 2844365 (99.95) | 2843006 (99.95) | 1359 (99.71) |  |
| Yes | 1553 (0.05) | 1549 (0.05) | 4 (0.29) |  |
| Palbociclib, n (%) |  |  |  | 0.118 |
| No | 2787850 (99.28) | 2785685 (99.28) | 1359 (99.71) |  |
| Yes | 20218 (0.72) | 20075 (0.72) | 4 (0.29) |  |
| Docosanol, n (%) |  |  |  | 0.009 |
| No | 2845900 (99.99) | 2844538 (99.99) | 1362 (99.93) |  |
| Yes | 18 (0.01) | 17 (0.01) | 1 (0.07) |  |
| Infliximab, n (%) |  |  |  | 0.324 |
| No | 2826012 (99.30) | 2824655 (99.30) | 1357 (99.56) |  |
| Yes | 19906 (0.7) | 19900 (0.7) | 6 (0.44) |  |
| Everolimus, n (%) |  |  |  | 0.025 |
| No | 2844635 (99.95) | 2843275 (99.96) | 1360 (99.78) |  |
| Yes | 1283 (0.05) | 1280 (0.04) | 3 (0.22) |  |
| Etanercept, n (%) |  |  |  | 0.121 |
| No | 2832527 (99.53) | 2831166 (99.53) | 1361 (99.85) |  |
| Yes | 13391 (0.47) | 13389 (0.47) | 2 (0.15) |  |
| Ibrutinib, n (%) |  |  |  | 1.000 |
| No | 2843666 (99.92) | 2842304 (99.92) | 1362 (99.93) |  |
| Yes | 2252 (0.08) | 2251 (0.08) | 1 (0.07) |  |
| Secukinumab, n (%) |  |  |  | 0.733 |
| No | 2841203 (99.83) | 2839841 (99.83) | 1362 (99.93) |  |
| Yes | 4715 (0.17) | 4714 (0.17) | 1 (0.07) |  |
| Aspirin, n (%) |  |  |  | 1.000 |
| No | 2845918 (100) | 2844555 (100) | 1363 (100) |  |
| Omalizumab, n (%) |  |  |  | 1.000 |
| No | 2843710 (99.92) | 2842348 (99.92) | 1362 (99.93) |  |
| Yes | 2208 (0.08) | 2207 (0.08) | 1 (0.07) |  |
| Prednisone, n (%) |  |  |  | 0.319 |
| No | 2845115 (99.97) | 2843753 (99.97) | 1362 (99.93) |  |
| Yes | 803 (0.03) | 802 (0.03) | 1 (0.07) |  |
| Trastuzumab, n (%) |  |  |  | 0.633 |
| No | 2843707 (99.92) | 2842344 (99.92) | 1363 (100) |  |
| Yes | 32211 (0.08) | 2211 (0.08) | 0 (0) |  |
| Capecitabine, n (%) |  |  |  | 0.018 |
| No | 2843862 (99.93) | 2842503 (99.93) | 1359 (99.71) |  |
| Yes | 2056 (0.07) | 2052 (0.07) | 4 (0.29) |  |
| Fluorouracil, n (%) |  |  |  | 0.216 |
| No | 2845411 (99.98) | 2844049 (99.98) | 1362 (99.93) |  |
| Yes | 507 (0.02) | 506 (0.02) | 1 (0.07) |  |
| Amlodipine, n (%) |  |  |  | 1.000 |
| No | 2845918 (100) | 2844555 (100) | 1363 (100) |  |
