# Supplementary material for: An integrative pharmacovigilance, network toxicology and molecular docking study on drug-induced cheilitis
Source: Front Pharmacol. 2026 Mar 20;17:1757807. doi: 10.3389/fphar.2026.1757807 (PMC13047072; doi:10.3389/fphar.2026.1757807)
Supplement: Supplementary file 4 [file Table9.docx]

**Table S9** The process of molecular docking explores the interaction between drug-protein.

| **Complex** | **van der Waals** | **Attractive Charge** | **Conventional Hydrogen Bond** | **Carbon Hydrogen Bond** | **Pi-Donor Hydrogen Bond** | **Pi-Sigma** | **Amide-Pi Stacked** | **Alkyl** | **Pi-Alkyl** | **Metal-Acceptor** | **Unfavorable Donor-Donor** | **Unfavorable Acceptor-Acceptor** |
| --- | --- | --- | --- | --- | --- | --- | --- | --- | --- | --- | --- | --- |
| Afatinb-EGFR | GLU-762 LEU-792 MET-793 LEU-718 PHE-795 ASP-800 THR-854 |  | PRO-794 | LYS-745 | THR-790 LYS-745 GLY-796 | LEU-844 |  | MET-766 LEU-788 VAL-726 ALA-743 CYS-797 | MET-766 LEU-788 VAL-726 ALA-743 CYS-797 |  |  |  |
| Capecitabine-IL-6 | LEU-92 THR-138 VAL-96 LYS-120 THR-149 PRO-139 PRO-141 | GLU-95 GLU-99 | GLU-99 | ALA-145 |  |  |  | LEU-148 |  |  | ASN-144 GLU-95 | ASN-144 |
| Everolimus-TNF-α | GLU-104 GLU-107 THR-105 SER-71 PRO-106 ALA-109 |  | ARG-103 LYS-122 GLN-102 | GLU-104 |  |  |  |  |  |  |  |  |
| Isotretinoin-IL-6 | GLU-42 THR-43 SER-107 GLU-106 SER-47 GLN-156 TRP-157 MET-49 |  | ASP-160 |  |  |  |  | LYS-46 ARG-104 | LYS-46 ARG-104 PHE-105 |  |  |  |
| Lamotrigine-BCL-2 | GLY-8 ASP-10 ASN-182 LEU-181GLY-194 |  | ASN-11 | TRP-195 |  |  | THR-7 TYR-9 |  | THR-7 TYR-9 ILE-189 | ILE-189 |  |  |
