# Supplementary material for: An integrative pharmacovigilance, network toxicology and molecular docking study on drug-induced cheilitis
Source: Front Pharmacol. 2026 Mar 20;17:1757807. doi: 10.3389/fphar.2026.1757807 (PMC13047072; doi:10.3389/fphar.2026.1757807)
Supplement: Supplementary file 5 [file Table2.docx]

**Table S2** This study utilizes ROR, PRR, and IC EBGM, in addition to the associated formulas and thresholds.

| Algorithms | Calculation formula | Criteria |
| --- | --- | --- |
| ROR | $\text{ROR}\text{=}\frac{\text{a}\text{/}\text{c}}{\text{b}\text{/}\text{d}}\text{=}\frac{\text{ad}}{\text{bc}}$  $\text{95\%}\text{CI}\text{=}\text{e}^{\text{In}\text{(}\text{ROR}\text{)±1.96}\sqrt{\frac{\text{1}}{\text{a}}\text{+}\frac{\text{1}}{\text{b}}\text{+}\frac{\text{1}}{\text{c}}\text{+}\frac{\text{1}}{\text{d}}}}$ | $\text{a}\text{≥3}$  $\text{ROR}\text{≥2}$  $\text{95\%}\text{CI}\text{>1}$ |
| PRR | $\text{PRR}\text{=}\frac{\text{a}\text{/(}\text{a}\text{+}\text{b}\text{)}}{\text{c}\text{/(}\text{c}\text{+}\text{d}\text{)}}\text{=}\frac{\text{a}\text{(}\text{c}\text{+}\text{d}\text{)}}{\text{c}\text{(}\text{a}\text{+}\text{b}\text{)}}$  $\text{χ}^{\text{2}}\text{=}\frac{\left( \left\vert\text{ad}\text{−}\text{bc} \right\vert\text{−}\frac{\text{n}}{\text{2}} \right)^{\text{2}}\text{n}}{\text{(}\text{a}\text{+}\text{b}\text{)(}\text{a}\text{+}\text{c}\text{)(}\text{c}\text{+}\text{d}\text{)(}\text{b}\text{+}\text{d}\text{)}}$  $\text{n}\text{=}\text{a}\text{+}\text{b}\text{+}\text{c}\text{+}\text{d}$ | $\text{a}\text{≥3}$  $\text{PRR}\text{≥2}$  $\text{χ}^{\text{2}}\text{≥4}$ |
| BCPNN | $\text{E}\left( \text{IC} \right)\text{=}\text{log}_{\text{2}}\frac{\left( \text{C}_{\text{xy}}\text{+}\text{γ}_{\text{11}} \right)\left( \text{C}\text{+}\text{α} \right)\left( \text{C}\text{+}\text{β} \right)}{\left( \text{C}\text{+}\text{γ} \right)\left( \text{C}_{\text{x}}\text{+}\text{α}_{\text{1}} \right)\left( \text{C}_{\text{y}}\text{+}\text{β}_{\text{1}} \right)}$  $\text{V}\left( \text{IC} \right)\text{=}\frac{\text{1}}{{\text{(}\text{In}\text{2)}}^{\text{2}}}\left\{ \left[ \frac{\text{C}\text{−}\text{C}_{\text{xy}}\text{+}\text{γ}\text{−}\text{γ}_{\text{11}}}{\left( \text{C}_{\text{xy}}\text{+}\text{γ}_{\text{11}} \right)\left( \text{1+}\text{C}\text{+}\text{γ} \right)} \right]\text{+}\left[ \frac{\text{C}\text{−}\text{C}_{\text{x}}\text{+}\text{α}\text{−}\text{α}_{\text{1}}}{\left( \text{C}_{\text{x}}\text{+}\text{α}_{\text{1}} \right)\left( \text{1+}\text{C}\text{+}\text{α} \right)} \right]\text{+}\left[ \frac{\text{C}\text{−}\text{C}_{\text{x}}\text{+}\text{α}\text{−}\text{α}_{\text{1}}}{\left( \text{C}_{\text{y}}\text{+}\text{β}_{\text{1}} \right)\left( \text{1+}\text{C}\text{+}\text{β} \right)} \right] \right\}$  $\text{γ}\text{=}\text{γ}_{\text{11}}\frac{\text{(}\text{C}\text{+}\text{α}\text{)(}\text{C}\text{+}\text{β}\text{)}}{\text{(}\text{C}_{\text{x}}\text{+}\text{α}_{\text{1}}\text{)(}\text{C}_{\text{y}}\text{+}\text{β}_{\text{1}}\text{)}}$  $\text{IC}\text{−2}\text{SD}\text{=}\text{E}\left( \text{IC} \right)\text{−2}\sqrt{\text{V}\text{(}\text{IC}\text{)}}$  $\text{α}_{\text{1}}\text{=}\text{β}_{\text{1}}\text{=1}$  $\text{α}\text{=}\text{β}\text{=2}$  $\text{γ}_{\text{11}}\text{=1}$  $\text{C}\text{=}\text{a}\text{+}\text{b}\text{+}\text{c}\text{+}\text{d}$  $\text{C}_{\text{x}}\text{=}\text{a}\text{+}\text{b}$  $\text{C}_{\text{y}}\text{=}\text{a}\text{+}\text{c}$  $\text{C}_{\text{xy}}\text{=}\text{a}$ | $\text{a}\text{≥3}$  $\text{IC}\text{−2}\text{SD}\text{>0}$ |
| EBGM | $\text{EBGM}\text{=}\text{a}\text{(}\text{a}\text{+}\text{b}\text{+}\text{c}\text{+}\text{d}\text{)/[(}\text{a}\text{+}\text{c}\text{)(}\text{a}\text{+}\text{b}\text{)]}$ | $\text{a}\text{>0}$  $\text{95\%}\text{CI}\text{>2}$ |
