# Supplementary material for: An integrative pharmacovigilance, network toxicology and molecular docking study on drug-induced cheilitis
Source: Front Pharmacol. 2026 Mar 20;17:1757807. doi: 10.3389/fphar.2026.1757807 (PMC13047072; doi:10.3389/fphar.2026.1757807)
Supplement: Supplementary file 6 [file Table5.docx]

**Table S5** Drug-induced cheilitis exhibits signaling differences based on weight.

| **Drug** | **Case Reports** | **ROR(95% CI)** | **PRR(95% CI)** | **IC(IC025)** | **EBGM(EBGM05)** | **weight** |
| --- | --- | --- | --- | --- | --- | --- |
| Isotretinoin | 27 | 21.27(14.5, 31.21) | 21(14.47, 30.48) | 4.37(3.82) | 20.61(14.96) | <50 |
| Lamotrigine | 14 | 3.27(1.93, 5.53) | 3.26(1.92, 5.53) | 1.7(0.96) | 3.24(2.08) | <50 |
| Capecitabine | 26 | 12.37(8.38, 18.26) | 12.28(8.3, 18.17) | 3.59(3.04) | 12.07(8.71) | <50 |
| Tykerb | 4 | 11.18(4.17, 29.93) | 11.1(4.17, 29.58) | 3.47(2.2) | 11.08(4.86) | <50 |
| Everolimus | 9 | 5.22(2.71, 10.07) | 5.21(2.73, 9.95) | 2.37(1.47) | 5.18(2.99) | <50 |
| Afatinib | 22 | 50.06(32.64, 76.78) | 48.56(32.18, 73.29) | 5.58(4.98) | 47.8(33.42) | <50 |
| Palbociclib | 11 | 8.51(4.69, 15.43) | 8.47(4.7, 15.25) | 3.07(2.25) | 8.41(5.11) | <50 |
| Fluorouracil | 14 | 3.49(2.06, 5.91) | 3.48(2.05, 5.91) | 1.79(1.05) | 3.46(2.22) | <50 |
| Zometa | 6 | 6.52(2.92, 14.56) | 6.5(2.91, 14.52) | 2.69(1.62) | 6.47(3.3) | <50 |
| Carbocisteine | 4 | 11.28(4.21, 30.2) | 11.2(4.2, 29.84) | 3.48(2.21) | 11.17(4.9) | <50 |
| Panitumumab | 3 | 9.05(2.91, 28.2) | 9.01(2.89, 28.08) | 3.17(1.75) | 8.99(3.47) | <50 |
| Tetracycline | 4 | 85.91(31.36, 235.38) | 81.5(31.19, 212.94) | 6.34(5.04) | 81.27(34.97) | <50 |
| Arikayce | 3 | 13.13(4.21, 40.95) | 13.03(4.18, 40.61) | 3.7(2.28) | 13(5.02) | <50 |
| Mobocertinib | 8 | 174.72(84, 363.41) | 157.34(80.8, 306.37) | 7.29(6.29) | 156.44(84.76) | <50 |
| Depas | 4 | 7.71(2.88, 20.63) | 7.68(2.88, 20.46) | 2.94(1.67) | 7.66(3.36) | <50 |
| Viramune | 3 | 6.67(2.14, 20.77) | 6.65(2.13, 20.73) | 2.73(1.31) | 6.64(2.57) | <50 |
| Carbamazepine | 3 | 6.54(2.1, 20.36) | 6.52(2.09, 20.32) | 2.7(1.28) | 6.51(2.52) | <50 |
| Trileptal | 4 | 4.68(1.75, 12.5) | 4.67(1.75, 12.44) | 2.22(0.95) | 4.66(2.05) | <50 |
| Phenobarbital tab | 4 | 16.42(6.12, 44.02) | 16.26(6.1, 43.32) | 4.02(2.74) | 16.21(7.1) | <50 |
| Betaseron | 3 | 22.27(7.12, 69.71) | 21.98(7.19, 67.18) | 4.45(3.03) | 21.93(8.44) | <50 |
| Kytril | 3 | 6.51(2.09, 20.25) | 6.49(2.08, 20.23) | 2.69(1.27) | 6.47(2.5) | <50 |
| Retrovir | 4 | 5.47(2.04, 14.61) | 5.45(2.05, 14.52) | 2.44(1.17) | 5.44(2.39) | <50 |
| Elidel | 5 | 29.16(12.02, 70.74) | 28.65(12.09, 67.87) | 4.84(3.67) | 28.55(13.6) | <50 |
| Erlotinib hydrochloride | 4 | 49.38(18.23, 133.79) | 47.9(18.33, 125.15) | 5.58(4.29) | 47.77(20.75) | <50 |
| Idursulfase | 7 | 5.91(2.81, 12.44) | 5.89(2.8, 12.4) | 2.55(1.55) | 5.87(3.15) | <50 |
| Oxaliplatin | 16 | 13.83(8.43, 22.69) | 13.72(8.41, 22.4) | 3.76(3.07) | 13.57(8.97) | <50 |
| Gemcitabine | 3 | 5.55(1.78, 17.26) | 5.53(1.77, 17.24) | 2.47(1.05) | 5.52(2.14) | <50 |
| Oxinorm | 3 | 9.81(3.15, 30.56) | 9.76(3.13, 30.42) | 3.28(1.86) | 9.74(3.76) | <50 |
| Fungizone | 3 | 14.11(4.52, 44.04) | 14(4.49, 43.63) | 3.8(2.38) | 13.97(5.39) | <50 |
| Simponi | 6 | 5.04(2.26, 11.26) | 5.03(2.25, 11.23) | 2.33(1.25) | 5.01(2.56) | <50 |
| Doxycycline. | 3 | 9.87(3.17, 30.76) | 9.82(3.15, 30.61) | 3.29(1.87) | 9.8(3.79) | <50 |
| Ispaghula extract | 5 | 713.21(247.47, 2055.42) | 490.64(237.58, 1013.24) | 8.93(7.59) | 488.87(201.63) | <50 |
| Amitriptyline hydrochloride | 5 | 69.42(28.3, 170.29) | 66.52(28.08, 157.58) | 6.05(4.87) | 66.29(31.29) | <50 |
| Bendroflumethiazide | 5 | 45.88(18.82, 111.81) | 44.6(18.83, 105.65) | 5.47(4.3) | 44.44(21.09) | <50 |
| Chlorphenamine | 5 | 28.52(11.76, 69.19) | 28.03(11.83, 66.4) | 4.8(3.63) | 27.94(13.31) | <50 |
| Diazepam | 5 | 5.58(2.32, 13.45) | 5.56(2.3, 13.43) | 2.47(1.31) | 5.55(2.66) | <50 |
| Enalapril | 5 | 5.78(2.4, 13.93) | 5.76(2.38, 13.91) | 2.52(1.36) | 5.74(2.75) | <50 |
| Hypromellose | 5 | 142.64(57.01, 356.86) | 130.84(56.33, 303.92) | 7.03(5.81) | 130.37(60.52) | <50 |
| Nystan | 4 | 570.15(181.32, 1792.76) | 418.38(180.11, 971.84) | 8.7(7.25) | 417.17(159.96) | <50 |
| Trametinib | 12 | 33.97(19.13, 60.32) | 33.27(18.85, 58.74) | 5.04(4.25) | 32.99(20.4) | <50 |
| Omalizumab | 15 | 7.71(4.63, 12.84) | 7.68(4.61, 12.78) | 2.93(2.21) | 7.6(4.96) | <50 |
| Imvexxy | 3 | 35.61(11.32, 111.96) | 34.84(11.4, 106.48) | 5.12(3.68) | 34.76(13.33) | <50 |
| Repatha | 3 | 16.84(5.39, 52.61) | 16.68(5.46, 50.98) | 4.06(2.63) | 16.64(6.42) | <50 |
| Sandostatin lar depot | 6 | 19.42(8.67, 43.52) | 19.2(8.6, 42.88) | 4.26(3.18) | 19.12(9.73) | <50 |
| Brigatinib | 6 | 84.11(36.93, 191.56) | 79.89(36.48, 174.98) | 6.31(5.21) | 79.55(39.95) | <50 |
| Zelitrex | 3 | 27.01(8.62, 84.67) | 26.57(8.69, 81.21) | 4.73(3.3) | 26.52(10.19) | <50 |
| Xaluprine | 3 | 235.02(69.76, 791.8) | 204.49(70.96, 589.29) | 7.67(6.14) | 204.05(73.85) | <50 |
| Ceftriaxone sodium | 14 | 75.46(44.02, 129.33) | 72.06(43.29, 119.95) | 6.16(5.41) | 71.34(45.45) | <50 |
| Flecainide | 3 | 17.6(5.63, 54.99) | 17.42(5.7, 53.24) | 4.12(2.69) | 17.38(6.7) | <50 |
| Ventavis | 3 | 14.97(4.8, 46.72) | 14.83(4.76, 46.22) | 3.89(2.46) | 14.8(5.71) | <50 |
| Velaglucerase alfa | 13 | 93.68(53.4, 164.33) | 88.48(52.12, 150.2) | 6.45(5.67) | 87.66(54.77) | <50 |
| Ofev | 5 | 11.71(4.85, 28.26) | 11.63(4.81, 28.09) | 3.53(2.37) | 11.59(5.54) | <50 |
| Isotretinoin | 571 | 67.89(62.29, 74) | 65.84(60.88, 71.21) | 5.94(5.82) | 61.46(57.18) | 50~100 |
| Lamotrigine | 62 | 4.21(3.28, 5.41) | 4.2(3.26, 5.42) | 2.06(1.71) | 4.18(3.39) | 50~100 |
| Peginterferon alfa-2a | 42 | 4.71(3.47, 6.37) | 4.7(3.5, 6.31) | 2.23(1.79) | 4.68(3.63) | 50~100 |
| Ribavirin | 59 | 3.56(2.75, 4.59) | 3.55(2.75, 4.58) | 1.82(1.45) | 3.53(2.85) | 50~100 |
| Tazocilline | 4 | 8.33(3.12, 22.25) | 8.3(3.12, 22.11) | 3.05(1.78) | 8.3(3.65) | 50~100 |
| Sulfamethoxazole/trimethoprim | 40 | 3.08(2.26, 4.2) | 3.08(2.25, 4.21) | 1.62(1.17) | 3.07(2.36) | 50~100 |
| Nexavar | 13 | 4.62(2.68, 7.97) | 4.61(2.66, 7.98) | 2.2(1.45) | 4.61(2.92) | 50~100 |
| Adalat | 6 | 4.29(1.92, 9.55) | 4.28(1.92, 9.56) | 2.1(1.03) | 4.28(2.19) | 50~100 |
| Sunitinib malate | 48 | 6.6(4.97, 8.78) | 6.59(5.01, 8.67) | 2.71(2.31) | 6.55(5.17) | 50~100 |
| Everolimus | 48 | 6.54(4.92, 8.68) | 6.52(4.96, 8.58) | 2.7(2.29) | 6.49(5.11) | 50~100 |
| Tyverb | 3 | 38.49(12.28, 120.66) | 37.78(12.36, 115.47) | 5.24(3.81) | 37.77(14.52) | 50~100 |
| Afatinib | 17 | 15.58(9.66, 25.12) | 15.47(9.66, 24.76) | 3.95(3.28) | 15.44(10.35) | 50~100 |
| Primperan | 8 | 4.39(2.19, 8.79) | 4.38(2.21, 8.7) | 2.13(1.19) | 4.38(2.45) | 50~100 |
| Palbociclib | 70 | 4.92(3.89, 6.22) | 4.91(3.88, 6.21) | 2.29(1.95) | 4.88(4) | 50~100 |
| Rifater | 3 | 29.29(9.37, 91.58) | 28.88(9.45, 88.27) | 4.85(3.42) | 28.87(11.12) | 50~100 |
| Ortho tri-cyclen | 7 | 19.57(9.3, 41.22) | 19.39(9.21, 40.84) | 4.28(3.27) | 19.38(10.39) | 50~100 |
| Fluorouracil | 73 | 3.61(2.87, 4.55) | 3.61(2.85, 4.57) | 1.84(1.51) | 3.58(2.95) | 50~100 |
| Ketek | 3 | 7.95(2.56, 24.72) | 7.92(2.54, 24.68) | 2.99(1.57) | 7.92(3.07) | 50~100 |
| Corticosteroids | 6 | 9.22(4.13, 20.56) | 9.18(4.11, 20.5) | 3.2(2.13) | 9.17(4.69) | 50~100 |
| Radiation therapy | 5 | 7.16(2.98, 17.24) | 7.14(2.96, 17.25) | 2.84(1.68) | 7.14(3.42) | 50~100 |
| Monopril | 4 | 9.02(3.38, 24.08) | 8.98(3.37, 23.93) | 3.17(1.9) | 8.98(3.95) | 50~100 |
| Nystatin | 11 | 5.25(2.9, 9.49) | 5.24(2.91, 9.43) | 2.39(1.57) | 5.23(3.19) | 50~100 |
| Detrol la | 8 | 4.88(2.44, 9.77) | 4.87(2.45, 9.67) | 2.28(1.34) | 4.87(2.72) | 50~100 |
| Lac-b | 3 | 18.14(5.82, 56.54) | 17.98(5.88, 54.95) | 4.17(2.75) | 17.98(6.94) | 50~100 |
| Mavik | 3 | 8.35(2.69, 25.96) | 8.32(2.67, 25.93) | 3.06(1.64) | 8.32(3.22) | 50~100 |
| Zometa | 25 | 3.21(2.16, 4.75) | 3.2(2.16, 4.74) | 1.68(1.12) | 3.2(2.3) | 50~100 |
| Hydrea | 4 | 4.6(1.72, 12.28) | 4.59(1.72, 12.23) | 2.2(0.93) | 4.59(2.02) | 50~100 |
| Lendormin | 4 | 6.53(2.45, 17.43) | 6.51(2.44, 17.35) | 2.7(1.43) | 6.51(2.86) | 50~100 |
| Fluconazole | 15 | 3.59(2.16, 5.96) | 3.59(2.16, 5.98) | 1.84(1.13) | 3.58(2.34) | 50~100 |
| Glyburide | 12 | 4.13(2.34, 7.28) | 4.12(2.33, 7.27) | 2.04(1.26) | 4.12(2.56) | 50~100 |
| Minomycin | 5 | 34.16(14.11, 82.71) | 33.6(14.18, 79.59) | 5.07(3.9) | 33.58(16.03) | 50~100 |
| Biofermin | 3 | 9.34(3, 29.03) | 9.3(2.98, 28.99) | 3.22(1.8) | 9.3(3.6) | 50~100 |
| Minocycline hcl | 4 | 7.83(2.93, 20.92) | 7.81(2.93, 20.81) | 2.96(1.7) | 7.8(3.43) | 50~100 |
| Izilox | 5 | 108.79(44.22, 267.67) | 103.24(43.58, 244.56) | 6.69(5.5) | 103.18(48.58) | 50~100 |
| Loxonin | 11 | 5.59(3.09, 10.11) | 5.58(3.1, 10.05) | 2.48(1.66) | 5.58(3.4) | 50~100 |
| Duphalac | 9 | 14.62(7.58, 28.17) | 14.52(7.6, 27.72) | 3.86(2.96) | 14.5(8.38) | 50~100 |
| Gasmotin | 3 | 5.3(1.71, 16.47) | 5.29(1.7, 16.49) | 2.4(0.99) | 5.29(2.05) | 50~100 |
| Vitamedin | 3 | 16.31(5.24, 50.83) | 16.19(5.19, 50.46) | 4.02(2.59) | 16.19(6.25) | 50~100 |
| Foraseq | 4 | 5.84(2.19, 15.6) | 5.83(2.19, 15.53) | 2.54(1.28) | 5.83(2.56) | 50~100 |
| Flagyl | 9 | 3.55(1.84, 6.82) | 3.54(1.85, 6.76) | 1.82(0.93) | 3.54(2.05) | 50~100 |
| Elplat | 10 | 9.6(5.16, 17.88) | 9.56(5.11, 17.9) | 3.26(2.4) | 9.55(5.68) | 50~100 |
| Irinotecan hydrochloride | 6 | 7.4(3.32, 16.49) | 7.37(3.3, 16.46) | 2.88(1.81) | 7.37(3.77) | 50~100 |
| Mucosta | 7 | 4.66(2.22, 9.79) | 4.65(2.21, 9.79) | 2.22(1.22) | 4.65(2.5) | 50~100 |
| Sorafenib | 5 | 4.63(1.93, 11.14) | 4.62(1.91, 11.16) | 2.21(1.05) | 4.62(2.22) | 50~100 |
| Carbocisteine | 4 | 5.42(2.03, 14.47) | 5.41(2.03, 14.41) | 2.44(1.17) | 5.41(2.38) | 50~100 |
| Oracilline | 6 | 28(12.51, 62.69) | 27.63(12.37, 61.71) | 4.79(3.71) | 27.61(14.07) | 50~100 |
| Rovamycine | 4 | 34.36(12.79, 92.35) | 33.8(12.69, 90.06) | 5.08(3.8) | 33.78(14.77) | 50~100 |
| Nauzelin | 3 | 7.94(2.55, 24.68) | 7.91(2.54, 24.65) | 2.98(1.57) | 7.91(3.06) | 50~100 |
| Panitumumab | 19 | 14.48(9.22, 22.75) | 14.38(9.16, 22.57) | 3.84(3.21) | 14.35(9.83) | 50~100 |
| Hirudoid | 3 | 6.2(1.99, 19.25) | 6.18(1.98, 19.26) | 2.63(1.21) | 6.18(2.39) | 50~100 |
| Minocin | 3 | 18.19(5.84, 56.71) | 18.04(5.9, 55.14) | 4.17(2.75) | 18.03(6.96) | 50~100 |
| Minocycline | 10 | 8.15(4.38, 15.17) | 8.12(4.34, 15.2) | 3.02(2.16) | 8.11(4.82) | 50~100 |
| Aredia | 10 | 13.09(7.03, 24.39) | 13.01(6.95, 24.36) | 3.7(2.84) | 13(7.72) | 50~100 |
| Amoban | 3 | 10.72(3.45, 33.35) | 10.67(3.42, 33.26) | 3.41(2) | 10.67(4.13) | 50~100 |
| Pydoxal | 3 | 9.94(3.2, 30.91) | 9.9(3.18, 30.86) | 3.31(1.89) | 9.89(3.83) | 50~100 |
| Incivek | 13 | 4.66(2.7, 8.03) | 4.65(2.69, 8.05) | 2.22(1.46) | 4.65(2.95) | 50~100 |
| Tetracycline | 5 | 11.94(4.96, 28.78) | 11.88(4.92, 28.7) | 3.57(2.41) | 11.87(5.69) | 50~100 |
| Chapstick classic | 3 | 1501(335.88, 6707.71) | 858.14(362.26, 2032.8) | 9.74(8.01) | 857.84(245.11) | 50~100 |
| Thyradin | 3 | 14.03(4.51, 43.68) | 13.94(4.47, 43.45) | 3.8(2.38) | 13.93(5.39) | 50~100 |
| Ortho tri cyclen | 3 | 9.15(2.94, 28.46) | 9.11(2.92, 28.39) | 3.19(1.77) | 9.11(3.53) | 50~100 |
| Rhinocort aqua | 3 | 20.35(6.53, 63.48) | 20.16(6.6, 61.61) | 4.33(2.91) | 20.15(7.78) | 50~100 |
| Levothyroxin | 6 | 5(2.24, 11.15) | 4.99(2.23, 11.15) | 2.32(1.25) | 4.99(2.55) | 50~100 |
| Urso | 6 | 5.73(2.57, 12.77) | 5.72(2.56, 12.78) | 2.51(1.44) | 5.71(2.92) | 50~100 |
| Imiquimod | 4 | 52.33(19.39, 141.24) | 51.02(19.53, 133.3) | 5.67(4.39) | 51(22.22) | 50~100 |
| Fluticasone | 10 | 3.77(2.03, 7.01) | 3.77(2.01, 7.06) | 1.91(1.06) | 3.76(2.24) | 50~100 |
| Azelastine | 7 | 9.57(4.55, 20.12) | 9.53(4.53, 20.07) | 3.25(2.25) | 9.53(5.12) | 50~100 |
| Telaprevir | 4 | 8.87(3.32, 23.68) | 8.83(3.31, 23.53) | 3.14(1.87) | 8.83(3.88) | 50~100 |
| Toprol | 3 | 7.22(2.32, 22.45) | 7.2(2.31, 22.44) | 2.85(1.43) | 7.2(2.79) | 50~100 |
| Tiotropium | 5 | 4.76(1.98, 11.44) | 4.75(1.97, 11.47) | 2.25(1.09) | 4.75(2.28) | 50~100 |
| Zyclara | 3 | 37.06(11.83, 116.14) | 36.41(11.91, 111.28) | 5.19(3.75) | 36.39(13.99) | 50~100 |
| Ocuvite | 4 | 4.93(1.85, 13.16) | 4.92(1.85, 13.11) | 2.3(1.03) | 4.92(2.16) | 50~100 |
| Sovriad | 5 | 11.89(4.93, 28.64) | 11.82(4.89, 28.55) | 3.56(2.4) | 11.82(5.66) | 50~100 |
| Vectibix | 26 | 22.49(15.27, 33.12) | 22.25(15.03, 32.93) | 4.47(3.92) | 22.18(16.05) | 50~100 |
| Fluticasone propionate | 6 | 4.17(1.87, 9.28) | 4.16(1.86, 9.29) | 2.06(0.99) | 4.16(2.13) | 50~100 |
| Clobetasol | 11 | 4.78(2.65, 8.64) | 4.77(2.65, 8.59) | 2.25(1.44) | 4.77(2.91) | 50~100 |
| Minocycline hydrochloride | 5 | 48.35(19.91, 117.43) | 47.24(19.94, 111.9) | 5.56(4.39) | 47.21(22.47) | 50~100 |
| Armodafinil | 3 | 22.4(7.18, 69.92) | 22.17(7.25, 67.76) | 4.47(3.05) | 22.16(8.55) | 50~100 |
| Opdivo | 17 | 3.07(1.9, 4.94) | 3.06(1.91, 4.9) | 1.61(0.95) | 3.06(2.05) | 50~100 |
| Ultravist | 8 | 13.33(6.65, 26.72) | 13.25(6.67, 26.31) | 3.73(2.78) | 13.23(7.39) | 50~100 |
| Cravit | 5 | 6.91(2.87, 16.62) | 6.89(2.85, 16.64) | 2.78(1.63) | 6.88(3.3) | 50~100 |
| Tagrisso | 10 | 5.95(3.2, 11.08) | 5.94(3.17, 11.12) | 2.57(1.71) | 5.93(3.53) | 50~100 |
| Tri-sprintec | 5 | 27.57(11.4, 66.66) | 27.21(11.49, 64.46) | 4.77(3.6) | 27.2(12.99) | 50~100 |
| Dronabinol | 3 | 7.22(2.32, 22.45) | 7.2(2.31, 22.44) | 2.85(1.43) | 7.2(2.79) | 50~100 |
| L-lysine | 3 | 16.59(5.32, 51.68) | 16.46(5.28, 51.3) | 4.04(2.62) | 16.45(6.36) | 50~100 |
| Nicotine polacrilex | 5 | 4.97(2.07, 11.96) | 4.96(2.05, 11.98) | 2.31(1.15) | 4.96(2.38) | 50~100 |
| Peniramin | 7 | 48.33(22.83, 102.31) | 47.21(22.86, 97.5) | 5.56(4.55) | 47.18(25.19) | 50~100 |
| Zelboraf | 4 | 4.76(1.78, 12.7) | 4.75(1.78, 12.66) | 2.25(0.98) | 4.75(2.09) | 50~100 |
| Buprenorphine | 4 | 4.96(1.86, 13.23) | 4.95(1.86, 13.19) | 2.31(1.04) | 4.95(2.18) | 50~100 |
| Stromectol | 8 | 36(17.89, 72.46) | 35.38(17.82, 70.26) | 5.14(4.19) | 35.35(19.69) | 50~100 |
| Bipreterax | 4 | 31.03(11.56, 83.33) | 30.57(11.47, 81.45) | 4.93(3.66) | 30.56(13.37) | 50~100 |
| Pentacarinat | 4 | 16.61(6.21, 44.45) | 16.48(6.19, 43.91) | 4.04(2.77) | 16.47(7.23) | 50~100 |
| Ciprofloxacine | 9 | 54.95(28.33, 106.6) | 53.51(28.02, 102.17) | 5.74(4.83) | 53.46(30.71) | 50~100 |
| Azelastine | 4 | 8.22(3.08, 21.95) | 8.19(3.07, 21.82) | 3.03(1.77) | 8.19(3.6) | 50~100 |
| Betamethasone sodium phosphate | 3 | 16.82(5.4, 52.4) | 16.69(5.35, 52.02) | 4.06(2.64) | 16.68(6.44) | 50~100 |
| Valaciclovir | 8 | 5.26(2.63, 10.53) | 5.25(2.64, 10.43) | 2.39(1.45) | 5.24(2.93) | 50~100 |
| Thalidomide | 10 | 7.52(4.04, 14) | 7.5(4.01, 14.04) | 2.91(2.05) | 7.49(4.45) | 50~100 |
| Neofordex | 7 | 100.11(46.85, 213.94) | 95.39(46.19, 196.99) | 6.57(5.55) | 95.32(50.49) | 50~100 |
| Dalteparin sodium | 5 | 6.44(2.68, 15.51) | 6.43(2.66, 15.53) | 2.68(1.53) | 6.42(3.08) | 50~100 |
| Lormetazepam | 10 | 11.48(6.16, 21.38) | 11.42(6.1, 21.38) | 3.51(2.66) | 11.41(6.78) | 50~100 |
| Hydrochlorothiazide/valsartan | 8 | 12.97(6.47, 26) | 12.89(6.49, 25.6) | 3.69(2.74) | 12.88(7.2) | 50~100 |
| Flavoxate hydrochloride | 5 | 500.45(187.78, 1333.74) | 400.56(182.89, 877.31) | 8.65(7.36) | 400.32(176.28) | 50~100 |
| Ginkgo | 9 | 35.48(18.35, 68.61) | 34.88(18.27, 66.6) | 5.12(4.22) | 34.84(20.07) | 50~100 |
| Matricaria recutita | 4 | 889.59(273.9, 2889.28) | 616.18(270.52, 1403.52) | 9.27(7.79) | 615.88(229.84) | 50~100 |
| Novalgin | 4 | 5.12(1.92, 13.66) | 5.11(1.92, 13.62) | 2.35(1.09) | 5.11(2.25) | 50~100 |
| Meropenem anhydrous | 3 | 61.26(19.42, 193.29) | 59.47(19.46, 181.76) | 5.89(4.45) | 59.45(22.73) | 50~100 |
| Cubicin | 6 | 5.68(2.55, 12.65) | 5.66(2.53, 12.64) | 2.5(1.43) | 5.66(2.89) | 50~100 |
| Avamys | 3 | 7.47(2.4, 23.21) | 7.44(2.39, 23.19) | 2.9(1.48) | 7.44(2.88) | 50~100 |
| Fusidic acid | 3 | 9.51(3.06, 29.59) | 9.47(3.04, 29.52) | 3.24(1.82) | 9.47(3.67) | 50~100 |
| Rocephine | 5 | 24.65(10.2, 59.56) | 24.36(10.28, 57.7) | 4.61(3.44) | 24.35(11.64) | 50~100 |
| Sarilumab | 5 | 19.32(8.01, 46.63) | 19.15(7.93, 46.26) | 4.26(3.1) | 19.14(9.16) | 50~100 |
| Cannabis sativa flowering top | 3 | 25.12(8.04, 78.46) | 24.82(8.12, 75.86) | 4.63(3.21) | 24.81(9.57) | 50~100 |
| Rosuvas | 3 | 428.86(123.22, 1492.6) | 353.35(127.52, 979.12) | 8.46(6.9) | 353.23(124.41) | 50~100 |
| Ibaril | 3 | 3002(501.54, 17968.72) | 1201.4(581.75, 2481.06) | 10.23(8.4) | 1200.97(268.72) | 50~100 |
| Desoximetason | 3 | 1000.67(250.22, 4001.86) | 667.44(265.67, 1676.81) | 9.38(7.71) | 667.21(209.2) | 50~100 |
| Kredex | 4 | 55.22(20.44, 149.14) | 53.76(20.58, 140.46) | 5.75(4.46) | 53.73(23.4) | 50~100 |
| Oramorph | 9 | 18.45(9.57, 35.57) | 18.29(9.58, 34.92) | 4.19(3.29) | 18.27(10.55) | 50~100 |
| Insuline glargine | 3 | 90.97(28.6, 289.39) | 87.06(29.05, 260.92) | 6.44(4.99) | 87.03(33.05) | 50~100 |
| Acenocoumarol | 5 | 10.15(4.21, 24.45) | 10.1(4.18, 24.4) | 3.34(2.18) | 10.1(4.84) | 50~100 |
| Amphotericine b | 4 | 210.69(75.18, 590.46) | 190.72(74.44, 488.63) | 7.57(6.24) | 190.63(80.49) | 50~100 |
| Folotyn | 34 | 195.69(137.52, 278.46) | 178.4(130.38, 244.11) | 7.47(6.97) | 177.69(132.27) | 50~100 |
| Arikayce | 3 | 13.55(4.35, 42.19) | 13.47(4.32, 41.98) | 3.75(2.33) | 13.46(5.21) | 50~100 |
| Tretinoin | 10 | 24.02(12.87, 44.82) | 23.74(12.93, 43.59) | 4.57(3.71) | 23.72(14.07) | 50~100 |
| Mobocertinib | 3 | 14.16(4.55, 44.09) | 14.07(4.51, 43.85) | 3.81(2.39) | 14.06(5.44) | 50~100 |
| Ketoconazole | 8 | 8.44(4.21, 16.9) | 8.4(4.23, 16.68) | 3.07(2.12) | 8.4(4.7) | 50~100 |
| Hydrochlorothiazide/ramipril | 6 | 37.77(16.84, 84.74) | 37.09(16.93, 81.24) | 5.21(4.13) | 37.07(18.85) | 50~100 |
| Ebastine | 6 | 7.86(3.52, 17.52) | 7.83(3.51, 17.49) | 2.97(1.9) | 7.82(4) | 50~100 |
| Agiolax | 3 | 428.86(123.22, 1492.6) | 353.35(127.52, 979.12) | 8.46(6.9) | 353.23(124.41) | 50~100 |
| Betamethasone/calcipotriene | 7 | 117.78(54.94, 252.5) | 111.29(53.89, 229.83) | 6.8(5.77) | 111.2(58.75) | 50~100 |
| Terbinafine | 7 | 14.88(7.07, 31.3) | 14.78(7.02, 31.13) | 3.88(2.88) | 14.76(7.92) | 50~100 |
| Ethinyl estradiol/levonorgestrel | 7 | 23.88(11.33, 50.32) | 23.61(11.21, 49.72) | 4.56(3.55) | 23.59(12.64) | 50~100 |
| Ferrous fumarate | 7 | 4.88(2.32, 10.24) | 4.87(2.31, 10.26) | 2.28(1.28) | 4.87(2.61) | 50~100 |
| Etonogestrel | 6 | 42.3(18.84, 94.97) | 41.44(18.92, 90.76) | 5.37(4.29) | 41.41(21.05) | 50~100 |
| Semaglutide | 7 | 11.85(5.63, 24.91) | 11.78(5.59, 24.81) | 3.56(2.55) | 11.77(6.32) | 50~100 |
| Fluocinolone acetonide | 7 | 50.06(23.64, 106) | 48.86(23.66, 100.9) | 5.61(4.6) | 48.82(26.06) | 50~100 |
| Albendazole | 7 | 92.82(43.49, 198.1) | 88.75(42.98, 183.28) | 6.47(5.45) | 88.68(47.03) | 50~100 |
| Ruxience | 14 | 6.33(3.75, 10.71) | 6.32(3.72, 10.73) | 2.66(1.93) | 6.31(4.06) | 50~100 |
| Klor-con m | 4 | 103.98(38.05, 284.16) | 98.89(37.85, 258.37) | 6.63(5.32) | 98.85(42.62) | 50~100 |
| Estradiol hemihydrate | 3 | 84.56(26.63, 268.53) | 81.18(26.56, 248.11) | 6.34(4.89) | 81.15(30.86) | 50~100 |
| Azathioprin | 3 | 25.66(8.21, 80.15) | 25.35(8.29, 77.48) | 4.66(3.24) | 25.34(9.77) | 50~100 |
| Cyproterone | 6 | 78.51(34.72, 177.54) | 75.59(34.51, 165.56) | 6.24(5.15) | 75.53(38.16) | 50~100 |
| Siliq | 15 | 78.29(46.72, 131.19) | 75.38(46.18, 123.04) | 6.23(5.51) | 75.25(48.85) | 50~100 |
| Sacituzumab govitecan | 7 | 9.9(4.71, 20.81) | 9.85(4.68, 20.74) | 3.3(2.3) | 9.85(5.29) | 50~100 |
| Isotretinoin | 32 | 134.32(93.71, 192.54) | 130.95(92.02, 186.35) | 6.96(6.45) | 124.29(91.96) | >100 |
| Gleevec | 3 | 24.47(7.85, 76.29) | 24.35(7.81, 75.89) | 4.6(3.18) | 24.24(9.36) | >100 |
| Erbitux | 12 | 125.13(70.18, 223.11) | 122.11(69.17, 215.58) | 6.9(6.1) | 119.78(73.83) | >100 |
| Sunitinib malate | 5 | 11.24(4.66, 27.12) | 11.22(4.64, 27.1) | 3.48(2.32) | 11.13(5.33) | >100 |
| Stivarga | 3 | 76.05(24.25, 238.52) | 74.92(24.51, 228.98) | 6.22(4.79) | 74.56(28.65) | >100 |
| Irinotecan | 5 | 56.23(23.2, 136.23) | 55.61(23.02, 134.34) | 5.79(4.62) | 55.17(26.31) | >100 |
| Ibrutinib | 14 | 26.95(15.85, 45.85) | 26.81(15.79, 45.51) | 4.71(3.97) | 26.24(16.82) | >100 |
| Rubraca | 3 | 94.46(30.06, 296.87) | 92.71(30.33, 283.35) | 6.53(5.09) | 92.27(35.4) | >100 |
| Fluorouracil | 11 | 17.86(9.83, 32.45) | 17.8(9.89, 32.05) | 4.13(3.3) | 17.5(10.62) | >100 |
| Incivek | 3 | 13.1(4.21, 40.78) | 13.07(4.19, 40.74) | 3.7(2.28) | 13.01(5.03) | >100 |
| Ranitidine | 3 | 6.74(2.17, 20.97) | 6.73(2.16, 20.98) | 2.75(1.33) | 6.7(2.59) | >100 |
| Infliximab | 18 | 4.92(3.08, 7.87) | 4.92(3.07, 7.88) | 2.27(1.61) | 4.81(3.25) | >100 |
| Diclofenac | 3 | 6.08(1.95, 18.91) | 6.07(1.95, 18.92) | 2.6(1.18) | 6.05(2.34) | >100 |
| Mirapex | 5 | 22.15(9.17, 53.52) | 22.06(9.13, 53.29) | 4.45(3.29) | 21.89(10.47) | >100 |
| Cephalexin | 4 | 21.95(8.2, 58.8) | 21.86(8.2, 58.25) | 4.44(3.17) | 21.73(9.53) | >100 |
| Lupron depot | 4 | 20.12(7.51, 53.89) | 20.05(7.52, 53.42) | 4.32(3.04) | 19.92(8.74) | >100 |
| Alendronate sodium | 6 | 12.62(5.64, 28.23) | 12.59(5.64, 28.12) | 3.64(2.57) | 12.48(6.36) | >100 |
| Terazosin | 4 | 23.38(8.73, 62.63) | 23.27(8.73, 62) | 4.53(3.26) | 23.13(10.14) | >100 |
| Tamiflu | 3 | 36.98(11.84, 115.46) | 36.71(11.78, 114.42) | 5.19(3.77) | 36.54(14.09) | >100 |
| Tresiba | 4 | 15.81(5.91, 42.33) | 15.77(5.92, 42.02) | 3.97(2.7) | 15.67(6.88) | >100 |
| Onivyde | 3 | 400.85(123.27, 1303.48) | 370.86(123.75, 1111.45) | 8.53(7.04) | 369.08(137.6) | >100 |
| Pembrolizumab | 3 | 7.23(2.32, 22.49) | 7.22(2.32, 22.5) | 2.85(1.43) | 7.19(2.78) | >100 |
| Ipilimumab | 3 | 12.8(4.11, 39.86) | 12.77(4.1, 39.8) | 3.67(2.25) | 12.72(4.92) | >100 |
| Fruquintinib | 3 | 361.74(111.72, 1171.26) | 337.14(112.49, 1010.4) | 8.39(6.91) | 335.53(125.54) | >100 |
| Budesonide | 4 | 24.45(9.12, 65.5) | 24.33(9.13, 64.83) | 4.6(3.32) | 24.18(10.6) | >100 |
| Isotretinoin | 379 | 27.03(24.4, 29.95) | 26.84(24.33, 29.6) | 4.71(4.56) | 26.09(23.95) | unknow |
| Valtrex | 19 | 4(2.55, 6.27) | 3.99(2.54, 6.26) | 2(1.36) | 3.99(2.74) | unknow |
| Vfend | 20 | 11.65(7.51, 18.08) | 11.62(7.55, 17.88) | 3.54(2.92) | 11.6(8.03) | unknow |
| Amoxicillin | 124 | 4.22(3.53, 5.04) | 4.21(3.53, 5.02) | 2.07(1.81) | 4.18(3.61) | unknow |
| Depakene | 13 | 7.22(4.19, 12.45) | 7.21(4.16, 12.48) | 2.85(2.09) | 7.21(4.57) | unknow |
| Lamotrigine | 146 | 4.86(4.13, 5.72) | 4.85(4.15, 5.67) | 2.27(2.03) | 4.81(4.2) | unknow |
| Gleevec | 21 | 3.13(2.04, 4.81) | 3.13(2.03, 4.82) | 1.65(1.04) | 3.13(2.19) | unknow |
| Erbitux | 17 | 4.63(2.87, 7.45) | 4.62(2.89, 7.39) | 2.21(1.54) | 4.62(3.1) | unknow |
| Docosanol | 144 | 34.96(29.64, 41.23) | 34.64(29.61, 40.52) | 5.1(4.86) | 34.27(29.85) | unknow |
| Peginterferon alfa-2a | 39 | 2.91(2.12, 3.98) | 2.9(2.12, 3.97) | 1.54(1.09) | 2.9(2.23) | unknow |
| Ribavirin | 71 | 2.94(2.33, 3.72) | 2.94(2.32, 3.72) | 1.55(1.22) | 2.93(2.41) | unknow |
| Tegretol | 24 | 4.03(2.7, 6.02) | 4.03(2.72, 5.96) | 2.01(1.44) | 4.02(2.88) | unknow |
| Quinine | 12 | 13.3(7.54, 23.44) | 13.25(7.51, 23.39) | 3.73(2.94) | 13.24(8.24) | unknow |
| Rocephin | 12 | 8.79(4.99, 15.5) | 8.77(4.97, 15.48) | 3.13(2.35) | 8.77(5.46) | unknow |
| Tarceva | 24 | 2.88(1.93, 4.31) | 2.88(1.95, 4.26) | 1.53(0.96) | 2.88(2.06) | unknow |
| Capecitabine | 89 | 3.99(3.24, 4.92) | 3.99(3.22, 4.95) | 1.99(1.69) | 3.97(3.33) | unknow |
| Tazocilline | 10 | 13.65(7.34, 25.41) | 13.61(7.27, 25.48) | 3.77(2.91) | 13.6(8.08) | unknow |
| Sulfamethoxazole/trimethoprim | 37 | 3.36(2.44, 4.64) | 3.36(2.46, 4.6) | 1.75(1.29) | 3.35(2.56) | unknow |
| Acitretin | 10 | 15.97(8.58, 29.73) | 15.9(8.49, 29.77) | 3.99(3.14) | 15.89(9.45) | unknow |
| Nexavar | 16 | 3.35(2.05, 5.47) | 3.35(2.05, 5.47) | 1.74(1.06) | 3.35(2.22) | unknow |
| Clindamycin | 10 | 3.87(2.08, 7.19) | 3.86(2.06, 7.23) | 1.95(1.1) | 3.86(2.3) | unknow |
| Nicorette | 16 | 4.37(2.68, 7.14) | 4.37(2.68, 7.13) | 2.13(1.44) | 4.36(2.89) | unknow |
| Tykerb | 28 | 11.39(7.86, 16.52) | 11.36(7.83, 16.49) | 3.5(2.98) | 11.34(8.31) | unknow |
| Trastuzumab | 99 | 4.3(3.53, 5.24) | 4.3(3.53, 5.23) | 2.09(1.81) | 4.27(3.62) | unknow |
| Clarithromycin | 17 | 5.09(3.16, 8.19) | 5.08(3.17, 8.13) | 2.34(1.68) | 5.08(3.41) | unknow |
| Femara | 12 | 4.52(2.56, 7.96) | 4.51(2.55, 7.96) | 2.17(1.39) | 4.51(2.81) | unknow |
| Taxol | 28 | 9.7(6.69, 14.07) | 9.68(6.67, 14.05) | 3.27(2.75) | 9.66(7.08) | unknow |
| Adalat | 13 | 10.88(6.31, 18.76) | 10.85(6.27, 18.78) | 3.44(2.68) | 10.84(6.87) | unknow |
| Colchicine | 13 | 5.53(3.21, 9.53) | 5.52(3.19, 9.56) | 2.46(1.71) | 5.52(3.5) | unknow |
| Bevacizumab | 35 | 4.02(2.88, 5.6) | 4.01(2.87, 5.6) | 2(1.53) | 4.01(3.03) | unknow |
| Voriconazole | 28 | 10.23(7.06, 14.83) | 10.21(7.04, 14.82) | 3.35(2.82) | 10.19(7.47) | unknow |
| Sunitinib malate | 28 | 2.79(1.92, 4.04) | 2.79(1.92, 4.05) | 1.48(0.95) | 2.78(2.04) | unknow |
| Amikacin | 11 | 6.81(3.77, 12.3) | 6.8(3.78, 12.24) | 2.76(1.95) | 6.79(4.14) | unknow |
| Prednisolon | 10 | 7.85(4.22, 14.61) | 7.84(4.19, 14.68) | 2.97(2.12) | 7.83(4.66) | unknow |
| Everolimus | 87 | 6.19(5.02, 7.65) | 6.19(4.99, 7.68) | 2.62(2.32) | 6.15(5.16) | unknow |
| Mupirocin | 20 | 18.03(11.62, 27.99) | 17.95(11.66, 27.63) | 4.16(3.54) | 17.92(12.41) | unknow |
| Pegintron | 13 | 6.03(3.5, 10.39) | 6.02(3.48, 10.42) | 2.59(1.83) | 6.02(3.82) | unknow |
| Victrelis | 18 | 11.72(7.38, 18.62) | 11.68(7.3, 18.7) | 3.54(2.89) | 11.67(7.92) | unknow |
| Ursodiol | 11 | 6.41(3.55, 11.58) | 6.4(3.55, 11.52) | 2.68(1.86) | 6.39(3.9) | unknow |
| Stivarga | 16 | 4.94(3.03, 8.08) | 4.94(3.03, 8.06) | 2.3(1.62) | 4.93(3.27) | unknow |
| Tyverb | 10 | 74.29(39.71, 138.99) | 72.81(39.66, 133.68) | 6.19(5.32) | 72.76(43.08) | unknow |
| Vinorelbine tartrate | 15 | 62.8(37.68, 104.65) | 61.74(37.09, 102.77) | 5.95(5.23) | 61.67(40.22) | unknow |
| Lapatinib ditosylate | 10 | 750.67(379.72, 1484.01) | 621.42(351.99, 1097.08) | 9.28(8.35) | 620.94(351.07) | unknow |
| Abraxane | 16 | 4.72(2.89, 7.71) | 4.72(2.89, 7.7) | 2.24(1.55) | 4.71(3.13) | unknow |
| Afatinib | 17 | 8.25(5.12, 13.28) | 8.23(5.14, 13.17) | 3.04(2.37) | 8.22(5.52) | unknow |
| Cabozantinib s-malate | 70 | 5.04(3.98, 6.38) | 5.04(3.98, 6.38) | 2.33(1.99) | 5.01(4.12) | unknow |
| Valacyclovir | 15 | 4.25(2.56, 7.05) | 4.25(2.55, 7.07) | 2.08(1.38) | 4.24(2.78) | unknow |
| Primperan | 13 | 11.86(6.88, 20.46) | 11.83(6.83, 20.48) | 3.56(2.81) | 11.82(7.49) | unknow |
| Pantosin | 14 | 144.17(84.48, 246.04) | 138.67(83.3, 230.83) | 7.11(6.37) | 138.52(88.57) | unknow |
| Magnesium oxide | 14 | 573.42(326.16, 1008.11) | 494.85(303.16, 807.75) | 8.95(8.17) | 494.32(308.3) | unknow |
| Cetuximab | 18 | 10.98(6.91, 17.44) | 10.95(6.84, 17.53) | 3.45(2.8) | 10.93(7.42) | unknow |
| Carbamazepine | 13 | 3.5(2.03, 6.03) | 3.5(2.02, 6.06) | 1.81(1.05) | 3.5(2.22) | unknow |
| Mesalazine | 10 | 8.65(4.65, 16.09) | 8.63(4.61, 16.16) | 3.11(2.25) | 8.62(5.13) | unknow |
| Nystatin. | 14 | 6.87(4.07, 11.61) | 6.86(4.04, 11.65) | 2.78(2.05) | 6.85(4.42) | unknow |
| Irinotecan | 23 | 4.23(2.81, 6.37) | 4.22(2.8, 6.37) | 2.08(1.5) | 4.22(3) | unknow |
| Palbociclib | 66 | 2.49(1.95, 3.17) | 2.49(1.97, 3.15) | 1.31(0.96) | 2.48(2.02) | unknow |
| Ibrutinib | 88 | 3.47(2.81, 4.28) | 3.47(2.8, 4.3) | 1.79(1.49) | 3.45(2.89) | unknow |
| Rubraca | 19 | 4.77(3.04, 7.48) | 4.76(3.03, 7.47) | 2.25(1.62) | 4.76(3.26) | unknow |
| Septrin | 13 | 41.32(23.91, 71.39) | 40.86(23.6, 70.74) | 5.35(4.59) | 40.82(25.83) | unknow |
| Clemastin | 12 | 270.28(150.29, 486.07) | 251.5(145.28, 435.39) | 7.97(7.16) | 251.27(153.77) | unknow |
| Piqray | 24 | 11.39(7.62, 17) | 11.35(7.67, 16.8) | 3.5(2.94) | 11.33(8.1) | unknow |
| Niraparib | 17 | 3.81(2.36, 6.13) | 3.8(2.37, 6.08) | 1.93(1.26) | 3.8(2.55) | unknow |
| Voriconazole | 12 | 4.34(2.46, 7.64) | 4.33(2.45, 7.64) | 2.11(1.33) | 4.33(2.7) | unknow |
| Tukysa | 10 | 7.38(3.97, 13.74) | 7.37(3.94, 13.8) | 2.88(2.03) | 7.37(4.38) | unknow |
| Kynmobi | 16 | 18.18(11.12, 29.72) | 18.1(11.09, 29.54) | 4.18(3.49) | 18.08(11.98) | unknow |
| Crisaborole | 22 | 721.31(456.22, 1140.41) | 601.26(414.32, 872.56) | 9.23(8.59) | 600.25(409.14) | unknow |
| Dapsone | 17 | 16.02(9.94, 25.8) | 15.95(9.96, 25.53) | 3.99(3.33) | 15.93(10.69) | unknow |
| Hydrocortisone valerate | 14 | 239.15(139.2, 410.88) | 224.33(134.76, 373.43) | 7.81(7.05) | 224.09(142.48) | unknow |
| Valacyclovir hydrochloride | 15 | 7.86(4.73, 13.05) | 7.84(4.71, 13.05) | 2.97(2.26) | 7.84(5.13) | unknow |
| Paraffin | 11 | 114.23(62.66, 208.25) | 110.75(61.51, 199.39) | 6.79(5.96) | 110.66(66.95) | unknow |
