# Supplementary material for: An integrative pharmacovigilance, network toxicology and molecular docking study on drug-induced cheilitis
Source: Front Pharmacol. 2026 Mar 20;17:1757807. doi: 10.3389/fphar.2026.1757807 (PMC13047072; doi:10.3389/fphar.2026.1757807)
Supplement: Supplementary file 7 [file Table3.docx]

**Table S3** Drug-induced cheilitis exhibits signaling differences based on age.

| **Drug** | **Case Reports** | **ROR (95% CI)** | **PRR (95% CI)** | **IC (IC025)** | **EBGM (EBGM05)** | **Sex** |
| --- | --- | --- | --- | --- | --- | --- |
| Lamotrigine | 30 | 3.28(2.28, 4.71) | 3.27(2.3, 4.65) | 1.69(1.18) | 3.23(2.39) | <19 |
| Isotretinoin | 366 | 38.85(34.58, 43.65) | 38.07(33.85, 42.82) | 4.91(4.75) | 30.17(27.37) | <19 |
| Docosanol | 7 | 41.02(19.33, 87.07) | 39.93(19.34, 82.46) | 5.31(4.3) | 39.77(21.18) | <19 |
| Depakene | 7 | 7.91(3.76, 16.65) | 7.87(3.74, 16.57) | 2.97(1.97) | 7.84(4.21) | <19 |
| Triflucan | 3 | 49.63(15.69, 157.01) | 48.03(15.72, 146.79) | 5.58(4.14) | 47.95(18.29) | <19 |
| Rifampicin | 4 | 7.56(2.83, 20.21) | 7.52(2.82, 20.04) | 2.91(1.64) | 7.51(3.3) | <19 |
| Retin-a | 3 | 47.99(15.18, 151.74) | 46.49(15.21, 142.09) | 5.54(4.09) | 46.42(17.72) | <19 |
| Ortho tri-cyclen | 3 | 27.64(8.81, 86.71) | 27.15(8.88, 82.98) | 4.76(3.33) | 27.1(10.41) | <19 |
| Vfend | 10 | 23.2(12.4, 43.41) | 22.85(12.45, 41.95) | 4.51(3.64) | 22.72(13.45) | <19 |
| Tazocilline | 3 | 17.06(5.46, 53.3) | 16.87(5.52, 51.56) | 4.07(2.65) | 16.85(6.49) | <19 |
| Tegretol | 7 | 4.36(2.07, 9.18) | 4.35(2.07, 9.16) | 2.12(1.11) | 4.34(2.33) | <19 |
| Amikacin | 7 | 8.25(3.92, 17.36) | 8.21(3.9, 17.29) | 3.03(2.03) | 8.18(4.39) | <19 |
| Amoxicillin | 38 | 4.27(3.09, 5.89) | 4.26(3.11, 5.83) | 2.07(1.61) | 4.19(3.2) | <19 |
| Zelitrex | 4 | 79.82(29.14, 218.63) | 75.72(28.98, 197.84) | 6.24(4.93) | 75.55(32.52) | <19 |
| Voriconazole | 28 | 22.98(15.77, 33.48) | 22.64(15.6, 32.86) | 4.48(3.94) | 22.29(16.27) | <19 |
| Gleevec | 3 | 11.49(3.69, 35.83) | 11.41(3.66, 35.56) | 3.51(2.09) | 11.39(4.4) | <19 |
| Sulfamethoxazole/trimethoprim | 13 | 3.74(2.16, 6.45) | 3.73(2.15, 6.46) | 1.89(1.13) | 3.71(2.35) | <19 |
| Oxatomide | 8 | 584.06(256.9, 1327.82) | 417.47(231.88, 751.61) | 8.7(7.62) | 415.53(209) | <19 |
| Betamethasone | 6 | 16.96(7.57, 37.97) | 16.77(7.51, 37.46) | 4.06(2.99) | 16.72(8.52) | <19 |
| Atropine | 3 | 9.9(3.18, 30.85) | 9.84(3.16, 30.67) | 3.3(1.87) | 9.83(3.8) | <19 |
| Urbanyl | 3 | 14.18(4.54, 44.25) | 14.05(4.51, 43.79) | 3.81(2.39) | 14.03(5.41) | <19 |
| Phenobarbital tab | 5 | 13.54(5.61, 32.71) | 13.43(5.56, 32.44) | 3.74(2.58) | 13.39(6.4) | <19 |
| Methotrexate | 74 | 2.61(2.06, 3.29) | 2.6(2.06, 3.29) | 1.34(1.01) | 2.53(2.09) | <19 |
| Zophren | 3 | 9.27(2.98, 28.88) | 9.22(2.96, 28.74) | 3.2(1.78) | 9.2(3.56) | <19 |
| Purinethol | 3 | 12.2(3.91, 38.04) | 12.11(3.89, 37.74) | 3.6(2.17) | 12.09(4.67) | <19 |
| Betamethasone | 3 | 11.77(3.78, 36.7) | 11.68(3.75, 36.4) | 3.54(2.12) | 11.67(4.5) | <19 |
| Carbamazepine | 6 | 4.45(1.99, 9.94) | 4.44(1.99, 9.92) | 2.15(1.07) | 4.43(2.26) | <19 |
| Colchicine | 7 | 16.47(7.81, 34.75) | 16.3(7.74, 34.33) | 4.02(3.01) | 16.24(8.69) | <19 |
| Rifampin | 6 | 10(4.47, 22.35) | 9.94(4.45, 22.2) | 3.31(2.23) | 9.9(5.05) | <19 |
| Mitoxantrone | 5 | 6.64(2.75, 16.01) | 6.62(2.74, 15.99) | 2.72(1.56) | 6.6(3.16) | <19 |
| Cotrimoxazole | 5 | 4.48(1.86, 10.79) | 4.47(1.85, 10.8) | 2.16(1) | 4.46(2.14) | <19 |
| Oxacillin | 3 | 30.76(9.79, 96.6) | 30.14(9.86, 92.12) | 4.91(3.48) | 30.09(11.55) | <19 |
| Ranitidine | 8 | 5.91(2.95, 11.86) | 5.89(2.97, 11.7) | 2.55(1.61) | 5.87(3.28) | <19 |
| Glivec | 4 | 7.86(2.94, 21.03) | 7.82(2.93, 20.84) | 2.97(1.69) | 7.81(3.43) | <19 |
| Trametinib | 13 | 20.07(11.59, 34.77) | 19.82(11.45, 34.31) | 4.3(3.53) | 19.67(12.42) | <19 |
| Carbamazepine | 10 | 9.33(5, 17.4) | 9.28(4.96, 17.38) | 3.21(2.35) | 9.23(5.48) | <19 |
| Omalizumab | 16 | 3.1(1.9, 5.08) | 3.1(1.9, 5.06) | 1.62(0.93) | 3.08(2.04) | <19 |
| Clemastin | 12 | 1951.43(821.18, 4637.36) | 836.9(576.69, 1214.52) | 9.7(8.71) | 831.06(402.8) | <19 |
| Clarithromycin | 6 | 9.04(4.04, 20.19) | 8.99(4.02, 20.08) | 3.16(2.09) | 8.96(4.57) | <19 |
| Xaluprine | 4 | 126.67(45.55, 352.27) | 116.62(45.52, 298.78) | 6.86(5.54) | 116.35(49.44) | <19 |
| Voriconazole | 11 | 10.48(5.78, 19) | 10.41(5.78, 18.74) | 3.37(2.55) | 10.35(6.29) | <19 |
| Purixan | 4 | 13.09(4.89, 35.08) | 12.98(4.87, 34.58) | 3.7(2.42) | 12.96(5.68) | <19 |
| Dextromethorphan | 3 | 12.2(3.91, 38.04) | 12.11(3.89, 37.74) | 3.6(2.17) | 12.09(4.67) | <19 |
| Tiaramide hydrochloride | 4 | 5826.97(650.93, 52162.12) | 1166.19(757.71, 1794.89) | 10.18(8.47) | 1163.48(185.89) | <19 |
| Velaglucerase alfa | 13 | 58.39(33.47, 101.85) | 56.19(33.1, 95.39) | 5.8(5.03) | 55.77(35.01) | <19 |
| Lamotrigine | 66 | 4.23(3.31, 5.4) | 4.22(3.34, 5.34) | 2.05(1.7) | 4.15(3.39) | 19~44 |
| Isotretinoin | 370 | 50.49(45.25, 56.35) | 49.67(44.16, 55.87) | 5.45(5.29) | 43.62(39.79) | 19~44 |
| Docosanol | 45 | 36.93(27.46, 49.68) | 36.44(27.16, 48.89) | 5.17(4.74) | 35.91(28.02) | 19~44 |
| Diflucan | 5 | 6.22(2.58, 14.97) | 6.21(2.57, 15) | 2.63(1.47) | 6.2(2.97) | 19~44 |
| Viramune | 3 | 5.58(1.8, 17.32) | 5.57(1.79, 17.36) | 2.48(1.06) | 5.56(2.15) | 19~44 |
| Potassium chloride | 5 | 4.28(1.78, 10.31) | 4.28(1.77, 10.34) | 2.1(0.94) | 4.27(2.05) | 19~44 |
| Albendazole | 3 | 25.01(8.02, 78) | 24.78(8.11, 75.73) | 4.63(3.21) | 24.76(9.56) | 19~44 |
| Depakene | 6 | 6.39(2.87, 14.26) | 6.38(2.86, 14.25) | 2.67(1.6) | 6.37(3.26) | 19~44 |
| Monuril | 3 | 131.71(41.28, 420.18) | 125.48(41.87, 376.06) | 6.97(5.51) | 125.36(47.49) | 19~44 |
| Solupred | 3 | 13.55(4.36, 42.17) | 13.49(4.33, 42.05) | 3.75(2.33) | 13.48(5.21) | 19~44 |
| Triflucan | 3 | 28.32(9.07, 88.4) | 28.03(9.17, 85.67) | 4.81(3.38) | 28.01(10.8) | 19~44 |
| Cogentin | 3 | 5.35(1.72, 16.6) | 5.34(1.71, 16.64) | 2.41(1) | 5.33(2.07) | 19~44 |
| Aleve | 7 | 15.05(7.15, 31.65) | 14.97(7.11, 31.53) | 3.9(2.9) | 14.93(8.01) | 19~44 |
| Prezista | 7 | 8.71(4.14, 18.31) | 8.69(4.13, 18.3) | 3.12(2.11) | 8.67(4.66) | 19~44 |
| Compazine | 4 | 6.71(2.51, 17.92) | 6.7(2.51, 17.85) | 2.74(1.47) | 6.69(2.94) | 19~44 |
| Desyrel | 4 | 20.63(7.71, 55.2) | 20.47(7.68, 54.54) | 4.35(3.08) | 20.45(8.97) | 19~44 |
| Marinol | 4 | 20.83(7.78, 55.75) | 20.67(7.76, 55.07) | 4.37(3.1) | 20.65(9.06) | 19~44 |
| Zidovudine | 9 | 15.07(7.82, 29.05) | 14.99(7.85, 28.62) | 3.9(3) | 14.95(8.63) | 19~44 |
| Etravirine | 9 | 79.98(41.17, 155.39) | 77.66(40.67, 148.29) | 6.27(5.36) | 77.43(44.42) | 19~44 |
| Raltegravir | 7 | 29.03(13.77, 61.19) | 28.72(13.64, 60.48) | 4.84(3.83) | 28.66(15.36) | 19~44 |
| Ritonavir | 5 | 6.32(2.63, 15.21) | 6.31(2.61, 15.24) | 2.65(1.5) | 6.3(3.02) | 19~44 |
| Lomotil | 3 | 7.75(2.49, 24.07) | 7.73(2.48, 24.09) | 2.95(1.53) | 7.72(2.99) | 19~44 |
| Darunavir | 4 | 7.58(2.84, 20.23) | 7.56(2.84, 20.14) | 2.92(1.65) | 7.55(3.32) | 19~44 |
| Isoniazid | 4 | 4.56(1.71, 12.17) | 4.55(1.71, 12.12) | 2.19(0.92) | 4.55(2) | 19~44 |
| Rifampicin | 6 | 6.74(3.02, 15.02) | 6.72(3.01, 15.01) | 2.75(1.67) | 6.71(3.43) | 19~44 |
| Pyrazinamide | 4 | 6.46(2.42, 17.25) | 6.45(2.42, 17.19) | 2.69(1.42) | 6.44(2.83) | 19~44 |
| Delsym | 3 | 18.81(6.04, 58.61) | 18.69(6, 58.25) | 4.22(2.8) | 18.67(7.22) | 19~44 |
| Doxycycline | 10 | 4.36(2.34, 8.11) | 4.35(2.32, 8.14) | 2.12(1.26) | 4.34(2.58) | 19~44 |
| Diprivan | 3 | 8.99(2.89, 27.94) | 8.96(2.87, 27.93) | 3.16(1.74) | 8.95(3.47) | 19~44 |
| Cefazolin | 3 | 5.29(1.7, 16.43) | 5.28(1.69, 16.46) | 2.4(0.98) | 5.28(2.04) | 19~44 |
| Retin-a | 5 | 39.58(16.35, 95.78) | 39.01(16.47, 92.41) | 5.28(4.12) | 38.94(18.59) | 19~44 |
| Amaryl | 3 | 10.25(3.3, 31.87) | 10.21(3.28, 31.82) | 3.35(1.93) | 10.2(3.95) | 19~44 |
| Ortho tri-cyclen | 5 | 16.27(6.75, 39.22) | 16.18(6.7, 39.09) | 4.01(2.85) | 16.15(7.73) | 19~44 |
| Minocin | 3 | 28.22(9.04, 88.09) | 27.93(9.14, 85.36) | 4.8(3.38) | 27.91(10.77) | 19~44 |
| Vfend | 4 | 9.51(3.56, 25.4) | 9.48(3.56, 25.26) | 3.24(1.97) | 9.47(4.16) | 19~44 |
| Doripenem monohydrate | 3 | 439.02(129.25, 1491.22) | 376.45(133.22, 1063.78) | 8.55(7.02) | 376.07(135.18) | 19~44 |
| Clarithromycin | 7 | 5.84(2.78, 12.27) | 5.83(2.77, 12.28) | 2.54(1.54) | 5.82(3.13) | 19~44 |
| Zeclar | 3 | 84.07(26.62, 265.52) | 81.5(26.67, 249.09) | 6.35(4.9) | 81.42(31.1) | 19~44 |
| Cephalexin | 4 | 5.27(1.98, 14.08) | 5.27(1.98, 14.04) | 2.39(1.13) | 5.26(2.31) | 19~44 |
| Nystatin | 7 | 16.1(7.65, 33.87) | 16(7.6, 33.7) | 4(2.99) | 15.97(8.57) | 19~44 |
| Victrelis | 3 | 19.51(6.26, 60.78) | 19.37(6.21, 60.37) | 4.27(2.85) | 19.36(7.48) | 19~44 |
| Aclasta | 3 | 31.48(10.08, 98.33) | 31.12(10.18, 95.11) | 4.96(3.53) | 31.09(11.99) | 19~44 |
| Valacyclovir | 7 | 9(4.28, 18.93) | 8.98(4.26, 18.91) | 3.16(2.16) | 8.96(4.81) | 19~44 |
| Mercaptopurine | 3 | 6.07(1.95, 18.87) | 6.06(1.94, 18.89) | 2.6(1.18) | 6.06(2.35) | 19~44 |
| Ultravist | 3 | 12.4(3.99, 38.59) | 12.35(3.96, 38.49) | 3.63(2.21) | 12.34(4.77) | 19~44 |
| Afatinib | 4 | 31.27(11.66, 83.87) | 30.92(11.6, 82.38) | 4.95(3.67) | 30.88(13.53) | 19~44 |
| Nicotine polacrilex | 4 | 15.94(5.96, 42.64) | 15.85(5.95, 42.23) | 3.98(2.71) | 15.83(6.95) | 19~44 |
| Vancomycin sandoz | 3 | 202.62(62.58, 656.04) | 188.22(62.8, 564.09) | 7.55(6.08) | 188.03(70.36) | 19~44 |
| Aracytine | 3 | 22.9(7.35, 71.41) | 22.72(7.29, 70.81) | 4.5(3.08) | 22.69(8.76) | 19~44 |
| Tienam | 3 | 21.36(6.85, 66.56) | 21.19(6.8, 66.04) | 4.4(2.98) | 21.17(8.18) | 19~44 |
| Amiklin | 3 | 94.08(29.72, 297.76) | 90.87(29.73, 277.72) | 6.5(5.06) | 90.77(34.62) | 19~44 |
| Tazocilline | 3 | 16.13(5.18, 50.2) | 16.03(5.14, 49.96) | 4(2.58) | 16.02(6.19) | 19~44 |
| Depo-medrol | 3 | 14.88(4.78, 46.31) | 14.8(4.75, 46.13) | 3.89(2.47) | 14.79(5.72) | 19~44 |
| Depakin chrono | 3 | 46.76(14.92, 146.52) | 45.96(15.04, 140.47) | 5.52(4.09) | 45.92(17.66) | 19~44 |
| Opdivo | 6 | 9.3(4.17, 20.74) | 9.27(4.15, 20.71) | 3.21(2.14) | 9.25(4.73) | 19~44 |
| Yervoy | 4 | 10.07(3.77, 26.89) | 10.03(3.76, 26.72) | 3.32(2.06) | 10.02(4.4) | 19~44 |
| Terbinafine | 5 | 21.64(8.97, 52.22) | 21.47(8.89, 51.87) | 4.42(3.26) | 21.44(10.26) | 19~44 |
| Laroxyl | 5 | 23.32(9.66, 56.3) | 23.13(9.57, 55.88) | 4.53(3.37) | 23.09(11.05) | 19~44 |
| Isoniazid. | 4 | 4.68(1.75, 12.49) | 4.67(1.75, 12.44) | 2.22(0.96) | 4.67(2.05) | 19~44 |
| Avamys | 3 | 17.25(5.54, 53.72) | 17.15(5.5, 53.45) | 4.1(2.68) | 17.13(6.62) | 19~44 |
| Fusidic acid | 3 | 33.34(10.67, 104.18) | 32.94(10.78, 100.67) | 5.04(3.61) | 32.91(12.68) | 19~44 |
| Tri-sprintec | 4 | 20.07(7.5, 53.72) | 19.93(7.48, 53.1) | 4.32(3.04) | 19.91(8.73) | 19~44 |
| Kisqali | 9 | 13.03(6.76, 25.12) | 12.98(6.8, 24.78) | 3.69(2.8) | 12.94(7.47) | 19~44 |
| Kesimpta | 10 | 3.43(1.84, 6.39) | 3.43(1.83, 6.42) | 1.77(0.92) | 3.42(2.03) | 19~44 |
| Ruxience | 14 | 22.59(13.33, 38.28) | 22.41(13.2, 38.04) | 4.48(3.74) | 22.31(14.35) | 19~44 |
| Dapsone | 17 | 35.65(22.06, 57.6) | 35.19(21.99, 56.33) | 5.13(4.46) | 34.99(23.42) | 19~44 |
| Crisaborole | 22 | 2082.99(1190.33, 3645.08) | 1166.92(852.8, 1596.75) | 10.18(9.47) | 1158.29(725.23) | 19~44 |
| Hydrocortisone valerate | 14 | 638.18(355.62, 1145.25) | 514.29(321.3, 823.19) | 9(8.2) | 511.87(313.81) | 19~44 |
| Paraffin | 11 | 558.72(291.25, 1071.83) | 461.34(271.76, 783.16) | 8.84(7.95) | 459.64(266.49) | 19~44 |
| Valacyclovir hydrochloride | 7 | 14.1(6.7, 29.67) | 14.03(6.66, 29.55) | 3.81(2.8) | 14(7.52) | 19~44 |
| Azathioprin | 3 | 42.26(13.5, 132.29) | 41.61(13.61, 127.17) | 5.38(3.95) | 41.57(16) | 19~44 |
| Lamotrigine | 55 | 6.28(4.81, 8.19) | 6.27(4.77, 8.25) | 2.63(2.25) | 6.19(4.96) | 44~59 |
| Isotretinoin | 24 | 33.6(22.44, 50.32) | 33.21(22.44, 49.15) | 5.05(4.47) | 33.02(23.55) | 44~59 |
| Docosanol | 38 | 45.48(32.96, 62.77) | 44.76(32.71, 61.25) | 5.47(5.01) | 44.35(33.87) | 44~59 |
| Diflucan | 5 | 5.27(2.19, 12.67) | 5.26(2.18, 12.71) | 2.39(1.24) | 5.25(2.52) | 44~59 |
| Viramune | 4 | 11.44(4.28, 30.57) | 11.4(4.28, 30.37) | 3.51(2.24) | 11.39(5.01) | 44~59 |
| Depakene | 4 | 6.83(2.56, 18.24) | 6.82(2.56, 18.17) | 2.77(1.5) | 6.81(3) | 44~59 |
| Vfend | 5 | 8.21(3.41, 19.75) | 8.19(3.39, 19.78) | 3.03(1.87) | 8.18(3.92) | 44~59 |
| Nystatin | 5 | 6.34(2.63, 15.25) | 6.33(2.62, 15.29) | 2.66(1.5) | 6.32(3.03) | 44~59 |
| Victrelis | 7 | 8.76(4.17, 18.41) | 8.73(4.15, 18.39) | 3.12(2.12) | 8.72(4.68) | 44~59 |
| Tazocilline | 5 | 17.59(7.3, 42.4) | 17.48(7.24, 42.23) | 4.13(2.97) | 17.46(8.36) | 44~59 |
| Rifater | 3 | 51.87(16.54, 162.63) | 50.92(16.66, 155.63) | 5.67(4.24) | 50.88(19.56) | 44~59 |
| Ribavirin | 54 | 3.62(2.77, 4.74) | 3.62(2.75, 4.76) | 1.84(1.46) | 3.58(2.86) | 44~59 |
| Trastuzumab | 51 | 4.48(3.4, 5.91) | 4.47(3.4, 5.88) | 2.15(1.75) | 4.43(3.51) | 44~59 |
| Peginterferon alfa-2a | 37 | 4.73(3.42, 6.54) | 4.72(3.38, 6.59) | 2.23(1.77) | 4.69(3.58) | 44~59 |
| Radiation therapy | 3 | 8.76(2.82, 27.24) | 8.74(2.8, 27.24) | 3.13(1.71) | 8.73(3.38) | 44~59 |
| Tamiflu | 4 | 6.16(2.31, 16.44) | 6.15(2.31, 16.39) | 2.62(1.35) | 6.14(2.7) | 44~59 |
| Aloxi | 6 | 8.18(3.67, 18.23) | 8.15(3.65, 18.2) | 3.03(1.95) | 8.14(4.16) | 44~59 |
| Tegretol | 13 | 6.29(3.65, 10.84) | 6.27(3.62, 10.85) | 2.65(1.89) | 6.26(3.97) | 44~59 |
| Sunitinib malate | 16 | 4.34(2.65, 7.09) | 4.33(2.65, 7.07) | 2.11(1.42) | 4.32(2.86) | 44~59 |
| Nexavar | 12 | 7.9(4.48, 13.93) | 7.88(4.46, 13.91) | 2.97(2.19) | 7.86(4.89) | 44~59 |
| Fluorouracil | 32 | 3.31(2.34, 4.69) | 3.31(2.33, 4.71) | 1.72(1.22) | 3.29(2.46) | 44~59 |
| Bevacizumab | 22 | 6.88(4.52, 10.47) | 6.87(4.55, 10.37) | 2.77(2.18) | 6.83(4.81) | 44~59 |
| Oxaliplatin | 17 | 4.96(3.08, 8) | 4.96(3.1, 7.94) | 2.3(1.64) | 4.94(3.32) | 44~59 |
| Tykerb | 13 | 12.09(7, 20.86) | 12.04(6.95, 20.84) | 3.59(2.83) | 12(7.6) | 44~59 |
| Aredia | 7 | 19.92(9.46, 41.92) | 19.78(9.39, 41.66) | 4.3(3.3) | 19.75(10.59) | 44~59 |
| Altabax | 3 | 157.61(49.2, 504.87) | 149.06(49.74, 446.73) | 7.22(5.75) | 148.95(56.23) | 44~59 |
| Tenormin | 4 | 6.75(2.53, 18) | 6.73(2.53, 17.93) | 2.75(1.48) | 6.73(2.96) | 44~59 |
| Zometa | 14 | 5.14(3.04, 8.7) | 5.14(3.03, 8.73) | 2.36(1.62) | 5.12(3.3) | 44~59 |
| Navelbine | 7 | 20.56(9.77, 43.27) | 20.41(9.69, 42.98) | 4.35(3.34) | 20.38(10.93) | 44~59 |
| Femara | 5 | 5.42(2.25, 13.05) | 5.42(2.24, 13.09) | 2.44(1.28) | 5.41(2.59) | 44~59 |
| Capecitabine | 43 | 5.25(3.89, 7.09) | 5.24(3.91, 7.03) | 2.38(1.95) | 5.2(4.04) | 44~59 |
| Taxol | 15 | 8.87(5.34, 14.74) | 8.85(5.32, 14.73) | 3.14(2.43) | 8.82(5.76) | 44~59 |
| Champix | 5 | 6.34(2.63, 15.25) | 6.33(2.62, 15.29) | 2.66(1.5) | 6.32(3.03) | 44~59 |
| Loxonin | 4 | 6.37(2.39, 17.01) | 6.36(2.39, 16.95) | 2.67(1.4) | 6.35(2.79) | 44~59 |
| Concentrated red cells | 7 | 294.49(134.96, 642.62) | 265.96(131.33, 538.59) | 8.05(7) | 265.5(138.2) | 44~59 |
| Allopurinol | 12 | 3.64(2.07, 6.42) | 3.64(2.06, 6.43) | 1.86(1.07) | 3.63(2.26) | 44~59 |
| Acitretin | 3 | 12.8(4.12, 39.83) | 12.75(4.09, 39.74) | 3.67(2.25) | 12.74(4.93) | 44~59 |
| Rocephin | 6 | 11.97(5.37, 26.71) | 11.92(5.34, 26.62) | 3.57(2.5) | 11.91(6.08) | 44~59 |
| Lithium carbonate | 5 | 4.48(1.86, 10.78) | 4.48(1.85, 10.82) | 2.16(1) | 4.47(2.15) | 44~59 |
| Pyostacine | 3 | 37.08(11.86, 115.91) | 36.6(11.98, 111.86) | 5.19(3.77) | 36.57(14.09) | 44~59 |
| Indapamide | 4 | 9.39(3.52, 25.07) | 9.36(3.51, 24.94) | 3.23(1.96) | 9.35(4.11) | 44~59 |
| Magnesium oxide | 5 | 5.17(2.15, 12.44) | 5.16(2.14, 12.47) | 2.37(1.21) | 5.16(2.47) | 44~59 |
| Sucralfate | 3 | 7.73(2.49, 24.02) | 7.71(2.47, 24.03) | 2.95(1.53) | 7.71(2.98) | 44~59 |
| Fortum | 4 | 61.06(22.66, 164.56) | 59.75(22.87, 156.11) | 5.9(4.62) | 59.69(26.04) | 44~59 |
| Ciflox | 6 | 57.76(25.72, 129.72) | 56.58(25.83, 123.92) | 5.82(4.74) | 56.5(28.71) | 44~59 |
| Amikacin | 4 | 9.28(3.47, 24.77) | 9.25(3.47, 24.65) | 3.21(1.94) | 9.24(4.06) | 44~59 |
| Flagyl | 6 | 5.52(2.48, 12.3) | 5.51(2.47, 12.31) | 2.46(1.39) | 5.5(2.81) | 44~59 |
| Intron a | 3 | 9.51(3.06, 29.55) | 9.48(3.04, 29.55) | 3.24(1.83) | 9.47(3.67) | 44~59 |
| Lamisil | 4 | 9.09(3.41, 24.28) | 9.07(3.4, 24.17) | 3.18(1.91) | 9.06(3.98) | 44~59 |
| Relpax | 5 | 7(2.91, 16.84) | 6.98(2.89, 16.86) | 2.8(1.64) | 6.97(3.34) | 44~59 |
| Denavir | 3 | 143.78(45.01, 459.3) | 136.64(45.59, 409.51) | 7.09(5.63) | 136.54(51.67) | 44~59 |
| Panitumumab | 6 | 11.63(5.21, 25.95) | 11.59(5.19, 25.89) | 3.53(2.46) | 11.57(5.91) | 44~59 |
| Irinotecan hydrochloride | 10 | 28.09(15.06, 52.42) | 27.82(14.86, 52.09) | 4.79(3.94) | 27.75(16.47) | 44~59 |
| Kytril | 7 | 14.4(6.85, 30.29) | 14.33(6.8, 30.18) | 3.84(2.84) | 14.31(7.68) | 44~59 |
| Corticosteroids | 4 | 9.94(3.72, 26.56) | 9.91(3.72, 26.4) | 3.31(2.04) | 9.9(4.35) | 44~59 |
| Nauzelin | 3 | 20.64(6.63, 64.31) | 20.5(6.58, 63.89) | 4.36(2.93) | 20.48(7.91) | 44~59 |
| Visipaque | 5 | 14.6(6.06, 35.18) | 14.53(6.01, 35.1) | 3.86(2.7) | 14.51(6.95) | 44~59 |
| Amoban | 5 | 53.38(22.02, 129.42) | 52.38(22.11, 124.08) | 5.71(4.54) | 52.31(24.93) | 44~59 |
| Pantosin | 15 | 415.16(240.98, 715.22) | 360.66(225.32, 577.28) | 8.49(7.73) | 359.32(227.94) | 44~59 |
| Sennoside | 3 | 14.11(4.53, 43.88) | 14.04(4.5, 43.76) | 3.81(2.39) | 14.03(5.43) | 44~59 |
| Fluconazole | 9 | 4.56(2.37, 8.78) | 4.56(2.39, 8.71) | 2.19(1.29) | 4.55(2.63) | 44~59 |
| Incivek | 12 | 5.87(3.33, 10.35) | 5.86(3.32, 10.35) | 2.55(1.76) | 5.84(3.63) | 44~59 |
| Everolimus | 22 | 5.3(3.48, 8.06) | 5.29(3.51, 7.98) | 2.4(1.81) | 5.27(3.71) | 44~59 |
| Stivarga | 7 | 6.13(2.92, 12.88) | 6.12(2.91, 12.89) | 2.61(1.61) | 6.11(3.28) | 44~59 |
| Vancomycine | 5 | 48.63(20.07, 117.82) | 47.8(20.18, 113.23) | 5.58(4.41) | 47.74(22.77) | 44~59 |
| Telaprevir | 3 | 5.17(1.67, 16.06) | 5.17(1.66, 16.11) | 2.37(0.95) | 5.16(2) | 44~59 |
| Tyverb | 8 | 131.82(64.8, 268.15) | 125.8(63.35, 249.81) | 6.97(6) | 125.55(69.31) | 44~59 |
| Temesta | 5 | 16.33(6.77, 39.35) | 16.24(6.72, 39.23) | 4.02(2.86) | 16.22(7.77) | 44~59 |
| Prednisolon | 4 | 7.27(2.72, 19.4) | 7.25(2.72, 19.32) | 2.86(1.59) | 7.24(3.19) | 44~59 |
| Emperal | 4 | 390.36(136.86, 1113.42) | 341.69(136.01, 858.43) | 8.42(7.06) | 341.35(142.01) | 44~59 |
| Corodil | 4 | 218.6(78.91, 605.57) | 202.48(79.03, 518.76) | 7.66(6.34) | 202.28(86.24) | 44~59 |
| Lapatinib ditosylate | 5 | 719.27(268.43, 1927.28) | 569.63(260.08, 1247.61) | 9.15(7.87) | 568.92(249.39) | 44~59 |
| Vinorelbine tartrate | 7 | 61.35(28.99, 129.84) | 60.03(29.07, 123.97) | 5.91(4.89) | 59.92(32) | 44~59 |
| Pegintron | 6 | 4.25(1.91, 9.47) | 4.25(1.9, 9.49) | 2.08(1.01) | 4.24(2.17) | 44~59 |
| Fosaprepitant dimeglumine | 4 | 59.08(21.93, 159.16) | 57.85(22.14, 151.15) | 5.85(4.57) | 57.8(25.22) | 44~59 |
| Primperan | 12 | 18.14(10.27, 32.03) | 18.03(10.21, 31.83) | 4.17(3.38) | 17.98(11.17) | 44~59 |
| Magnesium sulfate | 4 | 8.37(3.14, 22.36) | 8.35(3.13, 22.25) | 3.06(1.79) | 8.34(3.67) | 44~59 |
| Mannitol. | 4 | 28.39(10.6, 76.06) | 28.11(10.55, 74.9) | 4.81(3.54) | 28.08(12.31) | 44~59 |
| Magnesium oxide | 14 | 1917.55(967.88, 3799.03) | 1128.38(747.65, 1702.99) | 10.14(9.26) | 1124.46(634.59) | 44~59 |
| Terazosin | 3 | 14.08(4.53, 43.81) | 14.01(4.5, 43.67) | 3.81(2.39) | 14(5.42) | 44~59 |
| Forxiga | 6 | 11.41(5.12, 25.46) | 11.37(5.09, 25.4) | 3.51(2.43) | 11.35(5.8) | 44~59 |
| Flucloxacillin | 6 | 11.71(5.25, 26.12) | 11.66(5.22, 26.04) | 3.54(2.47) | 11.65(5.95) | 44~59 |
| Fucibet | 6 | 546.78(227.46, 1314.39) | 455.82(220.72, 941.33) | 8.83(7.66) | 455.14(218.48) | 44~59 |
| Vectibix | 4 | 6.03(2.26, 16.1) | 6.02(2.26, 16.04) | 2.59(1.32) | 6.02(2.65) | 44~59 |
| Irinotecan | 17 | 8.92(5.53, 14.37) | 8.89(5.55, 14.23) | 3.15(2.48) | 8.86(5.94) | 44~59 |
| Valcyte | 4 | 11.6(4.34, 30.99) | 11.56(4.34, 30.8) | 3.53(2.26) | 11.55(5.07) | 44~59 |
| Palbociclib | 39 | 4.52(3.29, 6.19) | 4.51(3.3, 6.17) | 2.16(1.71) | 4.48(3.44) | 44~59 |
| Mepolizumab | 12 | 14.96(8.47, 26.4) | 14.88(8.43, 26.27) | 3.89(3.1) | 14.84(9.23) | 44~59 |
| Fluticasone propionate | 3 | 6.36(2.05, 19.75) | 6.34(2.03, 19.76) | 2.66(1.25) | 6.34(2.46) | 44~59 |
| Adepal | 3 | 204.89(63.36, 662.59) | 190.66(63.62, 571.4) | 7.57(6.1) | 190.52(71.36) | 44~59 |
| Captopril. | 6 | 27.02(12.08, 60.42) | 26.77(11.99, 59.79) | 4.74(3.66) | 26.73(13.63) | 44~59 |
| Ilomedine | 3 | 745.05(207.77, 2671.66) | 585.61(215.52, 1591.22) | 9.19(7.6) | 585.18(201.02) | 44~59 |
| Sarilumab | 4 | 30.45(11.36, 81.6) | 30.12(11.3, 80.25) | 4.91(3.64) | 30.09(13.19) | 44~59 |
| Imvexxy | 3 | 14.03(4.51, 43.66) | 13.97(4.48, 43.54) | 3.8(2.38) | 13.96(5.4) | 44~59 |
| Tafinlar | 5 | 5.15(2.14, 12.39) | 5.14(2.13, 12.42) | 2.36(1.2) | 5.14(2.47) | 44~59 |
| Diffu k | 4 | 16.05(6, 42.91) | 15.96(5.99, 42.52) | 4(2.73) | 15.95(7) | 44~59 |
| Cymevan | 4 | 99.36(36.62, 269.6) | 95.91(36.71, 250.59) | 6.58(5.29) | 95.82(41.57) | 44~59 |
| Tyvaso | 13 | 4.97(2.88, 8.57) | 4.96(2.87, 8.59) | 2.31(1.55) | 4.95(3.14) | 44~59 |
| Rupatadine fumarate | 3 | 36.42(11.65, 113.84) | 35.96(11.77, 109.9) | 5.17(3.74) | 35.93(13.85) | 44~59 |
| Faslodex | 5 | 4.46(1.85, 10.73) | 4.46(1.85, 10.77) | 2.15(1) | 4.45(2.14) | 44~59 |
| Cabozantinib s-malate | 14 | 5.5(3.25, 9.3) | 5.49(3.23, 9.32) | 2.45(1.72) | 5.48(3.53) | 44~59 |
| Tukysa | 3 | 11.57(3.72, 35.99) | 11.53(3.7, 35.94) | 3.53(2.11) | 11.52(4.46) | 44~59 |
| Ceftriaxone sodium | 14 | 74.61(43.84, 126.99) | 72.66(43.65, 120.95) | 6.18(5.44) | 72.41(46.4) | 44~59 |
| Hydrochlorothiazide/ramipril | 6 | 156.22(68.58, 355.87) | 147.83(67.5, 323.78) | 7.21(6.1) | 147.61(74.12) | 44~59 |
| Ebastine | 6 | 20.2(9.04, 45.12) | 20.06(8.98, 44.81) | 4.32(3.25) | 20.03(10.22) | 44~59 |
| Pegfilgrastim | 4 | 5.13(1.92, 13.7) | 5.13(1.93, 13.67) | 2.36(1.09) | 5.12(2.25) | 44~59 |
| Lormetazepam | 4 | 8.6(3.22, 22.96) | 8.57(3.22, 22.83) | 3.1(1.83) | 8.57(3.77) | 44~59 |
| Fruquintinib | 3 | 22.58(7.24, 70.36) | 22.4(7.19, 69.82) | 4.48(3.06) | 22.38(8.65) | 44~59 |
| Bimzelx | 3 | 15.91(5.11, 49.52) | 15.83(5.08, 49.34) | 3.98(2.56) | 15.82(6.12) | 44~59 |
| Lamotrigine | 35 | 4.63(3.32, 6.46) | 4.63(3.32, 6.46) | 2.21(1.73) | 4.61(3.49) | >=60 |
| Isotretinoin | 9 | 35.27(18.26, 68.12) | 34.78(18.21, 66.41) | 5.12(4.22) | 34.75(20.03) | >=60 |
| Docosanol | 29 | 22.62(15.68, 32.62) | 22.42(15.45, 32.54) | 4.48(3.96) | 22.36(16.46) | >=60 |
| Triflucan | 3 | 10.26(3.3, 31.9) | 10.22(3.28, 31.85) | 3.35(1.94) | 10.22(3.96) | >=60 |
| Vfend | 8 | 3.78(1.89, 7.57) | 3.78(1.9, 7.51) | 1.92(0.97) | 3.78(2.11) | >=60 |
| Ultravist | 6 | 9.82(4.4, 21.89) | 9.78(4.38, 21.84) | 3.29(2.22) | 9.77(5) | >=60 |
| Afatinib | 42 | 19.08(14.08, 25.87) | 18.94(14.12, 25.41) | 4.24(3.8) | 18.86(14.62) | >=60 |
| Tazocilline | 5 | 7.2(2.99, 17.32) | 7.18(2.97, 17.34) | 2.84(1.69) | 7.17(3.44) | >=60 |
| Trastuzumab | 54 | 3.78(2.89, 4.94) | 3.78(2.87, 4.97) | 1.91(1.53) | 3.76(3.01) | >=60 |
| Sunitinib malate | 59 | 4.95(3.83, 6.4) | 4.94(3.83, 6.37) | 2.3(1.93) | 4.92(3.97) | >=60 |
| Tykerb | 14 | 10.93(6.46, 18.48) | 10.88(6.41, 18.47) | 3.44(2.71) | 10.87(7) | >=60 |
| Capecitabine | 61 | 3.31(2.57, 4.25) | 3.3(2.56, 4.26) | 1.72(1.36) | 3.29(2.66) | >=60 |
| Taxol | 13 | 4.55(2.64, 7.85) | 4.55(2.63, 7.88) | 2.18(1.43) | 4.54(2.88) | >=60 |
| Loxonin | 12 | 5.33(3.03, 9.4) | 5.32(3.01, 9.39) | 2.41(1.62) | 5.32(3.31) | >=60 |
| Acitretin | 3 | 8.41(2.71, 26.15) | 8.39(2.69, 26.15) | 3.07(1.65) | 8.39(3.25) | >=60 |
| Rocephin | 7 | 4.69(2.23, 9.85) | 4.68(2.22, 9.86) | 2.23(1.23) | 4.68(2.52) | >=60 |
| Ciflox | 3 | 7.56(2.43, 23.49) | 7.54(2.42, 23.5) | 2.91(1.5) | 7.54(2.92) | >=60 |
| Flagyl | 8 | 3.69(1.84, 7.38) | 3.68(1.85, 7.31) | 1.88(0.94) | 3.68(2.06) | >=60 |
| Lamisil | 4 | 4.97(1.86, 13.25) | 4.96(1.86, 13.22) | 2.31(1.04) | 4.96(2.18) | >=60 |
| Panitumumab | 9 | 5.66(2.94, 10.89) | 5.65(2.96, 10.79) | 2.5(1.6) | 5.64(3.26) | >=60 |
| Kytril | 5 | 4.62(1.92, 11.1) | 4.61(1.91, 11.14) | 2.2(1.05) | 4.61(2.21) | >=60 |
| Incivek | 4 | 4.62(1.73, 12.32) | 4.61(1.73, 12.28) | 2.2(0.94) | 4.61(2.03) | >=60 |
| Everolimus | 71 | 7.95(6.29, 10.05) | 7.93(6.27, 10.03) | 2.98(2.64) | 7.88(6.48) | >=60 |
| Stivarga | 15 | 4.86(2.93, 8.07) | 4.85(2.91, 8.07) | 2.28(1.57) | 4.85(3.17) | >=60 |
| Lapatinib ditosylate | 5 | 225.23(90.08, 563.16) | 206.23(88.78, 479.04) | 7.69(6.48) | 206.12(95.74) | >=60 |
| Vinorelbine tartrate | 8 | 47.71(23.69, 96.08) | 46.81(23.57, 92.95) | 5.55(4.59) | 46.77(26.04) | >=60 |
| Primperan | 8 | 4.08(2.04, 8.17) | 4.08(2.05, 8.1) | 2.03(1.08) | 4.07(2.28) | >=60 |
| Palbociclib | 105 | 3.2(2.64, 3.88) | 3.2(2.63, 3.89) | 1.67(1.39) | 3.17(2.7) | >=60 |
| Sarilumab | 5 | 17.86(7.41, 43.06) | 17.74(7.34, 42.85) | 4.15(2.99) | 17.73(8.49) | >=60 |
| Cabozantinib s-malate | 34 | 3.27(2.33, 4.58) | 3.27(2.34, 4.56) | 1.7(1.22) | 3.26(2.46) | >=60 |
| Lormetazepam | 7 | 6.95(3.31, 14.6) | 6.93(3.29, 14.59) | 2.79(1.79) | 6.93(3.72) | >=60 |
| Fruquintinib | 3 | 11.56(3.72, 35.95) | 11.51(3.69, 35.87) | 3.52(2.11) | 11.51(4.45) | >=60 |
| Zithromax | 8 | 3.87(1.93, 7.74) | 3.87(1.95, 7.68) | 1.95(1.01) | 3.86(2.16) | >=60 |
| Erbitux | 16 | 4.4(2.69, 7.19) | 4.4(2.7, 7.18) | 2.13(1.45) | 4.39(2.91) | >=60 |
| Amoxicillin | 78 | 3.1(2.48, 3.87) | 3.1(2.5, 3.85) | 1.62(1.3) | 3.08(2.55) | >=60 |
| Ursodiol | 10 | 6.9(3.71, 12.84) | 6.88(3.67, 12.88) | 2.78(1.93) | 6.88(4.09) | >=60 |
| Colgate total whitening | 3 | 383.99(113.61, 1297.85) | 331.77(115.13, 956.08) | 8.37(6.84) | 331.66(119.71) | >=60 |
| Adalat | 15 | 7.76(4.67, 12.88) | 7.74(4.65, 12.88) | 2.95(2.24) | 7.73(5.05) | >=60 |
| Piqray | 6 | 6.72(3.01, 14.97) | 6.7(3, 14.96) | 2.74(1.67) | 6.7(3.42) | >=60 |
| Mobocertinib | 14 | 56.9(33.48, 96.71) | 55.63(33.42, 92.6) | 5.8(5.06) | 55.55(35.64) | >=60 |
| Imiquimod | 4 | 41.22(15.34, 110.77) | 40.55(15.22, 108.04) | 5.34(4.06) | 40.54(17.73) | >=60 |
| Zyvox | 5 | 4.29(1.78, 10.32) | 4.29(1.78, 10.36) | 2.1(0.94) | 4.29(2.06) | >=60 |
| Ciprofloxacin hydrochloride | 4 | 5.68(2.13, 15.16) | 5.67(2.13, 15.11) | 2.5(1.24) | 5.67(2.49) | >=60 |
| Pristinamycin | 3 | 22.45(7.2, 69.98) | 22.25(7.28, 68) | 4.48(3.05) | 22.25(8.59) | >=60 |
| Quetiapine fumarate | 59 | 2.57(1.99, 3.32) | 2.56(1.98, 3.3) | 1.35(0.99) | 2.56(2.06) | >=60 |
| Soma | 5 | 6.56(2.73, 15.78) | 6.54(2.71, 15.8) | 2.71(1.55) | 6.54(3.14) | >=60 |
| Depas | 8 | 7.68(3.84, 15.38) | 7.66(3.86, 15.21) | 2.94(1.99) | 7.65(4.28) | >=60 |
| Thyroid tab | 4 | 6.61(2.48, 17.65) | 6.6(2.48, 17.59) | 2.72(1.45) | 6.6(2.9) | >=60 |
| Colchimax | 3 | 16.97(5.45, 52.83) | 16.86(5.41, 52.55) | 4.07(2.65) | 16.85(6.52) | >=60 |
| Gleevec | 11 | 3.62(2.01, 6.55) | 3.62(2.01, 6.52) | 1.86(1.04) | 3.62(2.21) | >=60 |
| Celectol | 3 | 16.39(5.27, 51.04) | 16.29(5.23, 50.77) | 4.03(2.61) | 16.29(6.3) | >=60 |
| Irinotecan hcl | 6 | 4.4(1.97, 9.8) | 4.39(1.97, 9.81) | 2.13(1.06) | 4.39(2.25) | >=60 |
| Etodolac | 5 | 9.16(3.8, 22.04) | 9.13(3.78, 22.06) | 3.19(2.03) | 9.12(4.37) | >=60 |
| Quinine | 12 | 11.87(6.73, 20.93) | 11.82(6.7, 20.87) | 3.56(2.77) | 11.8(7.34) | >=60 |
| Fludex | 3 | 10.02(3.22, 31.15) | 9.98(3.2, 31.11) | 3.32(1.9) | 9.98(3.86) | >=60 |
| Oflocet | 4 | 8.88(3.33, 23.72) | 8.86(3.33, 23.61) | 3.15(1.88) | 8.85(3.89) | >=60 |
| Wellbutrin sr | 3 | 7.38(2.38, 22.94) | 7.36(2.36, 22.94) | 2.88(1.46) | 7.36(2.85) | >=60 |
| Sulfamethoxazole/trimethoprim | 44 | 3.92(2.91, 5.27) | 3.91(2.91, 5.25) | 1.96(1.54) | 3.9(3.04) | >=60 |
| Selbex | 8 | 8.86(4.42, 17.73) | 8.83(4.45, 17.53) | 3.14(2.2) | 8.82(4.93) | >=60 |
| Nicorette | 7 | 7.77(3.7, 16.32) | 7.75(3.68, 16.32) | 2.95(1.95) | 7.74(4.16) | >=60 |
| Immucyst | 3 | 7.06(2.27, 21.94) | 7.04(2.26, 21.94) | 2.82(1.4) | 7.04(2.73) | >=60 |
| Vancomycin hcl | 5 | 6.84(2.84, 16.47) | 6.83(2.83, 16.5) | 2.77(1.61) | 6.82(3.27) | >=60 |
| Primperan tab | 5 | 14.65(6.08, 35.31) | 14.57(6.03, 35.2) | 3.86(2.71) | 14.56(6.98) | >=60 |
| Dalacine | 5 | 22.52(9.33, 54.34) | 22.32(9.24, 53.92) | 4.48(3.32) | 22.31(10.68) | >=60 |
| Fungizone | 3 | 5.76(1.85, 17.88) | 5.75(1.84, 17.92) | 2.52(1.11) | 5.75(2.23) | >=60 |
| Erlotinib hydrochloride | 4 | 6.05(2.27, 16.13) | 6.03(2.26, 16.07) | 2.59(1.33) | 6.03(2.65) | >=60 |
| Durotep | 3 | 13.44(4.32, 41.8) | 13.37(4.29, 41.67) | 3.74(2.32) | 13.36(5.17) | >=60 |
| Duphalac | 9 | 10.52(5.46, 20.25) | 10.48(5.49, 20.01) | 3.39(2.49) | 10.47(6.05) | >=60 |
| Foraseq | 5 | 13.39(5.56, 32.27) | 13.33(5.52, 32.2) | 3.74(2.58) | 13.32(6.38) | >=60 |
| Oxinorm | 4 | 7.82(2.93, 20.87) | 7.8(2.93, 20.78) | 2.96(1.7) | 7.8(3.43) | >=60 |
| Pydoxal | 5 | 17.13(7.11, 41.29) | 17.02(7.05, 41.12) | 4.09(2.93) | 17.01(8.15) | >=60 |
| Sorafenib | 5 | 4.59(1.91, 11.04) | 4.58(1.9, 11.06) | 2.2(1.04) | 4.58(2.2) | >=60 |
| Carbocisteine | 8 | 7.36(3.67, 14.73) | 7.34(3.7, 14.58) | 2.87(1.93) | 7.33(4.1) | >=60 |
| Benicar hct | 6 | 9.04(4.05, 20.15) | 9.01(4.03, 20.12) | 3.17(2.1) | 9(4.6) | >=60 |
| Opalmon | 3 | 8.12(2.61, 25.24) | 8.1(2.6, 25.25) | 3.02(1.6) | 8.1(3.14) | >=60 |
| Torisel | 3 | 6.34(2.04, 19.7) | 6.33(2.03, 19.73) | 2.66(1.25) | 6.33(2.45) | >=60 |
| Berocca c | 3 | 1215.98(304.06, 4862.85) | 810.99(322.81, 2037.45) | 9.66(7.99) | 810.73(254.21) | >=60 |
| Leflunomide | 15 | 4.12(2.48, 6.83) | 4.11(2.47, 6.84) | 2.04(1.33) | 4.11(2.69) | >=60 |
| Rovamycine | 4 | 24.14(9.01, 64.65) | 23.91(8.97, 63.71) | 4.58(3.31) | 23.9(10.48) | >=60 |
| Discotrine | 3 | 16.89(5.42, 52.58) | 16.78(5.38, 52.3) | 4.07(2.65) | 16.77(6.49) | >=60 |
| Elplat | 11 | 9.69(5.36, 17.52) | 9.65(5.36, 17.37) | 3.27(2.45) | 9.64(5.87) | >=60 |
| Progesterone | 6 | 11.41(5.12, 25.46) | 11.36(5.09, 25.37) | 3.51(2.43) | 11.36(5.8) | >=60 |
| Coniel | 3 | 5.36(1.73, 16.64) | 5.35(1.72, 16.67) | 2.42(1) | 5.35(2.07) | >=60 |
| Lecicarbon | 3 | 36.3(11.61, 113.52) | 35.78(11.71, 109.35) | 5.16(3.73) | 35.77(13.78) | >=60 |
| Azunol | 4 | 18.22(6.81, 48.73) | 18.09(6.79, 48.2) | 4.18(2.91) | 18.08(7.94) | >=60 |
| Thyradin | 3 | 8.57(2.76, 26.64) | 8.55(2.74, 26.65) | 3.09(1.68) | 8.54(3.31) | >=60 |
| Hirudoid | 4 | 7.22(2.71, 19.28) | 7.2(2.7, 19.18) | 2.85(1.58) | 7.2(3.17) | >=60 |
| Urso | 6 | 4.55(2.04, 10.14) | 4.54(2.03, 10.14) | 2.18(1.11) | 4.54(2.32) | >=60 |
| Vinorelbine | 6 | 6.18(2.77, 13.77) | 6.17(2.76, 13.78) | 2.62(1.55) | 6.16(3.15) | >=60 |
| Hydroxocobalamin | 5 | 5.76(2.4, 13.87) | 5.75(2.38, 13.89) | 2.52(1.37) | 5.75(2.76) | >=60 |
| Zyclara | 3 | 33.16(10.61, 103.64) | 32.73(10.71, 100.03) | 5.03(3.61) | 32.72(12.61) | >=60 |
| Coumadine | 7 | 4.48(2.14, 9.41) | 4.48(2.13, 9.43) | 2.16(1.16) | 4.47(2.41) | >=60 |
| Ossopan | 3 | 331.63(99.24, 1108.19) | 291.95(101.31, 841.33) | 8.19(6.67) | 291.86(106.36) | >=60 |
| Indocollyre | 3 | 152(47.34, 488.07) | 143.11(47.75, 428.9) | 7.16(5.69) | 143.07(53.9) | >=60 |
| Isophane insulin | 3 | 7.01(2.26, 21.79) | 7(2.25, 21.82) | 2.81(1.39) | 7(2.71) | >=60 |
| Sovriad | 6 | 17.13(7.67, 38.25) | 17.02(7.62, 38.02) | 4.09(3.01) | 17.01(8.69) | >=60 |
| Cetuximab | 15 | 7.95(4.79, 13.21) | 7.93(4.76, 13.2) | 2.99(2.28) | 7.92(5.18) | >=60 |
| Acenocoumarol | 7 | 6.74(3.21, 14.17) | 6.73(3.2, 14.17) | 2.75(1.75) | 6.72(3.61) | >=60 |
| Advair diskus | 13 | 3.42(1.98, 5.89) | 3.42(1.98, 5.92) | 1.77(1.01) | 3.41(2.16) | >=60 |
| Minomycin | 3 | 15.72(5.05, 48.94) | 15.63(5.01, 48.72) | 3.97(2.55) | 15.62(6.04) | >=60 |
| Simeprevir | 3 | 8.37(2.69, 26) | 8.34(2.68, 25.99) | 3.06(1.64) | 8.34(3.23) | >=60 |
| Ibrutinib | 80 | 3.85(3.09, 4.8) | 3.85(3.1, 4.78) | 1.93(1.62) | 3.82(3.18) | >=60 |
| Nystatin | 11 | 5.29(2.93, 9.57) | 5.28(2.93, 9.51) | 2.4(1.58) | 5.28(3.22) | >=60 |
| Olysio | 5 | 12.95(5.38, 31.2) | 12.89(5.34, 31.14) | 3.69(2.53) | 12.88(6.17) | >=60 |
| Propoxyphene | 3 | 47.07(15.01, 147.57) | 46.2(15.12, 141.2) | 5.53(4.1) | 46.18(17.75) | >=60 |
| Nabumetone | 3 | 7.69(2.47, 23.88) | 7.67(2.46, 23.91) | 2.94(1.52) | 7.66(2.97) | >=60 |
| Minocycline hydrochloride | 3 | 16.93(5.44, 52.7) | 16.82(5.4, 52.42) | 4.07(2.65) | 16.81(6.5) | >=60 |
| Ispaghula extract/psyllium | 6 | 51.76(23.05, 116.22) | 50.7(23.15, 111.04) | 5.66(4.58) | 50.67(25.75) | >=60 |
| Amitriptyline hydrochloride. | 5 | 6.27(2.61, 15.08) | 6.26(2.59, 15.12) | 2.64(1.49) | 6.25(3) | >=60 |
| Chlorphenamine | 5 | 8.76(3.64, 21.09) | 8.73(3.61, 21.09) | 3.13(1.97) | 8.73(4.19) | >=60 |
| Hypromellose | 5 | 11.71(4.86, 28.19) | 11.65(4.82, 28.14) | 3.54(2.38) | 11.65(5.58) | >=60 |
| Nystan | 4 | 64.43(23.87, 173.93) | 62.79(24.03, 164.05) | 5.97(4.69) | 62.77(27.34) | >=60 |
| Tetracycline | 4 | 12.06(4.51, 32.21) | 12(4.5, 31.97) | 3.58(2.32) | 12(5.27) | >=60 |
| Cetuximab | 6 | 7.8(3.5, 17.39) | 7.78(3.48, 17.38) | 2.96(1.89) | 7.77(3.98) | >=60 |
| Secukinumab | 62 | 4.34(3.38, 5.58) | 4.34(3.36, 5.6) | 2.11(1.75) | 4.31(3.5) | >=60 |
| Panvitan | 3 | 13(4.18, 40.45) | 12.94(4.15, 40.33) | 3.69(2.27) | 12.94(5.01) | >=60 |
| Humulin nos | 3 | 5.19(1.67, 16.11) | 5.18(1.66, 16.14) | 2.37(0.96) | 5.18(2.01) | >=60 |
| Cravit | 6 | 6.55(2.94, 14.61) | 6.54(2.93, 14.61) | 2.71(1.64) | 6.54(3.34) | >=60 |
| Zinc | 5 | 4.82(2, 11.58) | 4.81(1.99, 11.62) | 2.27(1.11) | 4.81(2.31) | >=60 |
| Fosinopril | 4 | 7.87(2.95, 21.01) | 7.85(2.95, 20.92) | 2.97(1.7) | 7.85(3.45) | >=60 |
| Flecaine | 4 | 8.44(3.16, 22.54) | 8.42(3.16, 22.43) | 3.07(1.81) | 8.42(3.7) | >=60 |
| Chloraminophene | 4 | 51.2(19.02, 137.87) | 50.17(19.2, 131.08) | 5.65(4.37) | 50.15(21.89) | >=60 |
| Peniramin | 7 | 52.73(24.93, 111.52) | 51.63(25, 106.62) | 5.69(4.68) | 51.59(27.57) | >=60 |
| Cefuroxime | 7 | 7.89(3.76, 16.58) | 7.87(3.74, 16.57) | 2.98(1.97) | 7.86(4.23) | >=60 |
| Septrin | 12 | 39.74(22.46, 70.33) | 39.12(22.16, 69.06) | 5.29(4.5) | 39.07(24.24) | >=60 |
| Stromectol | 8 | 32.66(16.25, 65.63) | 32.24(16.24, 64.02) | 5.01(4.06) | 32.21(17.97) | >=60 |
| Oracilline | 5 | 24.28(10.06, 58.59) | 24.05(9.96, 58.1) | 4.59(3.43) | 24.03(11.5) | >=60 |
| Bisoprolol | 3 | 16.89(5.42, 52.58) | 16.78(5.38, 52.3) | 4.07(2.65) | 16.77(6.49) | >=60 |
| Zejula | 13 | 4.66(2.71, 8.04) | 4.66(2.69, 8.07) | 2.22(1.46) | 4.65(2.95) | >=60 |
| Bipreterax | 4 | 15.04(5.63, 40.19) | 14.95(5.61, 39.83) | 3.9(2.63) | 14.94(6.56) | >=60 |
| Pentacarinat | 4 | 18.39(6.88, 49.19) | 18.26(6.85, 48.65) | 4.19(2.92) | 18.25(8.01) | >=60 |
| Ciprofloxacine | 9 | 50.12(25.89, 97) | 49.13(25.73, 93.81) | 5.62(4.71) | 49.08(28.25) | >=60 |
| Azelastine | 3 | 7.08(2.28, 22) | 7.07(2.27, 22.04) | 2.82(1.4) | 7.06(2.74) | >=60 |
| Betamethasone sodium phosphate | 3 | 18.33(5.89, 57.09) | 18.2(5.84, 56.73) | 4.19(2.76) | 18.2(7.03) | >=60 |
| Sensodyne rapid relief | 3 | 1459.18(348.66, 6106.75) | 912.36(370.34, 2247.64) | 9.83(8.13) | 912.07(275.31) | >=60 |
| Valaciclovir | 8 | 5.05(2.52, 10.11) | 5.04(2.54, 10.01) | 2.33(1.39) | 5.04(2.82) | >=60 |
| Thalidomide | 9 | 3.8(1.97, 7.3) | 3.79(1.98, 7.24) | 1.92(1.03) | 3.79(2.19) | >=60 |
| Neofordex | 7 | 83.9(39.48, 178.27) | 81.13(39.29, 167.55) | 6.34(5.32) | 81.07(43.15) | >=60 |
| Palexia | 4 | 8.03(3.01, 21.43) | 8(3, 21.32) | 3(1.73) | 8(3.52) | >=60 |
| Dalteparin sodium | 5 | 5.99(2.49, 14.42) | 5.98(2.48, 14.45) | 2.58(1.42) | 5.98(2.87) | >=60 |
| Hydrochlorothiazide/valsartan | 8 | 9.8(4.89, 19.62) | 9.76(4.92, 19.38) | 3.29(2.34) | 9.75(5.45) | >=60 |
| Flavoxate hydrochloride | 5 | 296.64(117.2, 750.84) | 264.51(116.13, 602.49) | 8.05(6.82) | 264.37(121.55) | >=60 |
| Ginkgo | 9 | 31.11(16.11, 60.06) | 30.73(16.09, 58.68) | 4.94(4.04) | 30.7(17.71) | >=60 |
| Matricaria recutita | 4 | 1389.84(406.78, 4748.58) | 884.81(403.98, 1937.92) | 9.79(8.28) | 884.43(316.36) | >=60 |
| Metamizole sodium | 4 | 5.8(2.17, 15.48) | 5.79(2.17, 15.43) | 2.53(1.27) | 5.79(2.55) | >=60 |
| Idhifa | 4 | 5.64(2.11, 15.05) | 5.63(2.11, 15) | 2.49(1.23) | 5.63(2.48) | >=60 |
| Izilox | 4 | 76.01(28.09, 205.66) | 73.73(28.22, 192.64) | 6.2(4.92) | 73.7(32.05) | >=60 |
| Meropenem anhydrous | 3 | 73.7(23.36, 232.47) | 71.56(23.41, 218.71) | 6.16(4.72) | 71.54(27.36) | >=60 |
| Tarka | 3 | 9.23(2.97, 28.7) | 9.2(2.95, 28.67) | 3.2(1.78) | 9.2(3.56) | >=60 |
| Rocephine | 5 | 9.65(4.01, 23.24) | 9.62(3.98, 23.24) | 3.27(2.11) | 9.61(4.61) | >=60 |
| Arikayce | 9 | 9.58(4.98, 18.45) | 9.55(5, 18.23) | 3.25(2.36) | 9.54(5.52) | >=60 |
| Folotyn | 35 | 149.05(105.89, 209.79) | 140.52(102.69, 192.28) | 7.13(6.64) | 140.01(105.18) | >=60 |
| Dulcolax pico | 3 | 7295.88(758.82, 70148.34) | 1824.72(1033.58, 3221.43) | 10.83(8.94) | 1824.14(274.54) | >=60 |
| Doxycycline hydrochloride | 3 | 25.42(8.15, 79.3) | 25.17(8.24, 76.93) | 4.65(3.23) | 25.16(9.71) | >=60 |
| Rosuvas | 3 | 607.99(171.54, 2154.88) | 486.59(175.6, 1348.33) | 8.93(7.35) | 486.44(168.74) | >=60 |
| Ibaril | 3 | 3647.94(609.46, 21834.74) | 1459.78(706.87, 3014.66) | 10.51(8.69) | 1459.32(326.53) | >=60 |
| Desoximetason | 3 | 331.63(99.24, 1108.19) | 291.95(101.31, 841.33) | 8.19(6.67) | 291.86(106.36) | >=60 |
| Kredex | 4 | 29.48(11, 79.03) | 29.14(10.94, 77.64) | 4.86(3.59) | 29.13(12.76) | >=60 |
| Oramorph | 9 | 17.19(8.92, 33.12) | 17.08(8.95, 32.61) | 4.09(3.19) | 17.06(9.85) | >=60 |
| Insuline glargine | 3 | 61.31(19.49, 192.85) | 59.83(19.58, 182.86) | 5.9(4.47) | 59.81(22.93) | >=60 |
| Amphotericine b | 4 | 194.58(70.26, 538.87) | 180.24(70.35, 461.78) | 7.49(6.17) | 180.16(76.82) | >=60 |
| Brigatinib | 6 | 12.21(5.47, 27.25) | 12.16(5.44, 27.16) | 3.6(2.53) | 12.15(6.21) | >=60 |
| Austedo | 4 | 4.58(1.72, 12.22) | 4.58(1.72, 12.2) | 2.19(0.93) | 4.57(2.01) | >=60 |
| Relvar ellipta | 4 | 9.99(3.74, 26.67) | 9.95(3.73, 26.51) | 3.31(2.05) | 9.95(4.37) | >=60 |
| Kynmobi | 12 | 14.76(8.36, 26.04) | 14.68(8.32, 25.92) | 3.87(3.09) | 14.66(9.11) | >=60 |
| Regorafenib | 4 | 7.73(2.89, 20.62) | 7.71(2.89, 20.54) | 2.95(1.68) | 7.7(3.39) | >=60 |
| Agiolax | 3 | 291.83(88.1, 966.75) | 260.67(88.7, 766.06) | 8.03(6.52) | 260.59(95.66) | >=60 |
| Sotyktu | 3 | 9.06(2.92, 28.16) | 9.03(2.9, 28.14) | 3.17(1.76) | 9.03(3.5) | >=60 |
| Sodium thiosulfate | 9 | 119.03(60.94, 232.48) | 113.52(59.45, 216.76) | 6.83(5.91) | 113.42(64.78) | >=60 |
| Klor-con m | 4 | 71.54(26.46, 193.39) | 69.52(26.61, 181.64) | 6.12(4.83) | 69.49(30.24) | >=60 |
| Escitalopram oxalate | 6 | 5.05(2.27, 11.25) | 5.04(2.26, 11.26) | 2.33(1.26) | 5.04(2.58) | >=60 |
| Estradiol hemihydrate | 3 | 62.9(19.99, 197.91) | 61.33(20.07, 187.44) | 5.94(4.5) | 61.32(23.5) | >=60 |
| Calquence | 5 | 4.96(2.06, 11.92) | 4.95(2.05, 11.96) | 2.31(1.15) | 4.95(2.37) | >=60 |
| Cyproterone | 6 | 113.15(49.89, 256.6) | 108.17(49.39, 236.92) | 6.76(5.66) | 108.1(54.48) | >=60 |
| Siliq | 15 | 37.42(22.46, 62.34) | 36.87(22.15, 61.37) | 5.2(4.49) | 36.81(24.02) | >=60 |
| Sacituzumab govitecan | 7 | 29.57(14.03, 62.32) | 29.22(13.87, 61.54) | 4.87(3.86) | 29.2(15.65) | >=60 |
| Nuzyra | 3 | 13.39(4.3, 41.64) | 13.32(4.27, 41.52) | 3.73(2.32) | 13.31(5.15) | >=60 |
| Lamotrigine | 39 | 3.58(2.61, 4.91) | 3.58(2.62, 4.9) | 1.83(1.38) | 3.56(2.73) | unknow |
| Isotretinoin | 240 | 37.79(33.18, 43.04) | 37.51(32.7, 43.03) | 5.16(4.97) | 35.79(32.1) | unknow |
| Docosanol | 23 | 17.86(11.85, 26.93) | 17.8(11.79, 26.86) | 4.15(3.57) | 17.73(12.57) | unknow |
| Viramune | 3 | 7.06(2.27, 21.91) | 7.05(2.26, 21.97) | 2.82(1.4) | 7.04(2.73) | unknow |
| Albendazole | 6 | 147.49(65.41, 332.54) | 143.05(65.31, 313.31) | 7.16(6.07) | 142.88(72.36) | unknow |
| Solupred | 4 | 39.48(14.75, 105.66) | 39.16(14.7, 104.34) | 5.29(4.02) | 39.13(17.17) | unknow |
| Triflucan | 3 | 46.24(14.83, 144.22) | 45.8(14.99, 139.98) | 5.52(4.09) | 45.78(17.67) | unknow |
| Clarithromycin | 6 | 7.4(3.32, 16.5) | 7.39(3.31, 16.51) | 2.88(1.81) | 7.39(3.78) | unknow |
| Victrelis | 10 | 14.47(7.78, 26.94) | 14.43(7.71, 27.02) | 3.85(2.99) | 14.41(8.57) | unknow |
| Afatinib | 8 | 13.27(6.63, 26.58) | 13.24(6.67, 26.29) | 3.72(2.78) | 13.22(7.39) | unknow |
| Ribavirin | 32 | 3.78(2.67, 5.35) | 3.77(2.65, 5.36) | 1.91(1.42) | 3.76(2.81) | unknow |
| Peginterferon alfa-2a | 22 | 3.54(2.33, 5.38) | 3.54(2.35, 5.34) | 1.82(1.23) | 3.53(2.48) | unknow |
| Radiation therapy | 4 | 16.86(6.32, 45.03) | 16.81(6.31, 44.79) | 4.07(2.8) | 16.79(7.38) | unknow |
| Aloxi | 3 | 8.94(2.88, 27.75) | 8.92(2.86, 27.8) | 3.16(1.74) | 8.92(3.46) | unknow |
| Fluorouracil | 62 | 10.26(7.98, 13.18) | 10.24(7.94, 13.21) | 3.34(2.98) | 10.12(8.21) | unknow |
| Oxaliplatin | 21 | 10.25(6.67, 15.74) | 10.23(6.65, 15.75) | 3.35(2.74) | 10.19(7.12) | unknow |
| Tykerb | 7 | 7.97(3.79, 16.73) | 7.95(3.77, 16.74) | 2.99(1.99) | 7.95(4.27) | unknow |
| Altabax | 3 | 38.56(12.37, 120.14) | 38.25(12.27, 119.22) | 5.26(3.83) | 38.23(14.77) | unknow |
| Femara | 7 | 6.56(3.12, 13.77) | 6.55(3.11, 13.79) | 2.71(1.71) | 6.54(3.52) | unknow |
| Capecitabine | 41 | 5.34(3.92, 7.26) | 5.33(3.9, 7.29) | 2.41(1.97) | 5.3(4.1) | unknow |
| Acitretin | 4 | 23.56(8.82, 62.95) | 23.45(8.8, 62.48) | 4.55(3.28) | 23.43(10.29) | unknow |
| Sucralfate | 3 | 8.03(2.59, 24.95) | 8.02(2.57, 25) | 3(1.59) | 8.02(3.11) | unknow |
| Panitumumab | 8 | 20.13(10.05, 40.34) | 20.05(10.1, 39.81) | 4.32(3.38) | 20.02(11.19) | unknow |
| Incivek | 6 | 4.88(2.19, 10.87) | 4.87(2.18, 10.88) | 2.28(1.21) | 4.87(2.49) | unknow |
| Everolimus | 40 | 7.57(5.54, 10.33) | 7.56(5.52, 10.34) | 2.91(2.46) | 7.5(5.78) | unknow |
| Pegintron | 6 | 8.51(3.82, 18.96) | 8.49(3.8, 18.96) | 3.08(2.01) | 8.49(4.34) | unknow |
| Vectibix | 23 | 35.12(23.28, 52.98) | 34.87(23.1, 52.63) | 5.12(4.54) | 34.71(24.61) | unknow |
| Irinotecan | 11 | 7.05(3.9, 12.74) | 7.04(3.91, 12.67) | 2.81(1.99) | 7.02(4.28) | unknow |
| Cabozantinib s-malate | 28 | 5.09(3.51, 7.38) | 5.08(3.5, 7.37) | 2.34(1.81) | 5.06(3.71) | unknow |
| Tukysa | 5 | 7.86(3.27, 18.91) | 7.85(3.25, 18.96) | 2.97(1.82) | 7.85(3.76) | unknow |
| Valtrex | 11 | 5.17(2.86, 9.35) | 5.17(2.87, 9.31) | 2.37(1.55) | 5.16(3.15) | unknow |
| Keflex | 4 | 5.76(2.16, 15.37) | 5.76(2.16, 15.35) | 2.52(1.26) | 5.75(2.53) | unknow |
| Folic acid | 82 | 3.08(2.47, 3.83) | 3.08(2.48, 3.82) | 1.61(1.29) | 3.04(2.53) | unknow |
| Zithromax | 7 | 5.7(2.71, 11.96) | 5.69(2.7, 11.98) | 2.51(1.51) | 5.68(3.06) | unknow |
| Zovirax | 4 | 7.51(2.82, 20.05) | 7.5(2.81, 19.98) | 2.91(1.64) | 7.5(3.3) | unknow |
| Erbitux | 19 | 13.73(8.74, 21.55) | 13.69(8.72, 21.49) | 3.77(3.14) | 13.64(9.35) | unknow |
| Temazepam | 4 | 5.16(1.94, 13.76) | 5.16(1.94, 13.75) | 2.37(1.1) | 5.15(2.27) | unknow |
| Glyburide | 4 | 5.29(1.99, 14.12) | 5.29(1.99, 14.09) | 2.4(1.14) | 5.29(2.33) | unknow |
| Cardensiel | 3 | 31.94(10.26, 99.45) | 31.73(10.18, 98.9) | 4.99(3.57) | 31.72(12.26) | unknow |
| Mucosta | 4 | 18.63(6.98, 49.75) | 18.56(6.97, 49.45) | 4.21(2.94) | 18.54(8.15) | unknow |
| Pulmicort | 3 | 12.19(3.92, 37.86) | 12.16(3.9, 37.9) | 3.6(2.19) | 12.15(4.71) | unknow |
| Atarax | 5 | 8.65(3.6, 20.81) | 8.64(3.58, 20.87) | 3.11(1.95) | 8.63(4.14) | unknow |
| Evista | 5 | 4.69(1.95, 11.29) | 4.69(1.94, 11.33) | 2.23(1.07) | 4.69(2.25) | unknow |
| Risedronate sodium | 3 | 5.34(1.72, 16.58) | 5.34(1.71, 16.64) | 2.42(1) | 5.34(2.07) | unknow |
| Tretinoin | 10 | 41.29(22.14, 76.99) | 40.94(21.87, 76.65) | 5.35(4.5) | 40.86(24.26) | unknow |
| Amoxicillin | 26 | 3.07(2.09, 4.52) | 3.07(2.07, 4.54) | 1.61(1.07) | 3.06(2.22) | unknow |
| Imovane | 4 | 12.08(4.53, 32.24) | 12.05(4.52, 32.11) | 3.59(2.32) | 12.04(5.3) | unknow |
| Theophylline | 5 | 9.05(3.76, 21.78) | 9.04(3.74, 21.84) | 3.17(2.02) | 9.03(4.33) | unknow |
| Aldactone | 6 | 4.75(2.13, 10.58) | 4.74(2.12, 10.59) | 2.24(1.17) | 4.74(2.42) | unknow |
| Aromasin | 7 | 12.36(5.88, 25.97) | 12.33(5.85, 25.97) | 3.62(2.62) | 12.32(6.62) | unknow |
| Fluticasone | 9 | 8.4(4.37, 16.16) | 8.39(4.39, 16.02) | 3.07(2.17) | 8.37(4.84) | unknow |
| Elidel | 5 | 17.85(7.41, 42.96) | 17.78(7.36, 42.95) | 4.15(2.99) | 17.77(8.52) | unknow |
| Cordarone | 3 | 6.17(1.99, 19.14) | 6.16(1.98, 19.2) | 2.62(1.21) | 6.16(2.39) | unknow |
| Tarceva | 14 | 3.17(1.87, 5.35) | 3.17(1.87, 5.38) | 1.66(0.93) | 3.16(2.04) | unknow |
| Ursodiol | 4 | 6.76(2.53, 18.02) | 6.75(2.53, 17.99) | 2.75(1.49) | 6.74(2.97) | unknow |
| Metronidazole | 10 | 8.93(4.8, 16.61) | 8.91(4.76, 16.68) | 3.15(2.3) | 8.9(5.29) | unknow |
| Triamterene and hydrochlorothiazide | 3 | 7.15(2.3, 22.18) | 7.14(2.29, 22.25) | 2.83(1.42) | 7.13(2.76) | unknow |
| Zelitrex | 4 | 24.01(8.98, 64.15) | 23.89(8.97, 63.65) | 4.58(3.31) | 23.87(10.49) | unknow |
| Topina | 3 | 104.82(33.37, 329.2) | 102.56(33.56, 313.45) | 6.68(5.24) | 102.5(39.34) | unknow |
