# Supplementary material for: An integrative pharmacovigilance, network toxicology and molecular docking study on drug-induced cheilitis
Source: Front Pharmacol. 2026 Mar 20;17:1757807. doi: 10.3389/fphar.2026.1757807 (PMC13047072; doi:10.3389/fphar.2026.1757807)
Supplement: Supplementary file 8 [file Table4.docx]

**Table S4** Drug-induced cheilitis exhibits signaling differences based on sex.

| **Drug** | **Case Reports** | **ROR (95% CI)** | **PRR (95% CI)** | **IC (IC025)** | **EBGM (EBGM05)** | **Sex** |
| --- | --- | --- | --- | --- | --- | --- |
| Isotretinoin | 444 | 28.5(25.92, 31.34) | 28.2(25.57, 31.1) | 4.78(4.64) | 27.39(25.3) | Female |
| Lamotrigine | 145 | 4.05(3.44, 4.78) | 4.05(3.46, 4.74) | 2.01(1.77) | 4.02(3.51) | Female |
| Docosanol | 114 | 30.3(25.18, 36.47) | 29.95(25.11, 35.73) | 4.89(4.63) | 29.73(25.46) | Female |
| Interferon alfa-2a | 50 | 4.16(3.15, 5.49) | 4.15(3.15, 5.46) | 2.05(1.65) | 4.14(3.28) | Female |
| Amoxicillin | 108 | 2.99(2.47, 3.61) | 2.98(2.45, 3.63) | 1.57(1.3) | 2.97(2.53) | Female |
| Capecitabine | 98 | 3.67(3.01, 4.48) | 3.67(3.02, 4.46) | 1.87(1.58) | 3.65(3.09) | Female |
| Erbitux | 22 | 9.64(6.34, 14.66) | 9.61(6.37, 14.5) | 3.26(2.67) | 9.59(6.76) | Female |
| Betamethasone | 10 | 4.68(2.52, 8.7) | 4.67(2.49, 8.74) | 2.22(1.37) | 4.67(2.78) | Female |
| Diflucan | 14 | 4.06(2.41, 6.87) | 4.06(2.39, 6.89) | 2.02(1.29) | 4.06(2.62) | Female |
| Trastuzumab | 115 | 2.88(2.4, 3.46) | 2.88(2.41, 3.44) | 1.52(1.25) | 2.86(2.45) | Female |
| Ribavirin | 84 | 3.96(3.2, 4.91) | 3.96(3.19, 4.91) | 1.98(1.67) | 3.94(3.3) | Female |
| Gleevec | 20 | 3.7(2.38, 5.73) | 3.69(2.4, 5.68) | 1.88(1.27) | 3.69(2.56) | Female |
| Albendazole | 10 | 57.27(30.59, 107.22) | 56(30.5, 102.82) | 5.81(4.94) | 55.96(33.11) | Female |
| Quinine | 13 | 12.98(7.52, 22.39) | 12.92(7.46, 22.37) | 3.69(2.93) | 12.91(8.18) | Female |
| Tazocilline | 10 | 17.78(9.54, 33.12) | 17.66(9.43, 33.07) | 4.14(3.29) | 17.65(10.48) | Female |
| Lamisil | 9 | 7.13(3.71, 13.72) | 7.11(3.72, 13.58) | 2.83(1.93) | 7.11(4.11) | Female |
| Zovirax | 9 | 4.43(2.3, 8.52) | 4.42(2.31, 8.44) | 2.14(1.25) | 4.42(2.56) | Female |
| Nystatin | 10 | 3.71(1.99, 6.89) | 3.7(1.98, 6.93) | 1.89(1.03) | 3.7(2.2) | Female |
| Fluconazole | 22 | 3.73(2.45, 5.66) | 3.72(2.46, 5.61) | 1.89(1.3) | 3.72(2.62) | Female |
| Triflucan | 10 | 31.43(16.84, 58.66) | 31.05(16.91, 57.01) | 4.96(4.1) | 31.03(18.41) | Female |
| Femara | 17 | 3.08(1.92, 4.96) | 3.08(1.92, 4.93) | 1.62(0.95) | 3.08(2.07) | Female |
| Tykerb | 34 | 8.03(5.73, 11.25) | 8.01(5.74, 11.18) | 3(2.52) | 7.99(6.03) | Female |
| Aredia | 13 | 9.48(5.5, 16.35) | 9.45(5.46, 16.36) | 3.24(2.48) | 9.44(5.98) | Female |
| Taxol | 27 | 4.63(3.17, 6.76) | 4.63(3.19, 6.72) | 2.21(1.67) | 4.62(3.37) | Female |
| Adalat | 12 | 7.65(4.34, 13.49) | 7.63(4.32, 13.47) | 2.93(2.14) | 7.62(4.74) | Female |
| Voriconazole | 9 | 4.57(2.38, 8.79) | 4.57(2.39, 8.73) | 2.19(1.29) | 4.56(2.64) | Female |
| Etravirine | 9 | 46.4(23.99, 89.73) | 45.56(23.86, 86.99) | 5.51(4.61) | 45.54(26.22) | Female |
| Bendroflumethiazide | 9 | 3.6(1.87, 6.92) | 3.59(1.88, 6.85) | 1.84(0.95) | 3.59(2.08) | Female |
| Stromectol | 9 | 26.13(13.55, 50.41) | 25.87(13.55, 49.4) | 4.69(3.79) | 25.85(14.92) | Female |
| Minocycline | 10 | 3.85(2.07, 7.17) | 3.85(2.06, 7.21) | 1.94(1.09) | 3.85(2.29) | Female |
| Colchicine | 13 | 6.09(3.53, 10.5) | 6.08(3.51, 10.53) | 2.6(1.85) | 6.08(3.85) | Female |
| Sunitinib malate | 38 | 5.3(3.86, 7.29) | 5.29(3.87, 7.24) | 2.4(1.95) | 5.28(4.05) | Female |
| Ursodiol | 11 | 4.96(2.74, 8.96) | 4.95(2.75, 8.91) | 2.31(1.49) | 4.95(3.02) | Female |
| Prednisolon | 9 | 4.77(2.48, 9.17) | 4.76(2.49, 9.09) | 2.25(1.35) | 4.76(2.75) | Female |
| Kytril | 10 | 7.86(4.22, 14.62) | 7.84(4.19, 14.68) | 2.97(2.12) | 7.83(4.66) | Female |
| Everolimus | 103 | 7.02(5.79, 8.53) | 7.01(5.76, 8.53) | 2.8(2.52) | 6.97(5.92) | Female |
| Mupirocin | 9 | 6.9(3.58, 13.27) | 6.88(3.6, 13.14) | 2.78(1.89) | 6.88(3.98) | Female |
| Clarithromycin | 17 | 4.11(2.55, 6.62) | 4.1(2.56, 6.56) | 2.04(1.37) | 4.1(2.75) | Female |
| Incivek | 15 | 6.16(3.71, 10.23) | 6.15(3.69, 10.24) | 2.62(1.91) | 6.15(4.02) | Female |
| Nystatin | 14 | 4.53(2.68, 7.65) | 4.52(2.66, 7.67) | 2.18(1.44) | 4.52(2.91) | Female |
| Tyverb | 13 | 53.92(31.12, 93.43) | 52.79(31.1, 89.61) | 5.72(4.96) | 52.75(33.3) | Female |
| Vinorelbine | 9 | 5.14(2.67, 9.9) | 5.14(2.69, 9.81) | 2.36(1.46) | 5.13(2.97) | Female |
| Vinorelbine tartrate | 15 | 47.28(28.36, 78.82) | 46.41(27.88, 77.26) | 5.54(4.82) | 46.37(30.23) | Female |
| Lapatinib ditosylate | 10 | 285.04(148.11, 548.55) | 255.76(142.06, 460.47) | 8(7.1) | 255.59(147.79) | Female |
| Stivarga | 9 | 4.1(2.13, 7.88) | 4.09(2.14, 7.81) | 2.03(1.14) | 4.09(2.37) | Female |
| Afatinib | 34 | 14.64(10.45, 20.52) | 14.56(10.43, 20.32) | 3.86(3.38) | 14.53(10.95) | Female |
| Valacyclovir | 16 | 3.19(1.96, 5.22) | 3.19(1.95, 5.21) | 1.67(0.99) | 3.19(2.11) | Female |
| Primperan | 12 | 6.41(3.64, 11.31) | 6.4(3.63, 11.3) | 2.68(1.89) | 6.4(3.98) | Female |
| Pantosin | 14 | 95.93(56.23, 163.65) | 92.4(55.51, 153.81) | 6.53(5.78) | 92.31(59.04) | Female |
| Magnesium oxide | 14 | 475.71(268.47, 842.94) | 399.32(249.48, 639.17) | 8.64(7.85) | 398.95(247.19) | Female |
| Cabozantinib s-malate | 26 | 3.75(2.55, 5.5) | 3.74(2.53, 5.53) | 1.9(1.36) | 3.74(2.71) | Female |
| Ofev | 16 | 3.16(1.93, 5.16) | 3.16(1.94, 5.16) | 1.66(0.97) | 3.15(2.09) | Female |
| Palbociclib | 146 | 3.01(2.56, 3.54) | 3.01(2.57, 3.52) | 1.58(1.34) | 2.99(2.6) | Female |
| Ibrutinib | 82 | 5.03(4.05, 6.25) | 5.02(4.05, 6.23) | 2.32(2.01) | 5(4.17) | Female |
| Opdivo | 28 | 4.74(3.27, 6.87) | 4.73(3.26, 6.86) | 2.24(1.71) | 4.72(3.46) | Female |
| Mepolizumab | 12 | 3.47(1.97, 6.12) | 3.47(1.97, 6.13) | 1.79(1.01) | 3.47(2.16) | Female |
| Trametinib | 13 | 8.5(4.93, 14.66) | 8.47(4.89, 14.66) | 3.08(2.32) | 8.47(5.37) | Female |
| Rubraca | 22 | 3.65(2.4, 5.54) | 3.64(2.41, 5.49) | 1.86(1.27) | 3.64(2.57) | Female |
| Septrin | 12 | 39.74(22.46, 70.3) | 39.12(22.16, 69.06) | 5.29(4.5) | 39.09(24.25) | Female |
| Ciprofloxacine | 9 | 61.65(31.81, 119.47) | 60.18(31.52, 114.91) | 5.91(5) | 60.14(34.57) | Female |
| Arikayce | 12 | 5.88(3.33, 10.35) | 5.86(3.32, 10.35) | 2.55(1.77) | 5.86(3.65) | Female |
| Piqray | 25 | 7.91(5.34, 11.72) | 7.89(5.33, 11.68) | 2.98(2.42) | 7.88(5.67) | Female |
| Tukysa | 10 | 5.14(2.76, 9.56) | 5.13(2.74, 9.61) | 2.36(1.51) | 5.13(3.05) | Female |
| Oramorph | 9 | 14.5(7.53, 27.93) | 14.42(7.55, 27.53) | 3.85(2.95) | 14.41(8.33) | Female |
| Valacyclovir hydrochloride | 13 | 5.68(3.29, 9.79) | 5.67(3.28, 9.82) | 2.5(1.74) | 5.66(3.59) | Female |
| Tretinoin | 11 | 13.67(7.55, 24.72) | 13.6(7.55, 24.49) | 3.76(2.95) | 13.59(8.27) | Female |
| Dapsone | 18 | 16.41(10.32, 26.1) | 16.31(10.19, 26.11) | 4.03(3.37) | 16.29(11.05) | Female |
| Mobocertinib | 14 | 54.01(31.8, 91.73) | 52.88(31.77, 88.03) | 5.72(4.98) | 52.83(33.91) | Female |
| Ruxience | 14 | 4.35(2.58, 7.36) | 4.35(2.56, 7.38) | 2.12(1.39) | 4.34(2.8) | Female |
| Crisaborole | 22 | 691.14(430.77, 1108.88) | 540.81(372.66, 784.83) | 9.08(8.42) | 540.02(363.59) | Female |
| Hydrocortisone valerate | 14 | 195.1(113.21, 336.22) | 180.94(108.7, 301.2) | 7.5(6.74) | 180.77(114.64) | Female |
| Paraffin | 11 | 81.43(44.66, 148.5) | 78.88(43.81, 142.01) | 6.3(5.47) | 78.82(47.68) | Female |
| Velaglucerase alfa | 13 | 31.74(18.36, 54.86) | 31.35(18.11, 54.27) | 4.97(4.21) | 31.32(19.81) | Female |
| Isotretinoin | 505 | 73.95(67.5, 81.01) | 72.51(65.74, 79.98) | 6.08(5.95) | 67.58(62.61) | Male |
| Lamotrigine | 65 | 5.31(4.16, 6.78) | 5.3(4.19, 6.71) | 2.4(2.05) | 5.26(4.29) | Male |
| Docosanol | 27 | 28.87(19.75, 42.19) | 28.63(19.73, 41.55) | 4.83(4.3) | 28.53(20.77) | Male |
| Interferon alfa-2a | 29 | 2.8(1.94, 4.03) | 2.8(1.93, 4.06) | 1.48(0.96) | 2.79(2.06) | Male |
| Amoxicillin | 72 | 3.73(2.96, 4.7) | 3.73(2.95, 4.72) | 1.89(1.55) | 3.7(3.05) | Male |
| Capecitabine | 51 | 4.85(3.68, 6.39) | 4.84(3.68, 6.37) | 2.27(1.87) | 4.82(3.82) | Male |
| Erbitux | 12 | 3.52(2, 6.21) | 3.52(1.99, 6.21) | 1.81(1.03) | 3.52(2.19) | Male |
| Nystatin | 9 | 7.04(3.66, 13.55) | 7.03(3.68, 13.42) | 2.81(1.92) | 7.02(4.06) | Male |
| Voriconazole | 22 | 10.72(7.05, 16.3) | 10.69(7.08, 16.13) | 3.41(2.82) | 10.66(7.5) | Male |
| Minocycline | 7 | 6.54(3.12, 13.74) | 6.53(3.1, 13.75) | 2.71(1.7) | 6.53(3.51) | Male |
| Sunitinib malate | 44 | 4.69(3.48, 6.3) | 4.68(3.49, 6.28) | 2.22(1.8) | 4.66(3.64) | Male |
| Everolimus | 39 | 4.88(3.56, 6.69) | 4.87(3.56, 6.66) | 2.28(1.83) | 4.85(3.73) | Male |
| Mupirocin | 12 | 23.95(13.57, 42.27) | 23.79(13.48, 42) | 4.57(3.78) | 23.75(14.77) | Male |
| Incivek | 8 | 3.69(1.84, 7.38) | 3.68(1.85, 7.31) | 1.88(0.94) | 3.68(2.06) | Male |
| Stivarga | 13 | 5.65(3.28, 9.74) | 5.64(3.26, 9.76) | 2.49(1.74) | 5.63(3.57) | Male |
| Afatinib | 22 | 22.12(14.54, 33.67) | 21.99(14.57, 33.19) | 4.45(3.86) | 21.93(15.43) | Male |
| Primperan | 8 | 7.42(3.71, 14.85) | 7.4(3.73, 14.69) | 2.89(1.94) | 7.4(4.14) | Male |
| Cabozantinib s-malate | 54 | 4.57(3.5, 5.97) | 4.57(3.47, 6.01) | 2.18(1.8) | 4.54(3.63) | Male |
| Vfend | 21 | 11.64(7.58, 17.88) | 11.61(7.54, 17.87) | 3.53(2.93) | 11.58(8.09) | Male |
| Fluorouracil | 96 | 5.38(4.4, 6.58) | 5.37(4.41, 6.53) | 2.41(2.12) | 5.32(4.49) | Male |
| Zithromax | 11 | 6.24(3.45, 11.28) | 6.23(3.46, 11.22) | 2.64(1.82) | 6.22(3.79) | Male |
| Depakene | 11 | 7.89(4.36, 14.26) | 7.87(4.37, 14.17) | 2.97(2.16) | 7.86(4.79) | Male |
| Tegretol | 18 | 4.97(3.13, 7.9) | 4.96(3.1, 7.94) | 2.31(1.66) | 4.95(3.36) | Male |
| Atarax | 7 | 4.44(2.11, 9.32) | 4.43(2.1, 9.33) | 2.15(1.15) | 4.43(2.38) | Male |
| Oxaliplatin | 34 | 4.93(3.52, 6.91) | 4.93(3.53, 6.88) | 2.3(1.82) | 4.91(3.7) | Male |
| Tarceva | 17 | 4.1(2.55, 6.6) | 4.1(2.56, 6.56) | 2.03(1.36) | 4.09(2.75) | Male |
| Effexor xr | 8 | 4.52(2.26, 9.06) | 4.52(2.28, 8.98) | 2.18(1.23) | 4.52(2.53) | Male |
| Nicorette | 7 | 5.09(2.42, 10.68) | 5.08(2.41, 10.7) | 2.34(1.34) | 5.08(2.73) | Male |
| Loxonin | 14 | 10.91(6.45, 18.44) | 10.88(6.41, 18.47) | 3.44(2.71) | 10.86(7) | Male |
| Mucosta | 7 | 6.99(3.33, 14.67) | 6.97(3.31, 14.68) | 2.8(1.8) | 6.97(3.75) | Male |
| Nexavar | 26 | 5.38(3.66, 7.91) | 5.37(3.63, 7.95) | 2.42(1.88) | 5.36(3.88) | Male |
| Concentrated red cells | 7 | 117.85(55.46, 250.44) | 113.98(55.19, 235.39) | 6.83(5.81) | 113.87(60.6) | Male |
| Rocephin | 8 | 7.05(3.52, 14.12) | 7.04(3.55, 13.98) | 2.81(1.87) | 7.03(3.94) | Male |
| Sulfamethoxazole/trimethoprim | 48 | 4.68(3.52, 6.22) | 4.67(3.55, 6.14) | 2.22(1.81) | 4.65(3.67) | Male |
| Taxotere | 10 | 6.05(3.25, 11.26) | 6.04(3.23, 11.31) | 2.59(1.74) | 6.04(3.59) | Male |
| Duphalac | 9 | 16.66(8.65, 32.08) | 16.58(8.68, 31.66) | 4.05(3.15) | 16.56(9.57) | Male |
| Flagyl | 13 | 7.87(4.56, 13.56) | 7.85(4.53, 13.59) | 2.97(2.21) | 7.84(4.97) | Male |
| Elplat | 11 | 13.57(7.5, 24.54) | 13.52(7.51, 24.34) | 3.75(2.94) | 13.5(8.22) | Male |
| Irinotecan hydrochloride | 13 | 17.46(10.12, 30.13) | 17.38(10.04, 30.09) | 4.12(3.36) | 17.35(10.99) | Male |
| Thalidomide | 11 | 5.69(3.15, 10.28) | 5.68(3.15, 10.23) | 2.5(1.69) | 5.67(3.46) | Male |
| Gemzar | 7 | 4.16(1.98, 8.74) | 4.16(1.98, 8.76) | 2.05(1.05) | 4.15(2.23) | Male |
| Ciflox | 7 | 25.44(12.09, 53.53) | 25.26(11.99, 53.2) | 4.66(3.65) | 25.24(13.54) | Male |
| Valaciclovir | 9 | 8.26(4.29, 15.89) | 8.24(4.32, 15.73) | 3.04(2.15) | 8.23(4.76) | Male |
| Idursulfase | 7 | 5.18(2.47, 10.88) | 5.18(2.46, 10.91) | 2.37(1.37) | 5.17(2.78) | Male |
| Panitumumab | 16 | 13.07(8, 21.37) | 13.02(7.98, 21.25) | 3.7(3.01) | 13(8.62) | Male |
| Pegintron | 15 | 7.14(4.3, 11.85) | 7.12(4.28, 11.85) | 2.83(2.12) | 7.11(4.65) | Male |
| Victrelis | 14 | 12.36(7.31, 20.9) | 12.32(7.26, 20.91) | 3.62(2.89) | 12.3(7.92) | Male |
| Irinotecan | 18 | 4.69(2.95, 7.44) | 4.68(2.92, 7.49) | 2.22(1.57) | 4.67(3.17) | Male |
| Bevacizumab | 24 | 4.8(3.21, 7.17) | 4.79(3.24, 7.09) | 2.26(1.69) | 4.78(3.42) | Male |
| Cetuximab | 15 | 9.04(5.44, 15.02) | 9.02(5.42, 15.01) | 3.17(2.46) | 9(5.89) | Male |
| Vectibix | 25 | 14.53(9.8, 21.54) | 14.47(9.78, 21.41) | 3.85(3.29) | 14.43(10.38) | Male |
| Voriconazole | 12 | 5.82(3.3, 10.26) | 5.81(3.29, 10.26) | 2.54(1.75) | 5.8(3.61) | Male |
| Carbamazepine | 10 | 5.6(3.01, 10.43) | 5.6(2.99, 10.49) | 2.48(1.63) | 5.59(3.33) | Male |
| Peniramin | 7 | 78.57(37.13, 166.26) | 76.83(37.2, 158.67) | 6.26(5.25) | 76.76(41) | Male |
| Neofordex | 7 | 178.09(83.28, 380.85) | 169.36(82.01, 349.75) | 7.4(6.37) | 169.2(89.57) | Male |
| Clemastin | 12 | 284.44(157.8, 512.71) | 262.78(151.79, 454.92) | 8.04(7.22) | 262.35(160.24) | Male |
| Hydrochlorothiazide\valsartan | 8 | 19.38(9.67, 38.84) | 19.28(9.71, 38.29) | 4.27(3.32) | 19.26(10.76) | Male |
| Ginkgo | 9 | 54.72(28.32, 105.77) | 53.88(28.22, 102.88) | 5.75(4.85) | 53.82(31.01) | Male |
| Unspecified ingredient | 11 | 4.38(2.42, 7.91) | 4.37(2.43, 7.87) | 2.13(1.31) | 4.37(2.66) | Male |
| Azathioprine | 11 | 3.73(2.06, 6.73) | 3.72(2.07, 6.7) | 1.89(1.08) | 3.72(2.27) | Male |
| Mesalazine | 8 | 9.39(4.69, 18.8) | 9.37(4.72, 18.61) | 3.23(2.28) | 9.36(5.24) | Male |
| Folotyn | 35 | 204.18(145.08, 287.37) | 192.82(140.92, 263.84) | 7.58(7.1) | 191.91(144.18) | Male |
| Kynmobi | 10 | 19.8(10.63, 36.89) | 19.7(10.52, 36.89) | 4.3(3.44) | 19.67(11.69) | Male |
| Ceftriaxone sodium | 14 | 21.22(12.54, 35.91) | 21.1(12.43, 35.82) | 4.4(3.66) | 21.06(13.56) | Male |
| Sodium thiosulfate | 9 | 213.24(108.72, 418.27) | 200.84(107.27, 376.04) | 7.65(6.72) | 200.59(114.16) | Male |
| Siliq | 15 | 42.8(25.71, 71.25) | 42.29(25.41, 70.4) | 5.4(4.69) | 42.2(27.55) | Male |
| Isotretinoin | 60 | 41.8(32.22, 54.21) | 41.51(32.17, 53.56) | 5.31(4.94) | 39.6(31.86) | Unknown |
| Lamotrigine | 15 | 4.36(2.62, 7.26) | 4.36(2.62, 7.26) | 2.11(1.4) | 4.32(2.82) | Unknown |
| Docosanol | 5 | 10.8(4.49, 26.03) | 10.79(4.47, 26.07) | 3.43(2.27) | 10.75(5.15) | Unknown |
| Interferon alfa-2a | 5 | 5.94(2.47, 14.3) | 5.93(2.45, 14.33) | 2.56(1.41) | 5.91(2.84) | Unknown |
| Ribavirin | 8 | 5.22(2.61, 10.47) | 5.22(2.63, 10.37) | 2.38(1.43) | 5.19(2.9) | Unknown |
| Fluorouracil | 13 | 4.5(2.6, 7.77) | 4.49(2.59, 7.77) | 2.16(1.4) | 4.46(2.82) | Unknown |
| Nicorette | 5 | 9.73(4.04, 23.43) | 9.71(4.02, 23.46) | 3.27(2.12) | 9.68(4.64) | Unknown |
| Irinotecan | 6 | 6.07(2.72, 13.54) | 6.06(2.71, 13.54) | 2.59(1.52) | 6.04(3.08) | Unknown |
| Bevacizumab | 10 | 5.45(2.92, 10.15) | 5.44(2.91, 10.19) | 2.44(1.58) | 5.41(3.21) | Unknown |
| Azathioprine | 4 | 4.93(1.84, 13.15) | 4.92(1.85, 13.11) | 2.3(1.03) | 4.91(2.16) | Unknown |
| Atenolol | 3 | 6.11(1.97, 18.97) | 6.1(1.96, 19.01) | 2.61(1.19) | 6.09(2.36) | Unknown |
| Hydroxyzine | 3 | 5.87(1.89, 18.23) | 5.86(1.88, 18.26) | 2.55(1.13) | 5.85(2.27) | Unknown |
| Celecoxib | 3 | 6.59(2.12, 20.48) | 6.59(2.11, 20.54) | 2.72(1.3) | 6.57(2.55) | Unknown |
| Losartan potassium | 3 | 10.23(3.29, 31.78) | 10.21(3.28, 31.82) | 3.35(1.93) | 10.19(3.94) | Unknown |
| Loperamid | 3 | 726.6(217.89, 2423.04) | 642.88(223.09, 1852.62) | 9.33(7.81) | 641.37(234.12) | Unknown |
| Clindamycin | 3 | 16.64(5.35, 51.76) | 16.6(5.33, 51.74) | 4.05(2.63) | 16.56(6.41) | Unknown |
| Sevredol | 4 | 455.1(163.99, 1262.96) | 420.83(164.26, 1078.18) | 8.71(7.39) | 419.51(178.58) | Unknown |
| Nasal preparations | 4 | 5575.01(1392.74, 22316.23) | 2788.01(1404, 5536.31) | 11.44(9.86) | 2779.26(870.77) | Unknown |
| Abraxane | 4 | 13.23(4.95, 35.35) | 13.2(4.95, 35.17) | 3.72(2.45) | 13.16(5.78) | Unknown |
| Ruxolitinib | 46 | 3.24(2.41, 4.34) | 3.24(2.41, 4.35) | 1.66(1.24) | 3.15(2.47) | Unknown |
| Doxycycline | 3 | 8.59(2.76, 26.68) | 8.57(2.75, 26.71) | 3.1(1.68) | 8.56(3.31) | Unknown |
| Clozapin hexal | 3 | 16711.9(1737.14, 160774.26) | 4178.72(2366.96, 7377.28) | 12.03(10.13) | 4168.89(627.12) | Unknown |
| Hydroxyurea | 5 | 16.77(6.96, 40.42) | 16.72(6.92, 40.39) | 4.06(2.9) | 16.66(7.98) | Unknown |
| Mupirocin | 3 | 18.88(6.07, 58.73) | 18.82(6.04, 58.66) | 4.23(2.81) | 18.78(7.27) | Unknown |
| Marijuana | 4 | 65.2(24.29, 174.99) | 64.46(24.19, 171.75) | 6.01(4.73) | 64.26(28.13) | Unknown |
| Acitretin | 4 | 162.77(60.14, 440.57) | 158.18(60.54, 413.28) | 7.3(6.01) | 157.69(68.54) | Unknown |
| Chapstick nos | 9 | 220.93(113.23, 431.05) | 212.58(111.33, 405.9) | 7.72(6.81) | 211.08(120.66) | Unknown |
| Smoflipid | 4 | 137.65(50.97, 371.77) | 134.36(51.42, 351.05) | 7.07(5.78) | 133.94(58.33) | Unknown |
| Amino acids nos | 4 | 2787.51(838.31, 9268.82) | 1858.67(832.15, 4151.46) | 10.86(9.37) | 1852.84(677.99) | Unknown |
| Heparin | 5 | 4.26(1.77, 10.25) | 4.25(1.76, 10.27) | 2.08(0.93) | 4.24(2.03) | Unknown |
| Motegrity | 4 | 73.35(27.31, 197.01) | 72.41(27.18, 192.93) | 6.17(4.9) | 72.19(31.58) | Unknown |
| Niraparib | 16 | 12.1(7.39, 19.83) | 12.08(7.4, 19.72) | 3.58(2.89) | 11.94(7.9) | Unknown |
| Dovato | 3 | 21.87(7.03, 68.06) | 21.79(6.99, 67.91) | 4.44(3.02) | 21.74(8.41) | Unknown |
| Xcopri | 7 | 4.92(2.34, 10.35) | 4.92(2.34, 10.36) | 2.29(1.29) | 4.9(2.63) | Unknown |
| Imiquimod | 3 | 59.47(19.04, 185.76) | 58.85(19.26, 179.86) | 5.88(4.45) | 58.72(22.64) | Unknown |
| Azithromycin anhydrous. | 9 | 13.57(7.04, 26.16) | 13.54(7.09, 25.85) | 3.75(2.85) | 13.45(7.76) | Unknown |
| Nucala | 7 | 5.54(2.63, 11.64) | 5.53(2.63, 11.65) | 2.46(1.46) | 5.51(2.96) | Unknown |
