# Supplementary material for: An integrative pharmacovigilance, network toxicology and molecular docking study on drug-induced cheilitis
Source: Front Pharmacol. 2026 Mar 20;17:1757807. doi: 10.3389/fphar.2026.1757807 (PMC13047072; doi:10.3389/fphar.2026.1757807)
Supplement: Supplementary file 9 [file Table6.docx]

**Table S6** Results of sensitivity analysis in subgroup.

| Drug | Case Reports | ROR(95% CI) | PRR(95% CI) | IC(IC025) | EBGM(EBGM05) | Group |
| --- | --- | --- | --- | --- | --- | --- |
| **Age** |  |  |  |  |  |  |
| Isotretinoin | 333 | 53.26(47.3, 59.97) | 52.78(46.92, 59.37) | 5.45(5.28) | 43.79(39.65) | <19 |
| Lamotrigine | 55 | 9.85(7.53, 12.89) | 9.84(7.48, 12.95) | 3.26(2.88) | 9.58(7.65) | <19 |
| Ibuprofen | 37 | 3.31(2.39, 4.58) | 3.3(2.36, 4.6) | 1.7(1.24) | 3.26(2.48) | <19 |
| Methotrexate | 34 | 3.57(2.54, 5.01) | 3.57(2.56, 4.98) | 1.82(1.33) | 3.52(2.65) | <19 |
| Docosanol | 29 | 24.67(17.08, 35.63) | 24.55(16.92, 35.63) | 4.6(4.07) | 24.19(17.78) | <19 |
| Voriconazole | 28 | 33(22.69, 47.98) | 32.78(22.59, 47.57) | 5.01(4.48) | 32.32(23.62) | <19 |
| Amoxicillin | 21 | 6.97(4.53, 10.72) | 6.96(4.52, 10.71) | 2.79(2.18) | 6.9(4.81) | <19 |
| Cabozantinib | 18 | 4.24(2.66, 6.75) | 4.24(2.65, 6.79) | 2.07(1.42) | 4.21(2.85) | <19 |
| Valacyclovir | 17 | 15.62(9.68, 25.2) | 15.58(9.73, 24.94) | 3.95(3.28) | 15.45(10.35) | <19 |
| Everolimus | 17 | 4.38(2.71, 7.06) | 4.37(2.73, 6.99) | 2.12(1.45) | 4.34(2.91) | <19 |
| Capecitabine | 15 | 3.37(2.03, 5.6) | 3.37(2.02, 5.61) | 1.74(1.03) | 3.35(2.19) | <19 |
| Carbamazepine | 13 | 6(3.48, 10.35) | 5.99(3.46, 10.37) | 2.57(1.82) | 5.96(3.77) | <19 |
| Trametinib | 11 | 9.58(5.29, 17.34) | 9.56(5.31, 17.21) | 3.25(2.43) | 9.51(5.79) | <19 |
| Oseltamivir | 10 | 5.05(2.71, 9.4) | 5.04(2.69, 9.44) | 2.33(1.47) | 5.02(2.98) | <19 |
| Fluticasone/Vilanterol | 9 | 3.71(1.93, 7.14) | 3.71(1.94, 7.08) | 1.88(0.99) | 3.69(2.13) | <19 |
| Clarithromycin | 8 | 5.22(2.6, 10.45) | 5.21(2.62, 10.35) | 2.38(1.43) | 5.19(2.9) | <19 |
| Ribavirin | 8 | 6.42(3.2, 12.86) | 6.41(3.23, 12.73) | 2.68(1.73) | 6.39(3.57) | <19 |
| Telaprevir | 8 | 4.67(2.33, 9.36) | 4.67(2.35, 9.27) | 2.22(1.27) | 4.65(2.6) | <19 |
| Avobenzone/Homosalate/Octinoxate/Octisalate/Octocrylene/Oxybenzone/Titanium dioxide/Zinc oxide | 8 | 140.98(69.7, 285.19) | 136.98(68.98, 272.01) | 7.09(6.13) | 136.42(75.66) | <19 |
| Afatinib | 7 | 17.06(8.11, 35.87) | 17(8.07, 35.8) | 4.08(3.08) | 16.94(9.09) | <19 |
| Alpelisib | 7 | 6.84(3.25, 14.37) | 6.83(3.24, 14.38) | 2.77(1.76) | 6.81(3.66) | <19 |
| Mesalamine | 6 | 3.98(1.78, 8.87) | 3.97(1.78, 8.87) | 1.99(0.92) | 3.97(2.03) | <19 |
| Petrolatum | 6 | 35.91(16.06, 80.28) | 35.65(15.96, 79.63) | 5.15(4.08) | 35.54(18.13) | <19 |
| Idursulfase | 5 | 7.17(2.98, 17.26) | 7.16(2.96, 17.3) | 2.84(1.68) | 7.15(3.43) | <19 |
| Lapatinib | 5 | 5.95(2.47, 14.33) | 5.95(2.46, 14.37) | 2.57(1.41) | 5.93(2.85) | <19 |
| Panitumumab | 5 | 6.22(2.58, 14.97) | 6.21(2.57, 15) | 2.63(1.48) | 6.2(2.97) | <19 |
| Tucatinib | 5 | 12.51(5.19, 30.12) | 12.48(5.17, 30.15) | 3.64(2.48) | 12.45(5.97) | <19 |
| Azithromycin | 4 | 6.17(2.31, 16.46) | 6.16(2.31, 16.41) | 2.62(1.35) | 6.15(2.71) | <19 |
| Rifampicin | 4 | 9.25(3.47, 24.71) | 9.24(3.47, 24.62) | 3.21(1.94) | 9.22(4.05) | <19 |
| Retapamulin | 4 | 42.52(15.87, 113.9) | 42.15(15.82, 112.31) | 5.39(4.12) | 42.07(18.44) | <19 |
| Isotretinoin | 332 | 47.72(42.45, 53.65) | 47.33(42.08, 53.24) | 5.34(5.17) | 40.38(36.61) | 19-44 |
| Lamotrigine | 75 | 10.56(8.39, 13.3) | 10.54(8.33, 13.33) | 3.35(3.02) | 10.22(8.43) | 19-44 |
| Docosanol | 67 | 38.69(30.31, 49.39) | 38.41(30.36, 48.59) | 5.22(4.87) | 37.27(30.39) | 19-44 |
| Sodium fluoride | 28 | 67.4(46.31, 98.09) | 66.5(45.82, 96.51) | 6.04(5.51) | 65.67(47.97) | 19-44 |
| Cabozantinib | 19 | 4.4(2.8, 6.92) | 4.4(2.8, 6.91) | 2.13(1.49) | 4.37(2.99) | 19-44 |
| Valacyclovir | 17 | 12.39(7.68, 19.98) | 12.36(7.72, 19.78) | 3.62(2.95) | 12.27(8.23) | 19-44 |
| Everolimus | 17 | 4.2(2.61, 6.77) | 4.2(2.62, 6.72) | 2.06(1.39) | 4.17(2.8) | 19-44 |
| Capecitabine | 16 | 3.21(1.97, 5.26) | 3.21(1.97, 5.24) | 1.68(0.99) | 3.2(2.12) | 19-44 |
| Clarithromycin | 16 | 7.96(4.86, 13.02) | 7.95(4.87, 12.98) | 2.98(2.29) | 7.9(5.23) | 19-44 |
| Amoxicillin | 14 | 4.03(2.38, 6.82) | 4.03(2.37, 6.84) | 2(1.27) | 4.01(2.58) | 19-44 |
| Carbamazepine | 10 | 3.83(2.06, 7.13) | 3.83(2.05, 7.17) | 1.93(1.08) | 3.81(2.27) | 19-44 |
| Telaprevir | 10 | 4.72(2.53, 8.78) | 4.71(2.52, 8.82) | 2.23(1.38) | 4.7(2.79) | 19-44 |
| Fluticasone/Vilanterol | 10 | 4.12(2.21, 7.67) | 4.11(2.2, 7.7) | 2.04(1.18) | 4.1(2.44) | 19-44 |
| Fluconazole | 9 | 9.17(4.76, 17.67) | 9.16(4.8, 17.49) | 3.19(2.29) | 9.13(5.27) | 19-44 |
| Ribavirin | 8 | 4.98(2.48, 9.96) | 4.97(2.5, 9.87) | 2.31(1.36) | 4.96(2.77) | 19-44 |
| Ramipril | 8 | 7.17(3.58, 14.37) | 7.16(3.61, 14.22) | 2.84(1.89) | 7.14(3.99) | 19-44 |
| Alpelisib | 8 | 7.8(3.89, 15.63) | 7.79(3.92, 15.47) | 2.96(2.01) | 7.77(4.34) | 19-44 |
| Avobenzone/Homosalate/Octinoxate/Octisalate/Octocrylene/Oxybenzone/Titanium dioxide/Zinc oxide | 8 | 145.7(72.03, 294.72) | 141.51(71.26, 281) | 7.14(6.18) | 141(78.2) | 19-44 |
| Fluorouracil | 7 | 4.87(2.32, 10.22) | 4.86(2.31, 10.24) | 2.28(1.28) | 4.85(2.61) | 19-44 |
| Afatinib | 7 | 15.87(7.55, 33.38) | 15.83(7.52, 33.34) | 3.98(2.98) | 15.78(8.47) | 19-44 |
| Terbinafine | 7 | 19.47(9.26, 40.94) | 19.39(9.21, 40.84) | 4.27(3.27) | 19.34(10.38) | 19-44 |
| Nevirapine | 6 | 5.29(2.37, 11.79) | 5.28(2.36, 11.79) | 2.4(1.33) | 5.27(2.69) | 19-44 |
| Acitretin | 6 | 25.01(11.2, 55.83) | 24.88(11.14, 55.57) | 4.63(3.56) | 24.82(12.67) | 19-44 |
| Lapatinib | 6 | 5.73(2.57, 12.78) | 5.73(2.57, 12.8) | 2.51(1.44) | 5.71(2.92) | 19-44 |
| Interferon alfa-2b | 6 | 5.1(2.29, 11.36) | 5.09(2.28, 11.37) | 2.34(1.27) | 5.08(2.6) | 19-44 |
| Boceprevir | 6 | 12.61(5.65, 28.13) | 12.58(5.63, 28.1) | 3.65(2.58) | 12.55(6.41) | 19-44 |
| Sodium fluoride/Triclosan | 6 | 215.14(94.88, 487.84) | 206.09(94.1, 451.38) | 7.68(6.59) | 205.54(103.61) | 19-44 |
| Voriconazole | 5 | 5.26(2.19, 12.65) | 5.25(2.17, 12.68) | 2.39(1.23) | 5.24(2.52) | 19-44 |
| Allopurinol | 5 | 8.61(3.58, 20.71) | 8.59(3.56, 20.75) | 3.1(1.94) | 8.58(4.11) | 19-44 |
| Panitumumab | 5 | 5.62(2.33, 13.52) | 5.61(2.32, 13.55) | 2.49(1.33) | 5.6(2.69) | 19-44 |
| Isotretinoin | 170 | 35.92(30.71, 42.02) | 35.69(30.51, 41.75) | 5.05(4.82) | 33.08(29.02) | 45-59 |
| Docosanol | 61 | 40.46(31.34, 52.23) | 40.15(31.12, 51.8) | 5.29(4.92) | 39.09(31.57) | 45-59 |
| Lamotrigine | 58 | 10.43(8.04, 13.54) | 10.41(8.07, 13.43) | 3.35(2.97) | 10.17(8.18) | 45-59 |
| Ibuprofen | 33 | 3.06(2.17, 4.31) | 3.06(2.15, 4.35) | 1.6(1.11) | 3.03(2.27) | 45-59 |
| Sodium fluoride | 32 | 75.2(52.91, 106.87) | 74.1(52.07, 105.45) | 6.19(5.69) | 73.07(54.45) | 45-59 |
| Capecitabine | 25 | 3.9(2.63, 5.79) | 3.9(2.64, 5.77) | 1.95(1.39) | 3.87(2.78) | 45-59 |
| Telaprevir | 25 | 6.74(4.54, 9.99) | 6.73(4.55, 9.96) | 2.74(2.18) | 6.66(4.79) | 45-59 |
| Everolimus | 24 | 5.32(3.56, 7.95) | 5.31(3.52, 8.01) | 2.4(1.83) | 5.27(3.76) | 45-59 |
| Cabozantinib | 23 | 4.84(3.21, 7.3) | 4.83(3.2, 7.29) | 2.26(1.68) | 4.8(3.4) | 45-59 |
| Budesonide | 22 | 3.09(2.03, 4.71) | 3.09(2.05, 4.66) | 1.62(1.03) | 3.07(2.16) | 45-59 |
| Interferon alfa-2a | 21 | 4.26(2.77, 6.54) | 4.25(2.76, 6.54) | 2.08(1.47) | 4.22(2.95) | 45-59 |
| Lapatinib | 18 | 12.38(7.78, 19.7) | 12.35(7.72, 19.77) | 3.62(2.96) | 12.26(8.31) | 45-59 |
| Palbociclib | 18 | 3.17(1.99, 5.04) | 3.16(1.97, 5.06) | 1.65(1) | 3.15(2.13) | 45-59 |
| Amoxicillin | 17 | 5.46(3.39, 8.8) | 5.45(3.4, 8.72) | 2.44(1.77) | 5.42(3.63) | 45-59 |
| Fluconazole | 16 | 17.74(10.84, 29.04) | 17.68(10.83, 28.86) | 4.13(3.44) | 17.56(11.63) | 45-59 |
| Valacyclovir | 14 | 10.22(6.04, 17.29) | 10.2(6.01, 17.32) | 3.34(2.61) | 10.14(6.53) | 45-59 |
| Ribavirin | 14 | 5.94(3.51, 10.04) | 5.93(3.49, 10.07) | 2.56(1.83) | 5.9(3.8) | 45-59 |
| Sunitinib | 14 | 4.3(2.54, 7.28) | 4.3(2.53, 7.3) | 2.1(1.36) | 4.28(2.76) | 45-59 |
| Oxaliplatin | 11 | 3.7(2.05, 6.7) | 3.7(2.06, 6.66) | 1.88(1.06) | 3.69(2.25) | 45-59 |
| Stannous fluoride | 11 | 175.02(95.79, 319.77) | 169.09(93.92, 304.43) | 7.39(6.56) | 168.27(101.62) | 45-59 |
| Panitumumab | 11 | 9.99(5.52, 18.07) | 9.97(5.54, 17.95) | 3.31(2.49) | 9.93(6.04) | 45-59 |
| Allopurinol | 10 | 14.44(7.75, 26.9) | 14.4(7.69, 26.96) | 3.84(2.99) | 14.34(8.52) | 45-59 |
| Fluorouracil | 10 | 5.05(2.71, 9.39) | 5.04(2.69, 9.44) | 2.33(1.47) | 5.02(2.99) | 45-59 |
| Interferon alfa-2b | 9 | 5.3(2.75, 10.2) | 5.29(2.77, 10.1) | 2.4(1.5) | 5.28(3.05) | 45-59 |
| Acitretin | 9 | 35.56(18.44, 68.6) | 35.32(18.5, 67.44) | 5.14(4.24) | 35.18(20.3) | 45-59 |
| Fluticasone/Vilanterol | 9 | 3.6(1.87, 6.92) | 3.6(1.89, 6.87) | 1.84(0.95) | 3.59(2.07) | 45-59 |
| Oseltamivir | 8 | 5.63(2.81, 11.28) | 5.63(2.84, 11.18) | 2.49(1.54) | 5.61(3.14) | 45-59 |
| Clarithromycin | 8 | 4.25(2.12, 8.52) | 4.25(2.14, 8.44) | 2.08(1.14) | 4.24(2.37) | 45-59 |
| Doxycycline | 8 | 5.58(2.79, 11.18) | 5.58(2.81, 11.08) | 2.48(1.53) | 5.56(3.11) | 45-59 |
| Avobenzone/Homosalate/Octinoxate/Octisalate/Octocrylene/Oxybenzone/Titanium dioxide/Zinc oxide | 8 | 147.44(72.89, 298.21) | 143.21(72.12, 284.38) | 7.16(6.2) | 142.7(79.15) | 45-59 |
| Isotretinoin | 165 | 32.27(27.57, 37.78) | 32.06(27.41, 37.5) | 4.92(4.7) | 30.35(26.61) | ≥60 |
| Fluticasone | 53 | 3.46(2.63, 4.54) | 3.45(2.62, 4.54) | 1.77(1.38) | 3.41(2.72) | ≥60 |
| Docosanol | 51 | 28.45(21.56, 37.56) | 28.29(21.5, 37.22) | 4.8(4.4) | 27.82(22.05) | ≥60 |
| Lamotrigine | 50 | 8.91(6.73, 11.78) | 8.89(6.76, 11.7) | 3.13(2.73) | 8.76(6.93) | ≥60 |
| Capecitabine | 40 | 4.16(3.04, 5.68) | 4.15(3.03, 5.68) | 2.04(1.59) | 4.11(3.17) | ≥60 |
| Ibrutinib | 39 | 2.78(2.03, 3.81) | 2.78(2.03, 3.8) | 1.46(1.01) | 2.75(2.11) | ≥60 |
| Palbociclib | 39 | 2.97(2.16, 4.07) | 2.97(2.17, 4.06) | 1.56(1.11) | 2.94(2.26) | ≥60 |
| Sodium fluoride | 38 | 69.78(50.55, 96.33) | 68.75(50.24, 94.07) | 6.09(5.63) | 67.89(51.84) | ≥60 |
| Everolimus | 37 | 6.04(4.36, 8.35) | 6.03(4.32, 8.41) | 2.58(2.11) | 5.97(4.55) | ≥60 |
| Cabozantinib | 32 | 4.59(3.24, 6.5) | 4.59(3.23, 6.53) | 2.18(1.69) | 4.55(3.4) | ≥60 |
| Budesonide | 30 | 2.79(1.95, 4) | 2.79(1.96, 3.97) | 1.47(0.96) | 2.77(2.05) | ≥60 |
| Valacyclovir | 24 | 11.2(7.49, 16.75) | 11.18(7.55, 16.55) | 3.47(2.9) | 11.1(7.93) | ≥60 |
| Sunitinib | 23 | 3.84(2.55, 5.78) | 3.83(2.54, 5.78) | 1.93(1.35) | 3.81(2.7) | ≥60 |
| Cetuximab | 22 | 6.35(4.17, 9.66) | 6.34(4.2, 9.57) | 2.66(2.06) | 6.31(4.44) | ≥60 |
| Amoxicillin | 20 | 4.89(3.15, 7.59) | 4.89(3.18, 7.53) | 2.28(1.66) | 4.86(3.36) | ≥60 |
| Erlotinib | 20 | 3.49(2.25, 5.42) | 3.49(2.27, 5.37) | 1.8(1.18) | 3.47(2.4) | ≥60 |
| Afatinib | 20 | 18.78(12.09, 29.18) | 18.71(12.16, 28.8) | 4.22(3.6) | 18.59(12.86) | ≥60 |
| Allopurinol | 15 | 8.65(5.21, 14.37) | 8.64(5.19, 14.38) | 3.1(2.39) | 8.6(5.62) | ≥60 |
| Telaprevir | 15 | 5.08(3.06, 8.45) | 5.08(3.05, 8.46) | 2.34(1.63) | 5.06(3.31) | ≥60 |
| Lapatinib | 14 | 8.97(5.3, 15.17) | 8.95(5.27, 15.19) | 3.16(2.42) | 8.92(5.74) | ≥60 |
| Clarithromycin | 13 | 4.97(2.88, 8.57) | 4.96(2.87, 8.59) | 2.31(1.55) | 4.95(3.13) | ≥60 |
| Stannous fluoride | 12 | 175.56(98.52, 312.84) | 169.07(97.66, 292.69) | 7.4(6.59) | 168.4(103.85) | ≥60 |
| Doxycycline | 12 | 5.86(3.32, 10.34) | 5.86(3.32, 10.35) | 2.55(1.76) | 5.84(3.63) | ≥60 |
| Panitumumab | 12 | 6.71(3.8, 11.83) | 6.7(3.8, 11.83) | 2.74(1.95) | 6.67(4.15) | ≥60 |
| Fluticasone/Vilanterol | 12 | 3.44(1.95, 6.06) | 3.44(1.95, 6.07) | 1.78(0.99) | 3.43(2.13) | ≥60 |
| Sorafenib | 11 | 3.55(1.97, 6.43) | 3.55(1.97, 6.39) | 1.82(1.01) | 3.54(2.16) | ≥60 |
| Simeprevir | 11 | 19.49(10.77, 35.27) | 19.41(10.78, 34.95) | 4.27(3.45) | 19.34(11.77) | ≥60 |
| Alpelisib | 11 | 7.77(4.3, 14.06) | 7.76(4.31, 13.97) | 2.95(2.13) | 7.74(4.71) | ≥60 |
| Linezolid | 10 | 4.79(2.57, 8.92) | 4.79(2.56, 8.97) | 2.26(1.4) | 4.77(2.84) | ≥60 |
| Amoxicillin/Clavulanate potassium | 9 | 11.02(5.73, 21.22) | 11(5.76, 21) | 3.46(2.56) | 10.97(6.34) | ≥60 |
| **Sex** |  |  |  |  |  |  |
| Isotretinoin | 250 | 76.64(66.88, 87.83) | 75.59(65.9, 86.71) | 5.99(5.79) | 63.47(56.63) | Male |
| Lamotrigine | 41 | 12.9(9.46, 17.6) | 12.87(9.41, 17.61) | 3.65(3.21) | 12.55(9.68) | Male |
| Voriconazole | 32 | 21.71(15.28, 30.83) | 21.61(15.19, 30.75) | 4.4(3.91) | 21.18(15.79) | Male |
| Methotrexate | 30 | 3.3(2.3, 4.73) | 3.3(2.32, 4.7) | 1.7(1.19) | 3.25(2.4) | Male |
| Docosanol | 27 | 41.24(28.14, 60.44) | 40.89(28.18, 59.34) | 5.33(4.79) | 40.19(29.19) | Male |
| Amoxicillin | 26 | 7.57(5.14, 11.16) | 7.56(5.11, 11.19) | 2.9(2.35) | 7.45(5.38) | Male |
| Cabozantinib | 24 | 4.22(2.82, 6.31) | 4.21(2.79, 6.35) | 2.06(1.49) | 4.16(2.97) | Male |
| Capecitabine | 22 | 5.05(3.32, 7.7) | 5.05(3.35, 7.62) | 2.32(1.73) | 4.99(3.51) | Male |
| Sunitinib | 21 | 4.46(2.9, 6.87) | 4.46(2.9, 6.86) | 2.14(1.53) | 4.41(3.08) | Male |
| Allopurinol | 15 | 12.96(7.79, 21.57) | 12.93(7.77, 21.52) | 3.68(2.97) | 12.81(8.37) | Male |
| Everolimus | 15 | 4.91(2.95, 8.16) | 4.9(2.94, 8.16) | 2.28(1.57) | 4.86(3.18) | Male |
| Sorafenib | 13 | 4.71(2.73, 8.12) | 4.7(2.71, 8.14) | 2.22(1.46) | 4.67(2.96) | Male |
| Cetuximab | 12 | 4.41(2.5, 7.78) | 4.4(2.49, 7.77) | 2.13(1.34) | 4.38(2.72) | Male |
| Telaprevir | 12 | 3.98(2.26, 7.03) | 3.98(2.25, 7.03) | 1.98(1.2) | 3.96(2.46) | Male |
| Erlotinib | 11 | 3.98(2.2, 7.21) | 3.98(2.21, 7.17) | 1.99(1.17) | 3.96(2.41) | Male |
| Carbamazepine | 10 | 5.23(2.81, 9.74) | 5.23(2.79, 9.79) | 2.38(1.52) | 5.2(3.09) | Male |
| Valacyclovir | 9 | 9.92(5.15, 19.11) | 9.9(5.18, 18.9) | 3.3(2.4) | 9.85(5.69) | Male |
| Panitumumab | 8 | 6.76(3.37, 13.55) | 6.75(3.4, 13.4) | 2.75(1.8) | 6.72(3.76) | Male |
| Interferon alfa-2b | 8 | 5.55(2.77, 11.11) | 5.54(2.79, 11) | 2.46(1.52) | 5.52(3.08) | Male |
| Afatinib | 7 | 15.99(7.6, 33.64) | 15.94(7.57, 33.57) | 3.99(2.98) | 15.87(8.52) | Male |
| Linezolid | 6 | 3.97(1.78, 8.86) | 3.97(1.78, 8.87) | 1.99(0.91) | 3.96(2.02) | Male |
| Moxifloxacin | 6 | 5.38(2.41, 12) | 5.38(2.41, 12.02) | 2.42(1.35) | 5.36(2.74) | Male |
| Boceprevir | 6 | 9.07(4.06, 20.23) | 9.05(4.05, 20.21) | 3.17(2.1) | 9.02(4.61) | Male |
| Fluticasone/Salmeterol | 5 | 5.72(2.38, 13.78) | 5.72(2.37, 13.82) | 2.51(1.35) | 5.7(2.73) | Male |
| Apomorphine | 5 | 14(5.81, 33.73) | 13.96(5.78, 33.72) | 3.8(2.64) | 13.92(6.67) | Male |
| Azithromycin | 4 | 9.06(3.39, 24.2) | 9.05(3.4, 24.11) | 3.17(1.91) | 9.03(3.97) | Male |
| Iopromide | 4 | 16.68(6.24, 44.58) | 16.62(6.24, 44.28) | 4.05(2.78) | 16.58(7.29) | Male |
| Fenofibrate | 4 | 47.08(17.56, 126.24) | 46.61(17.49, 124.19) | 5.54(4.26) | 46.49(20.37) | Male |
| Fluconazole | 4 | 14.85(5.56, 39.68) | 14.81(5.56, 39.46) | 3.88(2.62) | 14.77(6.49) | Male |
| Idursulfase | 3 | 7.2(2.32, 22.36) | 7.19(2.31, 22.41) | 2.84(1.43) | 7.18(2.78) | Male |
| Isotretinoin | 221 | 26.61(23.2, 30.52) | 26.42(23.03, 30.31) | 4.62(4.43) | 24.63(21.96) | Female |
| Docosanol | 110 | 43.25(35.71, 52.37) | 42.72(35.12, 51.97) | 5.37(5.09) | 41.26(35.16) | Female |
| Lamotrigine | 86 | 9.78(7.89, 12.13) | 9.76(7.87, 12.11) | 3.25(2.94) | 9.52(7.95) | Female |
| Sodium fluoride | 59 | 98.21(75.63, 127.53) | 95.48(74, 123.19) | 6.55(6.18) | 93.7(75.3) | Female |
| Fluticasone | 48 | 3.18(2.39, 4.23) | 3.18(2.37, 4.27) | 1.65(1.25) | 3.14(2.48) | Female |
| Everolimus | 38 | 6.13(4.45, 8.44) | 6.12(4.47, 8.37) | 2.6(2.14) | 6.05(4.63) | Female |
| Amoxicillin | 32 | 4.94(3.49, 7) | 4.94(3.47, 7.03) | 2.29(1.8) | 4.9(3.66) | Female |
| Budesonide | 30 | 2.82(1.97, 4.04) | 2.82(1.98, 4.01) | 1.49(0.98) | 2.8(2.07) | Female |
| Alendronate sodium | 29 | 3.29(2.28, 4.74) | 3.29(2.27, 4.77) | 1.71(1.19) | 3.27(2.41) | Female |
| Lapatinib | 28 | 9.11(6.27, 13.22) | 9.09(6.26, 13.19) | 3.17(2.64) | 9.01(6.6) | Female |
| Ibrutinib | 23 | 3.03(2.01, 4.56) | 3.03(2.01, 4.57) | 1.59(1.01) | 3.01(2.14) | Female |
| Clarithromycin | 22 | 6.27(4.12, 9.54) | 6.26(4.15, 9.45) | 2.64(2.05) | 6.23(4.38) | Female |
| Fluconazole | 20 | 12.59(8.11, 19.56) | 12.55(8.15, 19.32) | 3.64(3.02) | 12.47(8.63) | Female |
| Telaprevir | 19 | 6.36(4.05, 10) | 6.35(4.05, 9.97) | 2.66(2.03) | 6.32(4.33) | Female |
| Interferon alfa-2a | 18 | 4.17(2.63, 6.64) | 4.17(2.61, 6.67) | 2.05(1.4) | 4.15(2.82) | Female |
| Valacyclovir | 17 | 7.58(4.7, 12.21) | 7.56(4.72, 12.1) | 2.91(2.24) | 7.53(5.05) | Female |
| Stannous fluoride | 17 | 197.87(121.17, 323.14) | 186.9(116.77, 299.16) | 7.54(6.85) | 185.89(123.32) | Female |
| Afatinib | 16 | 17.04(10.41, 27.88) | 16.96(10.39, 27.68) | 4.08(3.39) | 16.88(11.18) | Female |
| Carbamazepine | 15 | 4.51(2.71, 7.49) | 4.5(2.7, 7.49) | 2.17(1.46) | 4.49(2.93) | Female |
| Terbinafine | 14 | 10.98(6.49, 18.58) | 10.95(6.45, 18.59) | 3.45(2.71) | 10.9(7.02) | Female |
| Doxycycline | 13 | 4.43(2.57, 7.64) | 4.43(2.56, 7.67) | 2.14(1.38) | 4.41(2.8) | Female |
| Rifampicin | 12 | 12.52(7.09, 22.09) | 12.47(7.06, 22.02) | 3.64(2.85) | 12.43(7.73) | Female |
| Alpelisib | 12 | 6.27(3.55, 11.06) | 6.26(3.55, 11.05) | 2.64(1.85) | 6.24(3.88) | Female |
| Cetuximab | 10 | 5.75(3.09, 10.7) | 5.74(3.07, 10.75) | 2.52(1.66) | 5.72(3.4) | Female |
| Voriconazole | 9 | 6.89(3.58, 13.26) | 6.88(3.6, 13.14) | 2.78(1.88) | 6.86(3.97) | Female |
| Trametinib | 9 | 6.26(3.25, 12.05) | 6.25(3.27, 11.93) | 2.64(1.74) | 6.23(3.6) | Female |
| Acyclovir | 8 | 5.33(2.66, 10.68) | 5.33(2.68, 10.58) | 2.41(1.47) | 5.32(2.97) | Female |
| Tucatinib | 8 | 9.04(4.51, 18.12) | 9.02(4.54, 17.91) | 3.17(2.22) | 9(5.03) | Female |
| Acitretin | 7 | 19.97(9.49, 42.02) | 19.86(9.43, 41.83) | 4.31(3.3) | 19.82(10.64) | Female |
| Iopromide | 7 | 6.12(2.92, 12.87) | 6.11(2.9, 12.87) | 2.61(1.61) | 6.1(3.28) | Female |
| **Weight** |  |  |  |  |  |  |
| Isotretinoin | 15 | 22.08(13.06, 37.33) | 21.82(13.11, 36.32) | 4.36(3.63) | 20.55(13.24) | <50 |
| Lamotrigine | 11 | 7.07(3.86, 12.95) | 7.04(3.83, 12.93) | 2.76(1.92) | 6.77(4.08) | <50 |
| Capecitabine | 10 | 14.05(7.44, 26.52) | 13.94(7.45, 26.1) | 3.75(2.87) | 13.42(7.89) | <50 |
| Methotrexate | 10 | 5.12(2.72, 9.65) | 5.11(2.73, 9.57) | 2.31(1.43) | 4.94(2.91) | <50 |
| Afatinib | 7 | 34.87(16.33, 74.48) | 34.2(16.24, 72.03) | 5.06(4.03) | 33.26(17.62) | <50 |
| Oxaliplatin | 7 | 11.4(5.36, 24.22) | 11.33(5.38, 23.86) | 3.46(2.44) | 11.03(5.87) | <50 |
| Alendronate sodium | 6 | 9.67(4.29, 21.79) | 9.62(4.31, 21.49) | 3.23(2.15) | 9.41(4.77) | <50 |
| Doxycycline | 5 | 22.56(9.25, 55.01) | 22.28(9.22, 53.82) | 4.45(3.27) | 21.85(10.36) | <50 |
| Idursulfase | 4 | 7.44(2.76, 20.03) | 7.41(2.78, 19.74) | 2.87(1.59) | 7.31(3.19) | <50 |
| Erlotinib | 4 | 18.97(7.02, 51.23) | 18.77(7.04, 50.01) | 4.21(2.92) | 18.48(8.05) | <50 |
| Trametinib | 4 | 32.16(11.87, 87.18) | 31.59(11.86, 84.17) | 4.96(3.67) | 31.09(13.5) | <50 |
| Panitumumab | 3 | 13.94(4.44, 43.72) | 13.83(4.44, 43.11) | 3.77(2.34) | 13.68(5.25) | <50 |
| Everolimus | 3 | 5.56(1.78, 17.39) | 5.54(1.78, 17.27) | 2.46(1.03) | 5.49(2.11) | <50 |
| Palbociclib | 3 | 5.26(1.68, 16.45) | 5.25(1.68, 16.36) | 2.38(0.95) | 5.2(2) | <50 |
| Omalizumab | 3 | 5.85(1.87, 18.29) | 5.83(1.87, 18.17) | 2.53(1.1) | 5.77(2.22) | <50 |
| Isotretinoin | 229 | 57.28(49.62, 66.13) | 56.08(48.89, 64.33) | 5.54(5.34) | 46.61(41.33) | 50-100 |
| Lamotrigine | 32 | 11.08(7.79, 15.74) | 11.03(7.75, 15.7) | 3.43(2.93) | 10.79(8.04) | 50-100 |
| Everolimus | 18 | 6.43(4.03, 10.24) | 6.41(4, 10.26) | 2.66(2.01) | 6.34(4.29) | 50-100 |
| Palbociclib | 18 | 3.05(1.92, 4.86) | 3.05(1.91, 4.88) | 1.59(0.94) | 3.02(2.05) | 50-100 |
| Telaprevir | 17 | 4.88(3.02, 7.87) | 4.87(3.04, 7.8) | 2.27(1.6) | 4.82(3.23) | 50-100 |
| Sunitinib malate | 15 | 4.17(2.51, 6.94) | 4.17(2.51, 6.94) | 2.05(1.33) | 4.13(2.7) | 50-100 |
| Amoxicillin | 13 | 3.51(2.03, 6.07) | 3.51(2.03, 6.08) | 1.8(1.04) | 3.48(2.2) | 50-100 |
| Interferon alfa-2a | 13 | 5.18(3, 8.95) | 5.17(2.99, 8.95) | 2.36(1.6) | 5.13(3.24) | 50-100 |
| Pantoprazole | 12 | 4.15(2.35, 7.33) | 4.14(2.35, 7.31) | 2.04(1.25) | 4.12(2.56) | 50-100 |
| Fluconazole | 11 | 11.3(6.24, 20.49) | 11.25(6.25, 20.25) | 3.48(2.66) | 11.17(6.79) | 50-100 |
| Panitumumab | 9 | 8.92(4.62, 17.2) | 8.89(4.66, 16.97) | 3.14(2.24) | 8.83(5.1) | 50-100 |
| Allopurinol | 8 | 7.34(3.66, 14.72) | 7.32(3.69, 14.54) | 2.86(1.92) | 7.28(4.06) | 50-100 |
| Valacyclovir | 7 | 6.37(3.03, 13.41) | 6.36(3.02, 13.39) | 2.66(1.66) | 6.33(3.4) | 50-100 |
| Vancomycin | 7 | 4.21(2, 8.85) | 4.2(1.99, 8.85) | 2.07(1.06) | 4.19(2.25) | 50-100 |
| Simeprevir | 7 | 12.71(6.04, 26.78) | 12.65(6.01, 26.64) | 3.65(2.65) | 12.58(6.75) | 50-100 |
| Linezolid | 6 | 4.33(1.94, 9.66) | 4.32(1.93, 9.65) | 2.11(1.03) | 4.31(2.2) | 50-100 |
| Imiquimod | 6 | 24.03(10.73, 53.82) | 23.78(10.65, 53.11) | 4.57(3.49) | 23.68(12.06) | 50-100 |
| Afatinib | 6 | 12(5.37, 26.82) | 11.94(5.35, 26.67) | 3.57(2.5) | 11.89(6.07) | 50-100 |
| Sodium fluoride | 5 | 199.06(79.56, 498.05) | 182.55(78.59, 424.04) | 7.51(6.29) | 181.87(84.43) | 50-100 |
| Eletriptan | 5 | 11.94(4.95, 28.79) | 11.88(4.92, 28.7) | 3.57(2.4) | 11.84(5.66) | 50-100 |
| Pamidronate disodium | 5 | 20.46(8.47, 49.44) | 20.28(8.39, 48.99) | 4.34(3.17) | 20.21(9.66) | 50-100 |
| Iopromide | 5 | 5.02(2.09, 12.1) | 5.01(2.07, 12.1) | 2.32(1.16) | 5(2.4) | 50-100 |
| Fenofibrate | 4 | 5.76(2.16, 15.39) | 5.75(2.16, 15.32) | 2.52(1.25) | 5.73(2.52) | 50-100 |
| Isoniazid/Pyrazinamide/Rifampin | 3 | 85.18(26.85, 270.29) | 82.03(26.84, 250.71) | 6.35(4.9) | 81.84(31.14) | 50-100 |
| Telithromycin/Clarithromycin | 3 | 11.25(3.61, 35.02) | 11.2(3.59, 34.91) | 3.48(2.06) | 11.17(4.32) | 50-100 |
| Ethinyl estradiol/Norgestimate | 3 | 81.99(25.86, 259.94) | 79.06(25.87, 241.63) | 6.3(4.85) | 78.88(30.04) | 50-100 |
| Isotretinoin | 14 | 104.16(59.23, 183.15) | 102.04(58.94, 176.65) | 6.49(5.7) | 89.63(55.89) | >100 |
| Omeprazole | 7 | 5.15(2.39, 11.06) | 5.14(2.39, 11.04) | 2.29(1.25) | 4.89(2.58) | >100 |
| Telaprevir | 3 | 9.2(2.92, 29.01) | 9.19(2.95, 28.64) | 3.17(1.73) | 8.97(3.43) | >100 |
| Ibrutinib | 3 | 10.7(3.39, 33.73) | 10.68(3.43, 33.29) | 3.38(1.94) | 10.42(3.99) | >100 |
| Perindopril erbumine | 3 | 143.06(44.65, 458.39) | 138.66(44.49, 432.17) | 7.08(5.61) | 135.04(50.97) | >100 |
| Budesonide | 3 | 5.49(1.74, 17.29) | 5.48(1.76, 17.08) | 2.42(0.98) | 5.36(2.05) | >100 |
